# The multifaceted nature of antimicrobial peptides: current synthetic chemistry approaches and future directions

**DOI:** 10.1039/d0cs00729c

**Published:** 2021-05-27

**Authors:** Bee Ha Gan, Josephine Gaynord, Sam M. Rowe, Tomas Deingruber, David R. Spring

**Affiliations:** Department of Chemistry, University of Cambridge Lensfield Road Cambridge CB2 1EW UK spring@ch.cam.ac.uk

## Abstract

Bacterial infections caused by ‘superbugs’ are increasing globally, and conventional antibiotics are becoming less effective against these bacteria, such that we risk entering a post-antibiotic era. In recent years, antimicrobial peptides (AMPs) have gained significant attention for their clinical potential as a new class of antibiotics to combat antimicrobial resistance. In this review, we discuss several facets of AMPs including their diversity, physicochemical properties, mechanisms of action, and effects of environmental factors on these features. This review outlines various chemical synthetic strategies that have been applied to develop novel AMPs, including chemical modifications of existing peptides, semi-synthesis, and computer-aided design. We will also highlight novel AMP structures, including hybrids, antimicrobial dendrimers and polypeptides, peptidomimetics, and AMP–drug conjugates and consider recent developments in their chemical synthesis.

## Abstract 2

This review discusses the diversity of structure and physicochemical properties of antimicrobial peptides and their derivatives, various chemical synthetic strategies that have been applied in their development, and how this links to their activity.

## Graphical abstract

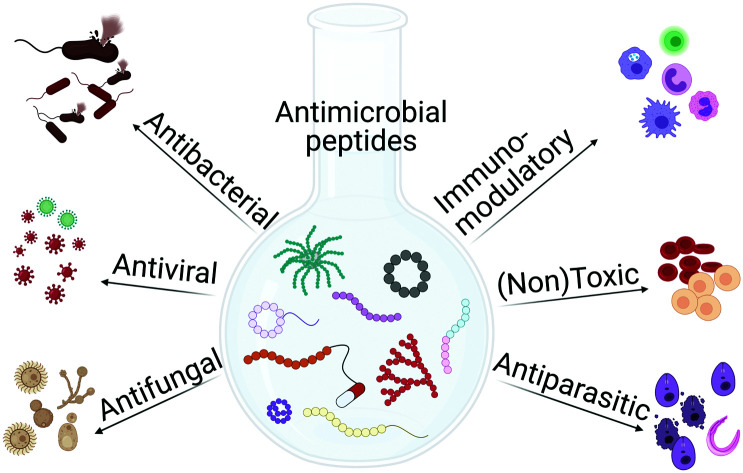

## 1 A brief history of antibiotics and the current state of play

Antibiotics are chemicals which either kill or prevent the growth of microbes. The word ‘antibiotic’ means ‘anti-life’, however it has become somewhat synonymous with antibacterial agents.^1^ Today the term antimicrobial is often used in place of antibiotic to emphasise the inclusion of antiviral, antifungal and antiparasitic agents, although both terms can be used interchangeably and will be used as such throughout. When appropriate, the specific terms, *e.g.* antibacterial, antifungal, will be used. Most of the antimicrobial research has focussed on antibacterial agents and therefore much of the discussion presented here will focus on this subclass. However, we direct the reader to Section 16 for a dedicated discussion of other subclasses.

Humankind has employed antimicrobials for millennia with documented examples of herbs, honey, and mouldy bread being used in ancient Egypt, China, and Rome to treat infections.^2^ While developing dyes for bacterial stains in the early 20th century, Paul Ehrlich observed that some compounds displayed an antibacterial effect. This inspired the search for a ‘magic bullet’ – a drug which would selectively kill disease-causing organisms while sparing the human patient. This search culminated in the discovery of arsphenamine in 1909: the first synthetic antimicrobial agent.^3^ Marketed as ‘Salvarsan’ in 1910, arsphenamine was the first effective treatment for syphilis, which had been one of the largest public health burdens in the 16th through 19th centuries. Sir Alexander Fleming's serendipitous discovery of penicillin G in 1928 was the next significant milestone in the history of drug discovery. The translation of Fleming's research into an extremely effective, mass-produced medicine saved thousands of lives, leading to penicillin being known as ‘the wonder drug’. This breakthrough triggered a race to develop similarly effective antimicrobials and has ultimately led to the drug discovery landscape we see today.^4^ In 2019, the WHO published the 21st Essential Medicines List which includes over 40 antimicrobials.^5^ These drugs can be sourced directly from nature; synthesised from simple building blocks in a laboratory; or a combination of the two whereby a complex molecule is sourced from nature and then further synthetically modified (semi-synthesis). Some of the major classes of antibiotics, which have been reviewed extensively elsewhere, are listed in Table 1.^1,2,7^

**Table 1:** An overview of the major classes of antibiotics, with key examples, targets, and sources. Adapted from Brown and Wright^11^

Class	Example	Target	Source
Sulfonamides	Sulfanilamide	Folate synthesis	Synthetic
Fluoroquinolones	Ciprofloxacin	DNA topoisomerases	Synthetic
β-Lactams	Ceftazidime	Cell wall synthesis	Natural product
Oxazolidinones	Linezolid	Protein synthesis	Synthetic
Aminoglycosides	Neomycin	Protein synthesis	Natural product
Glycopeptides	Vancomycin	Cell wall synthesis	Natural product
Polymyxins	Polymyxin B	Bacterial cell membrane	Natural product
Cyclic lipopeptides	Daptomycin	Bacterial cell membrane	Natural product
Tetracyclines	Tetracycline	Protein synthesis	Natural product

Despite increasingly sophisticated approaches to antimicrobial discovery and development, these drugs have several common limitations. Among the most restrictive is poor bioavailability, which necessitates regular and high dosing in order to maintain a sufficient concentration of drug at the site of infection.^6^ Another major issue is systemic toxicity, which is inherent for some classes of antibiotics. For example, nephro- and neuro-toxicity limit the clinical use of polymyxins, the last-resort drugs for *Pseudomonas aeruginosa* infections.^7^ The US Food and Drug Administration (FDA) have also recently updated their guidance for the use of fluoroquinolone antibiotics due to the possibility of life-threatening side effects in patients with low blood sugar.^8^ Broad-spectrum antibiotics, which are able to kill multiple species of microorganism, enable the rapid treatment of undiagnosed infections. However, over-reliance on these ‘catch-all’ therapeutics has become increasingly recognised as a significant contributor to the growing antimicrobial resistance (AMR) crisis. Furthermore, since the targets of these antibiotics are conserved among multiple species, they risk harming non-pathogenic organisms of the microbiome. This can result in significant side effects, particularly for patients with additional health conditions.^6,9^ Narrow-spectrum or ‘precision’ antibiotics can mitigate these risks by enabling individual species to be selectively targeted, but reliable, time-consuming, and costly diagnostic tests are required to determine a suitable drug for treatment.^10^

In this review, we aim to provide a broad introduction to antimicrobial peptides (AMPs) from the perspective of chemical synthesis, which we believe has not been adequately addressed in the current literature. For this reason, we have adopted a broad definition of an AMP, which will be outlined in Section 3. This review is not comprehensive of all AMPs and instead aims to cover the most interesting synthetic landscape. Where relevant, we direct the reader towards other insightful reviews already available in the literature.

## 2 Bacterial survival mechanisms

### 2.1 Bacterial resistance

AMR is a serious global threat to human, animal, and environmental health. In terms of human health, its effects are being felt acutely in the fields of surgery, transplantation, and infection treatment.^12^ There are several key factors contributing to the increasing spread of AMR among microbe populations. Among the most significant is the excessive usage of broad-spectrum antimicrobial products. For example, excessive antimicrobials have been added to animal feed as a preventive measure in farming. In humans, it is due to the dispensing of antibiotics, which are widely available without medical prescriptions in certain countries.^13^ Increased international travel, poor sanitation and hygiene, and the release of non-metabolised antibiotics into the environment through manure and faeces are also contributing factors.^14–16^

**Fig. 1:**
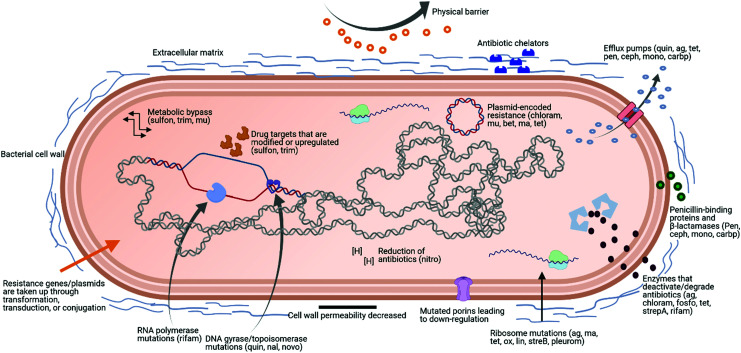
Scheme of possible bacterial mechanisms of resistance. Some examples of antibiotics affected by different mechanisms of resistance are shown in brackets. ag: aminoglycosides, ma: macrolides, tet: tetracyclines, ox: oxazolidinones, lin: lincosamides, strepA: streptogramin A, strepB: streptogramin B, pleurom: pleuromutilins, quin: quinolones, nal: nalidixic acid, novo: novobiocin, sulfon: sulfonamides, trim: trimethoprim, mu: mupirocin, chloram: chloramphenicol, fosfo: fosfomycin, rifam: rifamycins, nitro: nitroimidazoles, pen: penicillins, ceph: cephalosporins, mono: monobactams, carbp: carbapenems, bet: β-lactams.

A particularly concerning facet of AMR is multidrug resistance (MDR), which is defined as acquired non-susceptibility to at least one drug in three or more antimicrobial classes.^17,18^ Microorganisms displaying MDR are typically placed in one of two categories. The first category comprises pathogens that belong to the same genera and species as normal human commensal flora, but have acquired antibiotic resistant genes and are more virulent.^18^ Methicillin-resistant *Staphylococcus aureus* (MRSA), vancomycin-resistant enterococci (VRE), and drug-resistant *Escherichia coli* (*E. coli*) are examples of this class. The second category, known as ‘opportunistic pathogens’, are environmental bacteria that only display pathogenicity in an immunocompromised host. *Pseudomonas aeruginosa*, *Stenotrophomonas maltophilia*, *Acinetobacter baumannii*, and *Burkholderia cepacia* belong to this class.^18^ In 2017, the WHO identified *Pseudomonas* spp., *Acinetobacter* spp., and *Enterobacteriaceae* as the three groups of pathogens with the most critical need for new antibiotics. These pathogens were identified as displaying widespread MDR and posing a threat to hospitals, nursing homes, and to patients who require devices such as ventilators and catheters. These bacteria have become resistant to numerous conventional antibiotics, including carbapenems and third generation cephalosporins, which are currently the best antibiotics for treating MDR bacteria. Fig. 1 shows some examples of antibiotics that could be affected by different bacterial mechanisms of resistance, based on the review from Sriramulu.^19^

### 2.2 Bacterial tolerance and persistence

There are numerous bacterial survival mechanisms that have led to diverse nomenclature, which has been summarised by Mathias and co-workers.^20^ Here, we would like to draw attention to two modes of action other than bacterial resistance, which are known as tolerance and persistence. These terms describe two phenomena in which many conventional antibiotics fail to exhibit antibacterial effects, leading to increased bacterial survival.^21–24^

Bacteria that are tolerant can survive in the presence of antibiotics whose concentration is above the minimum. These bacteria cannot multiply in the presence of the antibiotic and arise without undergoing genetic change. However, they are killed at slower rates than resistant bacteria.^21,25^

Persistent bacteria have similar characteristics to tolerant bacteria. However, persistence concerns only a subpopulation of bacteria exposed to the antibiotic. In other words, if 100% of the population are persistent, they are tolerant. Persister cells are absent in exponentially growing cells. They can be found when the bacteria are under various forms of stress, for example in a stationary-phase culture (about 1%) and in biofilms.^25–28^

Due to their metabolically dormant state, persister cells are not sensitive to conventional antibiotics such as fluoroquinolones, aminoglycosides, and β-lactams, which are effective against actively growing cells. Effective antibacterial compounds must enter the persister cells without utilising bacterial machinery, which is likely to be inactive due to the dormant state.^25^ For an in-depth understanding of bacterial survival mechanisms and their evolution in the presence of antibiotics, we direct the reader to the recent reviews by Mathias and co-workers and Hardt and co-workers.^20,21^

Within the current arsenal of antibiotics, only a limited number of structural classes and targets are represented, with little evidence of any anticipated change in the current drug pipeline. Since 2000, only six new classes of antibiotics have been brought to market, and although several of these drugs have novel mechanisms of action, they only target Gram-positive bacteria.^29,30^ Four out of the six ESKAPE pathogens, recognised for their concerning levels of MDR and virulence, are Gram-negative. This is a niche that current therapies are failing to fill.^31^ The term ‘ESKAPE’ comprises six highly virulent and antibiotic resistant bacterial pathogens: *Enterococcus faecium*, *Staphylococcus aureus*, *Klebsiella pneumoniae*, *Acinetobacter baumannii*, *Pseudomonas aeruginosa*, and *Enterobacter* spp. For the future of human healthcare, it is imperative that new drugs are developed that are capable of overcoming the challenges posed by Gram-negative bacteria, and that can perturb the development of MDR.^6,32^

Numerous organisations and government entities are united in calling for increased investment in antibiotic discovery. Aside from traditional small-molecule drugs, recommendations have been made for the investigation of non-traditional and alternative therapies, focussing on different bacterial targets and more unusual molecular architectures.^32,33^ The UK Government Review on AMR specifically highlights AMPs as promising alternative therapeutics that merit investment and research.^34^ Likewise, recent studies have revealed that AMPs form a class of antibiotics that have low propensity to develop resistance.^35–37^

## 3 Antimicrobial peptide classification and the antimicrobial peptide database

AMPs, many of which are also referred to as host defence peptides (HDPs), are a numerous and varied group of molecules. For the purposes of this review, we will adopt a broad definition of AMPs, considering them to be peptides composed of predominantly α-amino acids that display antimicrobial activity, or that facilitate the antimicrobial activity of other compounds *e.g.* peptide efflux pump inhibitors. This means that they need not function by membrane disruption, which is classically considered a defining feature of many AMPs.^38,39^ We also consider that AMPs can be anywhere from two to 100 residues in length, can be charged or uncharged, and can be made by cellular machinery, fully synthetic, or a combination of the two. They can also be adorned with various modifications (*e.g.* lipid chains, PEG chains, sugars), meaning that anionic/neutral peptides and glycopeptides can be included in this discussion, despite the fact such compounds are not often considered as AMPs elsewhere in the literature.^40–43^ While the peptide-derived β-lactam antibiotics are AMPs by this definition, they will not be considered in this review. Furthermore, our definition means that some AMPs discussed (*e.g.* vancomycin, polymyxins) are not the ‘alternative therapeutics’ highlighted as requiring further investigation by the UK Government Review on AMR and are actually some of the most well-established clinical antibiotics.^44,45^ However, by using this broad definition of AMPs, we can showcase the diversity of peptide structures that exhibit antimicrobial activity and discuss the variety of methods reported in the literature for their chemical synthesis.

Numerous databases exist for documenting AMPs, and the antimicrobial peptide database (APD) is one such example which was originally created as a response to growing interest in AMPs as therapeutic agents against MDR pathogens.^46^ The database is dedicated to the ‘glossary, nomenclature, classification, information search, prediction, design, and statistics of AMPs and beyond’, with data having been collected manually from PubMed, the PDB, Google, and Swiss-Prot. Since its creation in 2003, the database continues to be updated and expanded by the Wang laboratory and is currently in its third iteration: the APD3.^46–48^ It is far from the only database of AMPs, but it is one of the most general in its scope, and therefore falls closest in-line with what we wish to highlight in this review. We direct the reader to a list of other AMP databases on the APD website should they wish to find more.

**Fig. 2:**
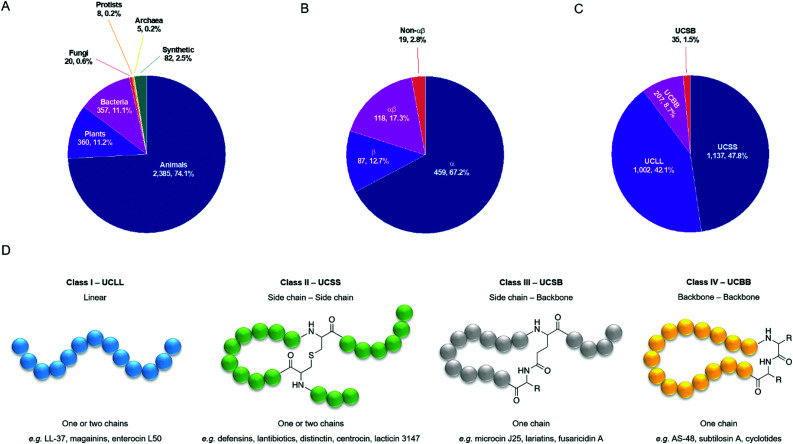
(A) Sources of the 3217 antimicrobial peptides in the APD3 database as of July 2020. (B and C) Structural classes of AMPs in the APD3 as of July 2020 using the α-helix/β-sheet system and using the universal classification system (UCS). Only those AMPs with the reported structural data necessary for classification have been included. (D) Classification of AMPs based on connection patterns of the polypeptide chain.^51^ An example linkage is used for class II and class III. The exact chemical nature of the linkage can vary. R is an aa side chain.

Typically, four criteria are followed to register a peptide in the APD3: (1) it must be from a natural source; (2) antimicrobial activity must be demonstrated (MIC < 100 mM or < 100 mg ml^−1^; (3) the amino acid (aa) sequence of the mature peptide must have been elucidated, at least partially; and (4) the peptide must contain fewer than 100 aa residues. However, approximately 2.5% of the entries are synthetic peptides of interest, and since October 2012 the database also includes some small yet important antimicrobial proteins (>100 aa). As of July 2020, there were 3217 AMPs in the database with entries from all six kingdoms of life (Fig. 2A).^49^ The database is designed to reduce redundancy, and so the same AMP from different species will share an entry, as will synthetic fragments of natural AMPs and the peptide from which they are derived.^50^ The APD3 has been used to describe two different structural classification systems, the first of which focusses on the presence of α-helix and/or β-sheet secondary structure elements. Using this α-helix/β-sheet system, AMPs are classified into four families: α, β, αβ, and non-αβ. AMPs in the α-family contain an α-helix as the main structural element, whereas those in the β-family contain at least a pair of β-strands. αβ-Structures have a mixture of both elements and non-αβ structures have neither. The percentage of AMPs in the database belonging to each of these four structural classes can be found in Fig. 2B, with the majority belonging to the α-family (459, 67.2%). This classification system relies on knowledge of AMP 3D structure, which is provided either by solution NMR or X-ray diffraction studies. Of the 3217 entries in the APD, only 683 (21.2%) have a reported 3D structure and so can be classified by their α-helix/β-sheet content.

To account for the AMPs in the APD3 that do not have a reported 3D structure (78.8%), Wang proposed a universal classification system (UCS) based on the covalent bonding patterns of polypeptide chains (Fig. 2D).^51^ The classification system has four classes, the first of which, UCLL (LL = linear), includes all linear peptides where, if chemical modifications are present, they are confined to individual aa. This is in contrast to the second class, UCSS (SS = side chain–side chain), in which all peptides have at least one chemical bond between different aa side chains, regardless of whether those aa are part of the same peptide backbone. The third class, UCSB (SB = side chain–backbone), contains peptides with a bond between the side chain of one residue and the backbone of another residue. The final class, UCBB (BB = backbone–backbone), comprises all those peptides that are cyclised head-to-tail. Several of these classes can be further sub-divided based on the number of polypeptide chains present and the nature of linkages present. We direct the reader to Wang's original outline of this UCS for further discussion.^51^ Using the UCS, a significantly greater proportion of entries in the APD3 have been classified by their structure (2381, 74.0%) (Fig. 2C). Most of these entries fall into the first class, UCLL (1002, 42.1%), or the second class, UCSS (1137, 47.8%).

Although the UCS can be applied universally, it does not emphasise all important structural features. For example, in the UCS lasso peptides fall into the category UCSB, indicating that they contain a side chain to backbone connection. However, this classification does not indicate their most interesting structural feature: the ring formed by the side chain–backbone connection is threaded by the resulting ‘tail’. It is this threading that affords the eponymous ‘lasso’-like structure that is responsible for many of their interesting properties.^52^ Nor does a classification of UCSS distinguish between the lantibiotic nisin, which has an extended structure, and the lantibiotic cinnamycin, which is globular. From a chemical synthesis perspective, these lasso peptides and lantibiotics are some of the most interesting and challenging natural peptide structures to realise.^53^ Despite this challenge, they are attractive therapeutic scaffolds due to their high stability, which is a consequence of their complicated topologies.^54,55^ Using either the UCS or α-helix/β-sheet systems, these interesting and important structural properties may easily be overlooked. However, given the extremely diverse structures of AMPs, no classification system can effectively highlight all key structural features while remaining broad in scope. While these two classification systems have been discussed in the context of AMPs, they can equally be applied to peptides in general.

**Fig. 3:**
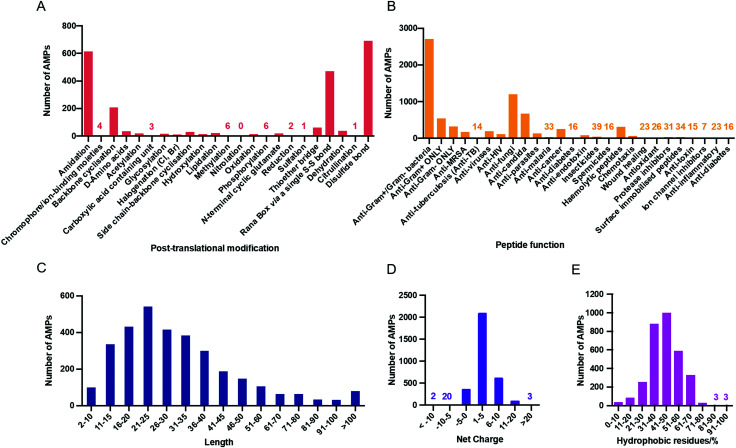
(A) AMPs with post-translational modifications reported in the APD3 as of July 2020. This data was collected from the APD3 by searching in the ‘chemical modification’ box with each of the 23 search terms corresponding to the modification, as outlined by Wang.^51^ (B) The number of AMPs in each of the 25 searchable peptide functions reported in the APD3 as of August 2020. Physicochemical properties of AMPs in the APD3 as of July 2020: aa length (C), net charge (D), and hydrophobicity (E).

Contributing to the diversity of AMPs are the numerous post-translational modifications (PTMs) which they can undergo. PTMs play important structural and functional roles and so the APD3 enables the user to search for AMPs with 23 types of PTM. The number of AMPs in the APD3 containing these modifications is shown in Fig. 3A.^56^

One of the most common PTMs in the database is C-terminal amidation, which can be critical for antimicrobial activity.^57^ Amidation raises the net charge of a peptide by +1 through neutralisation of the C-terminal carboxylate that would otherwise be present. In addition, amidation can alter the helicity of the AMP.^58^ For the synthetic AMP modelin-5, both amidated and un-amidated analogues can bind to lipid bilayers, but upon binding, helix formation is only induced in the amidated analogue.^59^ However, while amidation can improve antimicrobial activity, for some AMPs little difference is observed with or without amidation.^60^ In contrast to amidation, N-terminal acetylation is uncommon in natural AMPs, despite being used frequently in synthetic AMPs. This could again be related to net charge, as acetylation masks the positive charge afforded by a free N-terminal α-amino group at physiological pH.

In addition to antimicrobial activity, AMPs display a range of other functions and so the APD has been continuously updated to reflect these properties. In its current format, the APD3 offers 25 searchable peptide activities which are listed in Fig. 3B along with the number of peptides known to display each of those properties. Another means of AMP classification is by their physicochemical properties. This is an important source of information for designing synthetic AMPs and for drawing correlations between these properties and proposed mechanisms of action.

## 4 Physicochemical properties of AMPs

The lengths of peptides in the APD3 range from 2 to 183 aa residues, with an average length of 33.27 residues (Fig. 3C). The upper limit is however arbitrary and is purely defined by the scope of peptides collected in the database. There are two AMPs in the database that are two residues in length, gageotetrin A and peptide F3, both of which are post-translationally modified with lipidation and biphosphonation respectively. The shortest AMPs in the database to show antimicrobial activity without PTM are five residues long, several of which are from the antibacterial vermipeptide family. However, these are the extremes and most AMPs (88.5%) in the database are between 11 and 60 residues in length.

AMPs are typically cationic at neutral pH which can help to direct them to negatively charged bacterial membranes by electrostatic attraction (See Section 6.1).^61^ However, there are still many examples of neutral and anionic AMPs. The APD3 currently reports 195 AMPs with a negative net charge, 188 with a net charge of zero, and 2834 with a positive net charge (Fig. 3D). The breadth of known net charges is impressive with the ‘most anionic’ AMP, chrombacin, having a −12 net charge, while the ‘most cationic’ AMPs, sheep cathelicidin OaBac11 and fish histone-derived Oncorhyncin II, have a +30 net charge. The overwhelming majority of AMPs though (96.2%) have a net charge of between -5 and +10, with an average net charge of +3.33. Unlike some other databases, the APD3 takes the effect of chemical modification on net charge into account.^50^ For example, the net charge of AMPs that are amidated at the C-terminus is increased by +1.

The range of hydrophobicities of AMPs in the database is also broad with examples at both extremes (Fig. 3E). For example, baceridin (WaiVlL, cyclic) is composed exclusively of hydrophobic residues, therefore displaying 100% hydrophobicity. Gramicidin A (CHO-VGAlAvVvWlWlWlW-NH(CH_2_)_2_OH) and B (CHO-VGAlAvVvWlFlWlW-NH(CH_2_)_2_OH) also display notably high hydrophobicity at 93%. Most AMPs in the APD3 (98.6%) have a hydrophobicity of between 10 and 80%, with the peak of the distribution located between 40 and 50%. At the other end of the spectrum, sheep anionic peptide contains no hydrophobic residues at all. Throughout this review, note that l- and d-amino acids are assigned uppercase and lowercase letters, respectively (*i.e.* “V” for l-Val, and “l” for d-Leu).

Using the APD3, it is also easy to investigate the average length and net charge of AMPs with respect to their universal structural class (Table 2). The two properties do not directly correlate and while the average length follows the sequence UCSS1a > UCBB > UCSS1b > UCLL > UCSB, the order for average net charge is UCSS1a > UCLL > UCSS1b > UCBB > UCSB. UCSS1a peptides have a side chain-to-side chain disulfide bond, whereas for UCSS1b peptides it is a thioether bond. It is also interesting to consider the different aa compositions of each of these structural classes and we direct the reader to a review by Mishra and Wang in 2012 that addresses this issue.^62^ Together, these observations facilitate the prediction of AMP activity for a given sequence and furthermore, and help to inform the design of new AMPs.

**Table 2:** Number, average length, and net charge of AMPs from each of the universal structural classes in the APD3 as of July 2020

	UCLL	UCSS1a	UCSS1b	UCSB	UCBB
Number of AMPs	1002	1065	72	35	207
Average length	24.77	40.06	27.68	17.37	29.77
Average net charge	2.75	4.18	1.65	0.03	1.10

The conclusions drawn here about key physicochemical properties of AMPs map closely to those previously drawn by Wang and co-workers in 2015, despite an additional 598 AMPs having been added to the database (a 23% increase). This suggests that the boundaries and distribution of the properties described provide a solid framework to guide our understanding of this diverse range of molecules (typical length: 11 to 60 residues; charge: +1 to +5; hydrophobicity: 30% to 70%). However, the number of natural AMPs is likely in the range of millions, and so the database is far from a fully representative sample. Nonetheless, it is an extremely useful tool and will continue to increase in utility as the number of entries grows.^50^

## 5 Influence of environmental factors on AMPs

### 5.1 Effect of salt concentration on AMPs

It has been widely reported that many AMPs display lower activity in environments of high salt concentration, including clavanins, tachyplesin, histatins and defensins.^63–66^ This is of particular therapeutic relevance for cystic fibrosis (CF) patients, who are at risk from opportunistic pathogens like *P. aeruginosa*, and who often suffer from chronic infections in the pulmonary mucus, which typically has an elevated salt concentration. The Cl^−^ concentration of trachea and main stem bronchi airway surface liquid (ASL) of CF patients has been measured at around 129 and 170 mM respectively (compared to 84 and 85 mM for normal ASL).^67^ In CF patients, it has been demonstrated that the activity of AMPs is much lower in the ASL of CF patients compared to normal ASL.^67,68^ As many AMPs have been shown to be salt-sensitive *in vitro* (that is, they display lower activity in higher salt concentrations), it is believed that this could be the reason for their lack of efficacy in CF patients.^69^

Many membrane-disrupting AMPs adopt an α-helical, amphipathic structure to exert their antibacterial activity. A peptide helix features multiple backbone hydrogen bonds between the carbonyl oxygen and amide NH of consecutive helical turns, and these interactions can vary depending on the local environment, such as ionic strength and pH. There is also evidence that the local ionic strength does not just affect the structure of the peptide but can also interfere with the peptide–lipid interactions. Larson and Kandasamy used molecular dynamics (MD) simulations to study the effect of salt concentration on the interaction of AMP magainin and palmitoyloleoylphosphatidylcholine bilayers and found that the interactions between the peptide and lipids were stronger at lower salt concentrations.^70^

Various strategies have been employed to increase the activity of AMPs in the presence of high salt concentrations, including many of the synthetic modifications that will be discussed in this review. These approaches have been used for natural and *de novo*-designed AMPs, and examples of both will be given in this section.

The simplest approach is to modify the aa sequence of a natural AMP. However, this has been found to have mixed results. Mor and co-workers developed truncated analogues of frog-derived peptide dermaseptin S4 (sequence H-ALWMTLLKKVLKAAAKAALNAVLVGANA-NH_2_) and investigated their activities against *E. coli* under different incubation conditions.^71^ It was found that a shortened 14-mer analogue with a Met-Lys substitution (sequence H-ALWKTLLKKVLKAA–NH_2_) lost activity as the concentration of NaCl was increased, whereas a longer 28-mer analogue did not. Hancock and co-workers investigated analogues of a hybrid peptide of insect cecropin and bee melittin (sequence H-KWKLFKKIGIGAVLKVLTTGLPALIS-NH_2_) which differed in hydrophobicity and net positive charge. It was observed that the activity of these analogues against *P. aeruginosa* was unaffected under higher NaCl concentrations.^72^ Aside from modifying existing AMPs, *de novo* designed AMPs can be salt resistant.^73,74^ For example, Mietzner and co-workers developed *de novo* designed amphipathic AMPs comprised of Val and Arg residues, which differed in length and the position and number of Trp residues.^73^ In general, the inclusion of several Trp residues rendered the peptides insensitive to the presence of 150 mM NaCl when the activities were measured against *P. aeruginosa* and *S. aureus*.

Moving away from simple aa substitutions and towards the production of antimicrobial peptidomimetics (AMPMs, see Section 14), it has been shown that altering the stereochemistry of aa residues can improve salt sensitivity.^75^ Similarly, the replacement of natural aa with bulky, unnatural aa has the same effect. Cheng and co-workers demonstrated the success of this approach with P113, a peptide derived from AMP histatin 5. The authors replaced Trp and His residues with β-naphthylalanine and β-(4,4′-biphenyl)alanine residues, and these aa replacements recovered the normal antimicrobial activity against bacteria including *E. coli*, *P. aeruginosa* and *S. aureus*.^76^ The same group applied this strategy to Pac-525, a short Trp-rich AMP (sequence Ac-KWRRWVRWI-NH_2_), and produced an AMPM where all of the aa were the D stereoisomer, and the Trp residues were replaced with d-β-naphthylalanines. This AMPM retained antifungal activity against multiple fungal species even at high salt concentrations.^77,78^

Lehrer and co-workers identified that two intramolecular disulfide bonds present in protegrin PG-1 were necessary to maintain antibacterial activity in the presence of 100 mM NaCl.^79^ Under the same conditions, a linear analogue displayed negligible bactericidal activity. Therefore, an obvious strategy to confer salt resistance is to macrocyclise linear AMPs. Doing just this, Yang and co-workers produced constrained analogues of tachyplesin, which contains an intramolecular disulfide bond. The constrained analogue displayed a β-sheet secondary structure, and retained broad spectrum activity in both high and low salt concentrations.^64^ Other, more drastic structural changes can be performed, for example dimerisation. Shin and co-workers investigated two different strategies for dimerising a model cationic AMP, either *via* the side chain of a Lys residue, or *via* disulfide bond formation.^80,81^ For the disulfide-linked dimers, analogues where the Cys residue was placed at either the N- or C-terminus (sequences (H-CKLKKLWKKLLK-NH_2_)_2_ and (H-KLKKLWKKLLKC-NH_2_)_2_ respectively) were resistant to 150 mM NaCl. Unlike the monomer, the Lys-linked dimer (sequence (H-KLKKLWKKLLK)2K-NH_2_) retained activity in the presence of 150 mM NaCl against a range of Gram-negative and Gram-positive bacteria. The authors hypothesised that this resistance is due to the multimeric oligomerisation of the dimeric peptides under high salt concentration.^82^

For many peptides, the antimicrobial activity is dependent on their helical content. Kim and co-workers found that the helical contents of a model peptide [RLLR]_5_ (sequence = H-RLLRRLLRRLLRRLLRRLLR-NH_2_) and magainin II were greatly diminished in the presence of 200 mM NaCl, which was accompanied by a loss in antimicrobial and antifungal activity.^83^ The authors incorporated helix-stabilising sequences at the N- and C-termini of the peptides, (sequence: H-APKAMRLLRRLLRLQKKGI-NH_2_), which resulted in capped [RLLR]_5_ and magainin II maintaining helicity in the high salt environment, and losing no activity against both Gram-positive and Gram-negative bacteria, as well as fungi.

Most literature reports on the effect of salt concentrations on AMPs focus on NaCl, as it is of high clinical relevance for CF. It has been shown that increasing the concentration of divalent cations (Zn^2+^, Ca^2+^ and Mg^2+^) can increase the antimicrobial activity of AMPs such as kappacin and DCD-1L (see Section 6.1); however, there are conflicting reports as to the whether these cations are present in significantly higher concentrations in CF patients.^84–87^

From the examples given here, it is clear that a wide range of structural modifications can be successful in reducing the salt sensitivity of AMPs; however, the current information is highly specific for each peptide in question and the target bacteria. More in-depth mechanistic information is required to explain why AMPs lose activity in high salt environments, which would help to develop over-arching design guidelines for future AMP therapeutics, and could identify novel structural modifications that have not yet been applied to AMP design.

### 5.2 Influence of pH on the biological activity of AMPs

In addition to salt sensitivity, the biological activity of AMPs is also influenced by the environmental pH. Phoenix and co-workers have summarised the potential of pH-dependent AMPs derived from natural sources as therapeutic agents.^88^ Generally, the activity of AMPs can vary depending on the therapeutic site, the pH of which will not necessarily be at physiological pH (pH 7.4). As such, it will affect the AMP's physicochemical properties, and therefore potentially its mechanism of action. For example, sites of local inflammation, such an abscess, are characterised by an acidic milieu due to a local increase of lactic acid and fatty acid by-products from bacterial metabolism.^89,90^ The skin is another environment where acidity (pH 4–6) plays a critical role in inhibiting bacterial growth and promoting would healing.^91,92^

Changes in the environmental pH can affect the interaction between AMPs and bacterial membranes by altering protonation states of the AMP's functional groups. pH can also affect the nature of the bacterial surface itself, which will affect peptide binding.^93^ Most studies that investigate the effect of pH on AMP activity focus on a pH range from 5.5 to 7.5.^94,95^ It has been noted that histidine-rich peptides typically exhibit better antimicrobial activity at low pH due to the increase in net positive charge in acidic condition.^96^ At physiological pH, most histidine (p*K*_a_ 6.5) would be present as the neutral species. However, at a low pH, much more histidine would be present as the positively charge protonated species, which can enhance the ability of AMPs to interact with anionic bacterial membranes.^63,88,94^ pH is also known to impact the activity of anionic peptides, with acidic conditions increasing the overall positive charge.^84,97^ By testing the antimicrobial activity over a wider range of pH values (pH 4–9), a study from Wimley and co-workers showed that AMPs generally exhibit a linear decrease in antimicrobial activity against Gram-negative bacteria (*E. coli* and *P. aeruginosa*) with increasing pH. In contrast, the same study observed the opposite trend for the Gram-positive bacterium *S. aureus*, where high pH led to increased antimicrobial potency.^98^ This effect was attributed to the different protonation states of native charged molecules in the peptidoglycan of Gram-positive bacteria at higher pH, in which case, the charged molecules did not interfere with the diffusion of the AMPs across the peptidoglycan layer to reach the bacterial inner membrane.^98^ Another study by Welsh and co-workers demonstrated that the antibacterial activity of β-defensin-3 and LL-37 was impaired against *S. aureus* and *P. aeruginosa* in acidic pH and reduced the synergistic activity that is typically observed when these AMPs are used in combination. Instead, β-defensin-3 and LL-37 displayed better antibacterial activity against *S. aureus* at basic pH, where both peptides are less positively charged which may facilitate the insertion into the membranes.^99^

Nevertheless, the effect of pH on the antimicrobial activity of AMPs is strongly dependent on the AMP sequence as well as its mechanism of action and the bacterial cell wall composition. Modulating environmental pH to influence the protonation states of AMPs and charged molecules in microbial cell walls/membranes can have direct influence on the initial binding of a membrane disruptive AMP, which is critical for its activity. As such, pH modulation of AMPs can be considered as a strategy to combat common resistance mechanisms, and increase the potency of AMPs.^88,93,100^ However, such a strategy might be carried out only in topical applications.^93^

## 6 AMP mechanisms of action

An understanding of the mechanisms of action of AMPs is essential in order to make informed decisions when designing new AMP therapeutics. Multiple studies have highlighted two overarching mechanisms of action: membrane disruption and immunomodulation. However, this does not imply that other mechanisms of action are not employed by AMPs. For example, the glycopeptide vancomycin primarily acts by inhibiting the polymerisation and cross-linking of the cell wall peptidoglycan instead of disrupting bacterial cell membranes.^101^

### 6.1 Membrane disruption

**Fig. 4:**
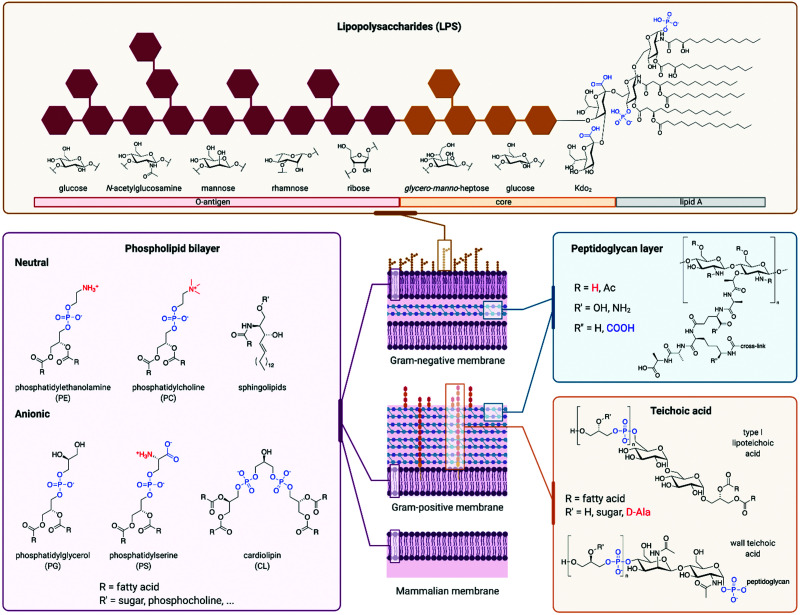
Different components of cellular envelopes. The charge of the different structures is highlighted, with negatively charged groups being shown in blue and positively charged groups in red. Phospholipid building blocks are found in cell membranes of mammals and bacteria: the surface of bacterial membranes contains a higher proportion of anionic phospholipids like phosphatidylglycerol, phosphatidylserine or cardiolipin, but it also contains neutral phosphatidylethanolamine. Mammalian cells contain a higher proportion of neutral phospholipids like phosphatidylcholine or sphingolipids. In addition to the cell membrane, bacteria have a cell wall consisting of peptidoglycan, which is mostly neutral. The cell wall of Gram-positive bacteria consists of a thick layer of peptidoglycan, which also contains teichoic acid with negatively charged phosphate units. Gram-negative bacteria have a thinner layer of peptidoglycan for their cell wall, which is protected by additional outer phospholipid bilayer. The surface of this outer membrane is covered with lipopolysaccharides (LPS). LPS consist of a chain of saccharide units, which are anchored to the membrane by the lipid A subunit. Saccharides in the core and O-antigen parts of LPS, as well as saccharides and fatty acids of lipid A, vary between different strains of bacteria, hence the structures shown are only a representative sample.

AMPs whose mechanism of action involves membrane disruption typically rely on the different compositions of host and pathogen membranes to impart selectivity. The surface of mammalian cells is mainly composed of neutrally charged phospholipids such as sphingolipids or phosphatidylcholine (Fig. 4).^102^ On the other hand, a significant fraction of bacterial membranes consists of negatively charged phospholipids such as phosphatidylserine (PS), phosphatidylglycerol (PG), and cardiolipin.^103^ In addition, the peptidoglycan cell wall that surrounds the cell membrane of Gram-positive bacteria contains significant quantities of negatively charged teichoic acid, while the outer leaflet of the outer membrane of Gram-negative bacteria is largely composed of negatively charged lipopolysaccharide (LPS).^104–106^ Mammalian cell membranes also contain negatively charged phospholipids like PS, but they are mostly located in the cytosolic leaflet of the bilayer.^102^ In some cancer cell lines, membrane regulation is lost, resulting in an increased presence of negative phospholipids on the cell surface.^107^ In these cases, some AMPs are able to selectively target the cancer cells over normal mammalian cells, illustrating the importance of membrane composition for AMP selectivity.^108^ Unlike bacterial membranes, mammalian membranes also contain the uncharged steroid cholesterol, which reduces their fluidity. This decreased fluidity has been implicated in reducing the membrane rearrangement often caused by AMPs.^109^ The overall negative charge of bacterial outer surfaces is likely responsible for the cationic nature of most AMPs, which have evolved as a defence mechanism against bacteria. The resulting electrostatic attraction facilitates initial peptide binding.^110^ It has been shown that decreasing the positive charge of an AMP below a certain threshold can significantly lower its activity.^111^ However, while increasing the positive charge typically leads to improved antimicrobial activity, too high a charge leads to an increase in off-target toxicity. The increased charge density is thought to enhance the interaction between water and the AMPs, stabilising the hydrophilic surface of transmembrane pores formed by AMPs after the initial binding event. However, many AMPs are anionic, in which case electrostatic attraction to a negatively charged membrane does not seem like a viable mechanism at first sight. An example of such peptide is DCD-1L.^97^ Despite its overall negative charge of −2, it has been proposed that only its positively-charged N-terminus is involved in initial membrane interaction. In addition, divalent cations such as Zn^2+^, Ca^2+^, or Mg^2+^ were found to improve antimicrobial activity, possibly through stabilisation of membrane-spanning DCD-1L oligomers, or by forming a salt bridge between the anionic peptide and anionic phospholipids. Other reported mechanisms for anionic AMPs rely on their hydrophobicity or the presence of positive phospholipids to facilitate the membrane interaction.^112^ Some anionic AMPs bypass membrane attachment and are taken up by cells *via* transporter proteins, such as microcin J25 using the outer membrane protein FhuA.^113^

The majority of AMPs are also amphipathic in nature, with an average hydrophobicity of 50% (see Section 4). Amphipathicity, and the distribution of polar and hydrophobic residues (often described by hydrophobic moment and polar angle), are then responsible for interactions between the peptide and the membrane after the peptide adheres to the membrane surface.^114,115^ As is the case with the net charge, the hydrophobicity of an AMP must strike a balance. Increasing the hydrophobicity of an AMP has been shown to improve its antimicrobial activity.^116^ However, given that both bacterial and mammalian membranes share a hydrophobic core, increased AMP hydrophobicity also results in decreased selectivity for bacterial cells, often leading to haemolysis. A high hydrophobic content can also result in reduced antimicrobial activity due to the formation of AMP aggregates.^116^

While the initial interaction between an AMP and the bacterial membrane often relies on the charge of the AMP, some AMPs are known to bind to a particular component of the membrane. For example, both nisin and mutacin bind to lipid II, a precursor of peptidoglycan, while daptomycin interacts with membrane PG.^117–119^ Whether it is due to charge or an interaction with a particular membrane component, the peptides are brought from solution to the surface of bacteria. α-Helical AMPs are typically unstructured in solution and only adopt their secondary structure upon interaction with the membrane. In contrast, AMPs with β-sheet structures are often already folded in solution as they typically have stabilising disulfide bridges.^114^ As the peptide-to-lipid ratio on the cell surface increases, the AMPs can bring about disruptive structural changes in the membrane through interaction with phospholipid heads on the membrane surface. When a threshold concentration is reached, insertion of the peptides into the hydrophobic parts of the membrane typically occurs.^120^

**Fig. 5:**
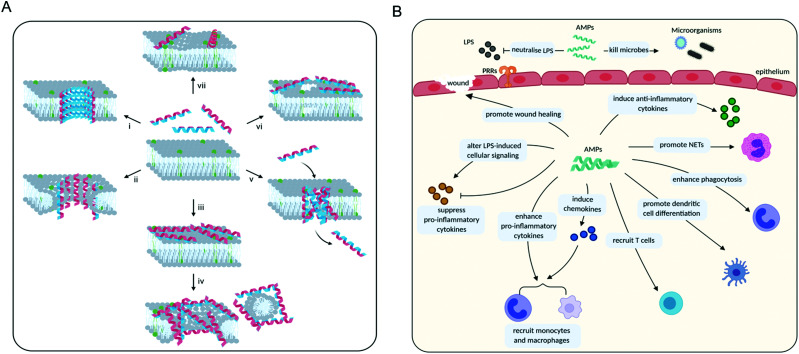
(A) Different membrane disruption mechanisms displayed by AMPs. The membrane is shown in pale grey with negatively charged phospholipids highlighted in green. The amphipathic nature of peptides is demonstrated by the double colouring of the helices: blue represents the surface with positively charged residues and red the surface with hydrophobic ones. (i) In the barrel-stave model peptides form a pore spanning the membrane. (ii) In the toroidal model peptides cause local membrane curvature resulting in membrane disruption. (iii) Peptides may cover the surface of the membrane (carpet model). As the peptide to lipid ratio increases, this can lead to membrane dispersal (detergent model, iv). (v) Peptides may form leaky aggregates in the membrane (aggregate model). (vi) In the electroporation model, peptide accumulation on the membrane surface causes an electric potential build-up resulting in a membrane disruption. (vii) Phospholipid clustering can change the morphology of the membrane. (B) Roles of AMPs in modulating the immune system. NETs: neutrophil extracellular traps, LPS: lipopolysaccharide, PRRs: pattern recognition receptors.

Once an AMP has bound to the membrane, there are three major mechanisms by which it may exert its membrane-disruptive activity: barrel-stave, toroidal or carpet (Fig. 5). In the “barrel-stave” model, when the threshold concentration of the (often α-helical) peptide is reached, a bundle forms and inserts into the membrane as a pore (Fig. 5A, i). The peptides, acting as “staves”, are oriented so that polar residues face the inside of the pore, and the hydrophobic residues the outside, while maintaining lateral interactions between individual peptides.^121,122^ The “toroidal” model is similar to the ‘barrel-stave’ model in that a pore is formed, but instead of spanning across the membrane the peptides induce local thinning and curvature in the membrane resulting in membrane disruption (Fig. 5A, ii).^121,123^ Finally, in the “carpet” model, the peptides cover the membrane surface (Fig. 5A, iii), disrupting its integrity and ultimately causing the membrane to disperse into particles that are sequestered by the AMPs.^124^ This later phase is sometimes also referred to as the ‘detergent’ model (Fig. 5A, iv).

In addition, there are a number of less frequently encountered mechanisms of action related to membrane disruption. For some AMPs it has been suggested that when a threshold concentration is reached, the peptides insert into the membrane as aggregates (Fig. 5A, v). These aggregates are leaky and can serve as a transient structure to facilitate the translocation of the peptide across the membrane to reach an intracellular target.^115^ There are also studies showing that some AMPs do not permeabilise the membrane and only cross it to reach their primary intracellular target, such as buforin II which binds to DNA.^120^ However, at high concentrations most membrane-binding peptides are expected to cause membrane leakage, including buforin II.^122,125^

An ‘electroporation’ model has also been described in which the charge of the peptides accumulated on the outer surface creates a sufficiently high electric potential difference that pore formation occurs (Fig. 5A, vi). Unlike other models, these pores are not lined with the peptides.^126^ Some AMPs have also been shown to alter the distribution of the phospholipids in the membrane, which on its own can bring about the desired effects (Fig. 5A, vii). Clustering of certain phospholipids can: change the local curvature of the membrane; cause a phase separation; make those phospholipids unavailable for other interactions; or alter the membrane thickness.^61,127^ This list is not exhaustive and other mechanisms are possible, though rare.^123^ Furthermore, it is likely that in many cases AMPs actually employ several mechanisms of action simultaneously.^114^

Various methods have been used to study the mechanisms of action of AMPs, both with live cells and artificial membranes or vesicles. Microscopy with labelled peptides has played a key role in identifying their location and effect on membranes. In addition, the folding of AMPs can be monitored by circular dichroism spectroscopy; the interaction of the peptide aa with lipids can be identified by NMR; and lipid ordering and mass of membranes can be measured by dual polarisation interferometry.^120,128^ Other methods of investigating AMP mechanisms of action include, but are not limited to: measurement of a voltage across the membrane; X-ray crystallography and diffraction; neutron diffraction; and membrane vesicle permeabilisation. Finally, computational methods, such as MD or Monte Carlo simulations, are heavily employed.^118,129,130^

The means by which membrane disruption results in cell death can vary. Membrane disruption ultimately results in dissipation of vital chemical gradients, such as that of protons or metal ions.^120^ The change in ion gradients can result in an influx of water into the cell due to osmotic pressure resulting in swelling and eventually bursting.^114^ Non membrane-disrupting peptides usually interfere with essential intracellular pathways.^120^ AMPs may also act synergistically with other antibiotics by either reducing the barrier to cell entry, thereby facilitating the antibiotic reaching its intracellular target (*e.g.* a combination of colistin with rifampicin), or by acting on an additional target (*e.g.* a combination of daptomycin with ampicillin).^131^

One appealing element of the tendency for AMPs to cause membrane disruption is that they may be better suited than conventional antibiotics to kill not only metabolically active bacterial cells, but also slow growing and dormant persister cells that are present in significant numbers in biofilm architectures (see Section 2.2).

### 6.2 Immunomodulatory activity

In addition to the direct killing of microorganisms through membrane disruption or inhibition of vital intracellular processes, many AMPs are also able to interact with the host immune system to modulate its inflammatory responses. In order to respond to an infection, the body recognises molecules that are not associated with human cells. These molecular markers are referred to as pathogen-associated molecular patterns (PAMPs) and danger-associated molecular patterns (DAMPs). The first category includes molecules derived from microorganisms and the second is associated with damaged or stressed human cells. PAMPs and DAMPs are typically detected by receptors known as pattern-recognition receptors (PRRs) from various cells of the innate immune system. Upon detection of PAMPs/DAMPs by PRRs, various signalling pathways are activated leading to an inflammatory response. During inflammation, blood vessel dilation and increased blood flow to the site of infection/damage enable phagocytic white blood cells (neutrophils, macrophages *etc.*) and components of the complement system to swarm the area and combat invading pathogens and/or begin the process of repair.^132^ The response is usually immediate, nonspecific, and tightly controlled. Intracellular signalling during an inflammatory response is highly complex and involves multiple signalling pathways that ultimately lead to activation of the adaptive immune response.^132–134^ Once the infection has been resolved, inflammation decreases and the number of circulating white blood cells returns to basal level.^134^

In some instances, the body induces an unusually severe inflammatory response to an infection, which can lead to systemic inflammatory conditions such as sepsis. Infection leading to sepsis remains a serious problem in hospitals and is currently one of the biggest health problems world-wide.^134^ In severe cases, sepsis causes a dangerous drop in blood pressure, known as “septic shock”, which can quickly result in multiple organ failure and death. Mortality rates from septic shock are close to 30–50% in a hospital setting.^135^ Sepsis is often caused by lipopolysaccharide (LPS), also known as endotoxin, from Gram-negative bacteria, and lipoproteins (LPs) from Gram-positive bacteria. The bacteria that are most frequently isolated from patients with sepsis include *S. aureus*, *S. pneumoniae*, *E. coli*, and *P. aeruginosa*.^134,136^ One of the main challenges in fighting sepsis is developing a drug that can efficiently kill bacteria without releasing the inflammation-inducing toxins LPS and LP.^137^ Indeed, studies have shown that treatment with conventional antibiotics can worsen the release of endotoxins from the bacteria into the blood system.^138,139^ For a discussion of the bacterial toxins involved in sepsis, we refer the reader to the review by Girish Ramachandran.^140^

AMPs are a key component of the innate immune system in multicellular organisms with the ability to elicit anti-inflammatory and immunostimulatory effects. For example, considerable effort has shown that several natural AMPs are able to neutralise LPS-induced inflammation in both *in vitro* and *in vivo* models of sepsis.^137^ AMPs are also able to directly recruit antigen-presenting cells (*e.g.* monocytes and macrophages) to the site of infection or indirectly *via* the induction of chemokines. Furthermore, they can suppress the expression of pro-inflammatory cytokines; enhance phagocytosis and pro-inflammatory responses to nucleic acids; induce the expression of anti-inflammatory cytokines; influence the differentiation of dendritic cells and the polarisation of T cells; and promote wound healing (Fig. 5B).^133^ Defensins and cathelicidins are currently the most extensively explored mammalian AMPs and their immunomodulatory activity has been recently summarised by Davidson and co-workers.^133^

The family of cyclic lipopeptide polymyxins are well known for their ability to modulate inflammatory cytokines by directly binding LPS, thereby neutralising its ability to activate PRRs.^141^ The positively charged 2,4-diaminobutyric acid (Dab) residues of polymyxins are able to interact with the negatively charged phosphate group of the lipid A component of LPS.^142^ However, being cationic is not a prerequisite for effective LPS binding as the cecropin D-like peptide (Gm1), an analogue of the negatively charged AMP cecropin, is also able to bind LPS. Gm1 shows promise as a template for further studies in peptide-based drug development of antisepsis compounds.^143–145^

Polymyxins are last-resort antibiotics due to their significant nephro- and neurotoxicity. To overcome this toxicity, Perego and co-workers have developed a blood endotoxin removal cartridge in which polymyxin B (PMB) is adsorbed onto polystyrene fibres to form an extracorporeal direct hemoperfusion device known as Toraymyxin™. Briefly, this device is bound to a machine that draws the blood out of the patient's vein, which then flows through the cartridge and is purified by adsorption of the endotoxins to the immobilised PMB. The machine subsequently delivers the purified blood back to the patient.^146,147^ This therapy has been used for the treatment of sepsis in Japan and Europe and has significantly reduced mortality in patients with severe sepsis and septic shock.^147–149^ Further details on polymyxins and their structure are provided in Section 8.

One of the most challenging aspects of developing immunomodulatory peptides as therapeutics is deciphering the interaction between the peptides and the immune system. Up to now, many studies have focused on identifying natural peptides and their variants as antibacterial agents, as well as anti-endotoxin drugs, the results of which have been summarised by Lohner and co-workers, and Kidric and co-workers.^137,150^ These reviews describe many peptides and proteins with LPS-binding properties that are relevant for the research of AMPs, such as cationic antimicrobial proteins from human and rabbit neutrophil leukocytes (CAP37 and CAP18); lipopolysaccharide-binding protein (LBP); bactericidal-permeability increasing protein (BPI); Limulus anti-LPS factor from the Atlantic horseshoe crab, *Limulus polyphemus* (LALF); and lactoferrin (LF), the mammalian iron-binding glycoprotein.^137,150^

Many experimental drug therapies to treat sepsis have failed clinical trials and critical care physicians are currently lacking drugs specifically approved for the treatment of sepsis. Although talactoferrin α, a recombinant form of the human lactoferrin protein, has failed clinical trials, antimicrobial peptides and proteins show a lot of promise in this area because they confer bactericidal and/or immunomodulatory effects, which may also pave the way for combination therapies of different AMPs, with anti-inflammatory or bactericidal effects.^134,151–153^ While initial studies have been performed with the classic natural AMP families, we believe an area of growth for AMP research is to fully elucidate their immunomodulatory activity such that they may be successfully employed for the treatment of sepsis.

## 7 Bacterial resistance to AMPs

While AMR has already been discussed (see Section 2), here we will highlight bacterial resistance mechanisms of particular relevance to AMPs, a topic which has also been reviewed elsewhere.^154,155^ These mechanisms include (but are not limited to): surface remodelling, the production of AMP-sequestering proteins, capsule synthesis, biofilm formation, the expression of efflux pumps and the co-opting of AMP function.

### 7.1 Surface remodelling

The majority of AMPs function by binding to and compromising bacterial membranes. As such, a key resistance mechanism for bacteria is to chemically and structurally alter the permeability and fluidity of their membranes to prevent interaction with AMPs. Bacterial strains displaying resistance towards polymyxins, cathelicidins, and defensins have all been shown to have altered membrane lipid compositions and reduced levels of particular membrane proteins and ions.^155^ This process of membrane alteration is referred to as surface remodelling.

**Fig. 6:**
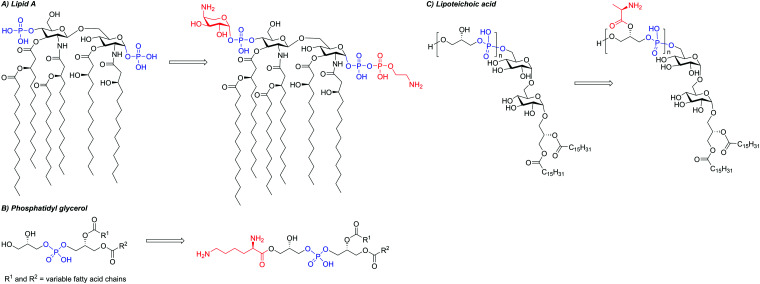
Various modifications that can occur to bacterial membrane and cell wall components that confer resistance towards AMPs: (A) modification of lipid A with phosphoethanolamine or 4-amino-4-deoxy-l-arabinose. (B) Modification of lipids with cationic residues (*e.g.* Lys). (C) d-Alanine esterification of teichoic acid residues. Negatively charged groups highlighted in blue, positively charged groups highlighted in red.

For cationic AMPs, interaction with the target membrane is driven by electrostatic attraction to the negatively charged bacterial surface. To prevent this attraction, some Gram-negative bacteria acquire chromosomally-encoded resistance genes that reduce the overall negative charge of their outer membrane.^110,156^ For example, polymyxin B-resistant *V. cholerae*, *P. aeruginosa*, *S. enterica*, and *A. baumannii* have been shown to modify lipid A, part of the key Gram-negative OM component LPS, with phosphoethanolamine or 4-amino-4-deoxy-l-arabinose residues, reducing its negative charge (Fig. 6A).^157,158^ These modifications confer resistance to many cationic AMPs, including polymyxin B, and occur primarily due to regulatory complexes such as PmrAB and PhoPQ, which alter gene expression in response to environmental changes.^156,159–161^ For an in-depth discussion of LPS modifications we direct the reader towards an excellent review by Hankins and co-workers.^162^ In the case of polymyxin B, it has also been found that resistance can be plasmid-mediated in Gram-negative bacteria.^156^ Indeed, polymyxin resistance was reported in 2016 in *E. coli* SHP45 as a result of the plasmid-mediated mobilised colistin resistance-1 (*mcr-1*) gene, which adds phosphoethanolamine to lipid A (Fig. 6A).^157,163^ Since 2016, the gene has been detected in several bacterial species and new *mcr* variants have been identified.^164–174^ Membrane alterations have also been observed in Gram-positive strains, for example, modification of the membrane lipid PG of *S. aureus* with a Lys residue, which is proposed to reduce the overall negative charge (Fig. 6B).

Some bacterial species evade AMP activity by modifying their cell walls in addition to, or instead of, their membranes. For example, cell wall thickening has been observed in Gram-positive *S. aureus* and Gram-negative *E. coli*.^175^ The cell wall of Gram-positive bacteria is negatively charged due to the large quantity of teichoic acid present, which is covalently linked to peptidoglycan.^176^ A variety of modifications can be made to teichoic acid, including esterification of the d-alanine residues (Fig. 6C).^177^ In this instance, it has been suggested that the primary function of these d-alanine substitutions is to increase the cell-wall density, rather than to reduce overall net charge.^178^

### 7.2 AMP-Degrading and sequestering proteins

Many pathogenic bacteria produce cytosolic and/or extracellular proteases, which hydrolyse and inactivate AMPs. This is often an inherent resistance mechanism and there are several studies linking the level of bacterial protease expression to the level of susceptibility to AMPs.^155^ Linear AMPs are often more prone to hydrolysis than macrocyclic AMPs due to the relative accessibility of the peptide backbone in the linear conformation.^179,180^

As an alternative to degradation strategies, some bacteria produce non-proteolytic AMP-sequestering proteins, which bind to AMPs and render them inactive, for example, staphylokinase produced by *S. aureus*.^181^*P. aeruginosa* has been observed to secrete a virulence factor, LasA, which causes shedding of a cell-surface heparan sulfate proteoglycan, syndecan-1.^182^ It is believed that syndecan-1 can neutralise cationic AMPs such as cathelicidins.

### 7.3 Capsule synthesis

Some pathogenic bacteria, including *P. aeruginosa*, can produce an anionic polysaccharide capsule that surrounds the cell.^183^ The capsule is thought to limit access of AMPs to the outer membrane, firstly by providing a physical barrier between the two.^184^ Secondly, capsule polysaccharides can bind to and sequester AMPs *via* electrostatic interactions.^184^ It has been shown that AMPs can modulate capsule expression in *K. pneumoniae*: the presence of polymyxin B and lactoferrin correlated with a higher quantity of capsule polysaccharide bound to the cell surface.^185^

### 7.4 Biofilm formation

Biofilms are a key mechanism by which bacteria can evade antibiotic activity. A biofilm consists of bacterial communities attached to a surface, and embedded in an extracellular matrix composed of lipids, DNA, polysaccharides, and proteins, which can have a large impact on cell-to-cell interactions and bacterial virulence.^12^ Biofilms are able to impart AMP resistance, likely due to the inability of the AMP to penetrate the biofilm matrix, in a similar manner to a capsule.^13^ Polysaccharides in the biofilm matrix are thought to bind to and sequester penetrating AMPs.^186^ A more putative mechanism of biofilm-induced resistance involves the presence of persister cells with a specific MDR phenotype within the biofilm.^187,188^

The response of bacterial biofilms to AMPs depends on the concentration, structure, and composition of the AMP.^183^ For example, sub-inhibitory concentrations of both polymyxin B and E (colistin) can induce biofilm formation, whereas some cationic AMPs have been reported to prevent biofilm formation or disrupt pre-formed biofilms.^189–191^

### 7.5 Membrane transport systems/efflux pumps

Even if an AMP can evade these resistance mechanisms and reach the bacterial membrane or cytoplasm, it can then be removed from the cell by membrane transport systems (or efflux pumps). Some efflux pumps are able to export multiple AMPs, for example, the energy-dependent MtrC–MtrD–MtrE efflux pump found in *Neisseria gonorrhoeae* and *meningitidis* is known to efflux the AMPs PG-1, PC-8, LL-37, and TP-1.^192^ Conversely, in *S. aureus* the *qacA* gene encodes for a proton motive force-dependent efflux pump which seems to only confer resistance towards one peptide, tPMP-1; however the exact nature of this resistance is yet to be fully elucidated.^193^ In addition to expressing efflux pumps that can export non-endogenous AMPs, bacteria typically express efflux pumps capable of exporting AMPs produced intracellularly as part of their own defence strategy, in order to avoid their harmful effects.^194^

In a different strategy, Mason and co-workers observed that nontypeable *Haemophilus influenzae* actively imports AMPs such as cathelicidins and defensins *via* the multifunctional Sap import system.^195^ Once in the cytosol, the AMPs are degraded by cytosolic proteases. The authors hypothesised that the Sap import system reduces the AMP concentration at the membrane and periplasmic space, thereby preventing membrane disruption.

### 7.6 Co-opting AMP function

A particularly unusual mechanism of AMP resistance is displayed by *Shigella flexneri*, which exploits the properties of cationic AMPs and uses them to invade host cells.^196^*S. flexneri* infect the host's epithelial cells, and adhesion between the bacterial and mammalian cells is necessary for successful invasion. As the cell surfaces of both the host epithelial cells and the bacteria contain anionic components, the bacteria employ cationic AMPs released by the host to facilitate adhesion. This example of bacteria not just evading AMP activity but repurposing them to promote virulence highlights how resourceful and adaptive these organisms can be.

The field of AMPs is relatively young and so investigations into the mechanisms of bacterial resistance towards these antimicrobial agents are largely preliminary or inconclusive. Further studies must be performed, particularly on pathogens of high relevance to human health, so that these resistance mechanisms may be understood and overcome. Often, bacterial resistance to AMPs is overlooked in discussions of AMPs as promising antimicrobial therapeutics, likely due to their underrepresentation in the clinic. However, as therapeutic AMPs are developed, it is vital that more is understood about resistance mechanisms so that the mistakes that have led to the current AMR crisis can be avoided. Beyond ensuring appropriate prescribing and usage of AMPs, chemical synthesis and bioengineering will play important roles in altering these compounds to reduce their susceptibility to the mechanisms of resistance outlined here.

## 8 (Non)ribosomally-synthesised AMPs and the current clinical landscape

Nature has elegantly evolved several cellular machineries capable of producing peptides and proteins with precise control over their sequence, length, stereochemistry and topology. Many AMPs belong to the large family of gene-encoded ribosomally-synthesised peptides (RPs), which are produced by nearly all forms of life including animals, bacteria, fungi, plants, and insects.^121^ For an in-depth discussion of ribosomally-synthesised AMPs, we refer the reader to a review from Abraham and co-workers, who considered these AMPs in five classes, such as cationic peptides enriched in a particular aa (*e.g.* proline, arginine, tryptophan, phenylalanine, or glycine); linear cationic α-helical peptides without cysteine residues; anionic peptides rich in glutamic acid and aspartic acid; anionic and cationic peptides containing cysteine residues; and neutral peptides.^197^ All ribosomally-synthesised AMPs share common features. Often, they are derived from relatively short precursor peptide sequences and are translated as inactive pro-peptides, requiring at least one proteolytic step for their activity (*e.g.* cathelicidins and defensins). Different proteases can generate various peptide lengths and enable access to diverse antimicrobial and immunomodulatory properties. As such, the presence of appropriate proteases, as well as their expression levels, are important factors for regulating the function of the AMPs.^198–202^ In bacteria, the genes encoding pro-peptides are often clustered with genes that encode proteins involved in modifying the pro-peptides, as well as those that impart the host with resistance against the generated AMP, and those that are responsible for its secretion.^203^

Once translation has occurred at the ribosome, some peptides undergo further PTMs, sometimes referred to as “post-ribosomal peptide synthesis” (PRPS). These ribosomally-synthesised and post-translationally modified peptides, or RiPPs, are becoming increasingly recognised as an untapped source of antimicrobial drugs.^204–206^ They contain a “leader peptide” that is appended to the N-terminus of the precursor peptide (in rare cases to the C-terminus, in which case it is known as the “follower peptide”). The leader peptide is known to play many roles including the recruitment of the PTM enzymes, identification of the proteolysis sites, and peptide export.^207,208^ Typical PTMs can include (but are not limited to): the formation of complex knotted topologies (*e.g.* lasso peptides, cyclotides); the formation of thioether bridges (*e.g.* lanthipeptides, sactipeptides); and the formation of backbone heterocyclic moieties (*e.g.* linear azole peptides, thiopeptides). These modifications provide access to regions of chemical space that are not explored by RPs, as well as imparting high stability to both chemical and metabolic degradation, making RiPPs attractive peptide drugs.

Despite the appeal of ribosomally-synthesised AMPs, to the best of our knowledge none have been brought to market for therapeutic applications, although some are currently undergoing clinical trials. Nevertheless, a few AMPs have been successfully used in other applications such as in agriculture and the food industry. For example, the prototypical lanthipeptide, nisin, has been used as a preservative in food for over 40 years.^131,204^

In contrast to RPs and RiPPs, there is a very clinically significant subset of nonribosomally-synthesised peptides (NRPs), which are synthesised without the need for ribosomes and messenger RNAs. This nonribosomal synthesis is achieved by multienzyme machineries, known as nonribosomal peptide synthetases, which are able to incorporate non-proteinogenic aa, such as d-aa, as well being able to perform modifications such as cyclisation, glycosylation, hydroxylation, and acylation.^209,210^ To date, NRPs have been predominantly used as systemic and topical antibacterials, followed by antitumour agents, antifungals and animal feed additives, and represent more than 20 marketed drugs.^209^ It is noteworthy that many AMPs, including RiPPs and NRPs, are not limited to acting as anti-infectious agents. They display a wide array of biological activites, such as inhibition of nucleotide/protein synthesis, metal ion chelation, cell membrane permeation, cell apoptosis regulation, cytokine release modulation, or acting as siderophores.^207,210–214^

**Fig. 7:**
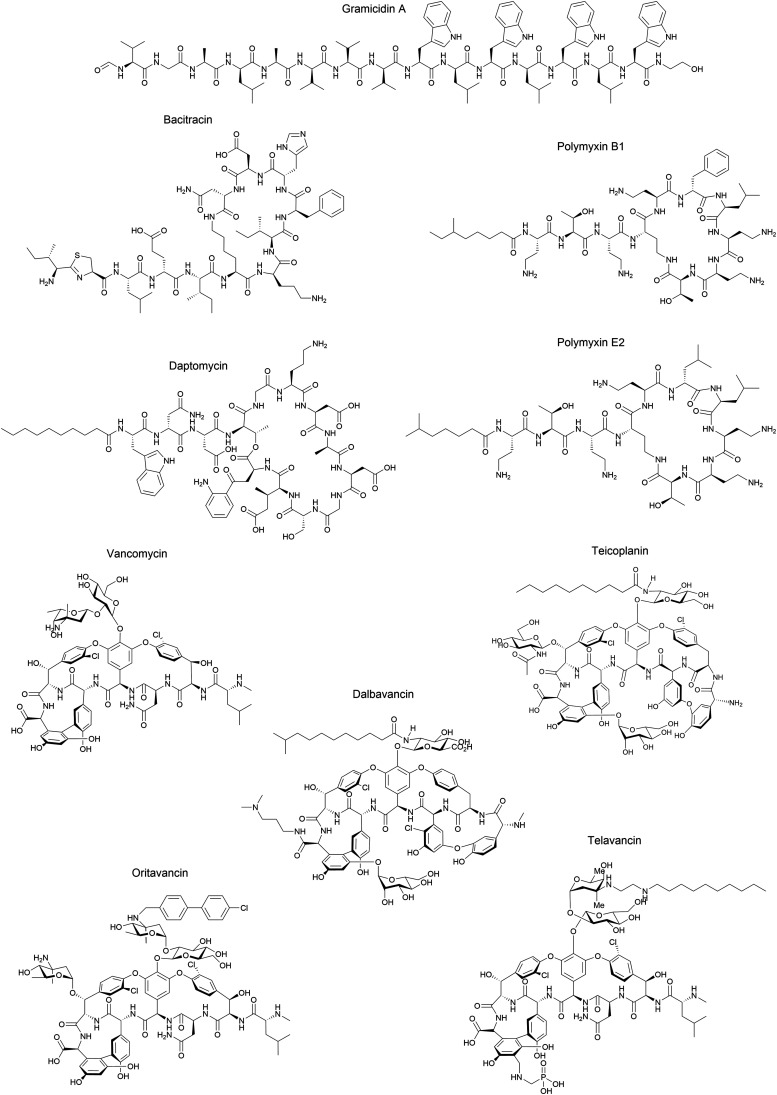
Structural formulae of some marketed peptide-based antibiotics. For gramicidin D, the polymyxins, and teicoplanin, only the most abundant components of the clinically used mixtures are shown.

Although some of these NRPs are key players in the clinic (*e.g.* vancomycin), there are still very few AMPs that are currently undergoing preclinical studies or clinical trials, approved by the FDA, or undergoing commercial development.^197,215^Fig. 7 and Table 3 show a non-exhaustive list of marketed peptide-based antibiotics: gramicidin D, polymyxin B/colistin (polymyxin E), bacitracin, daptomycin, vancomycin, oritavancin, dalbavancin, telavancin, and teicoplanin.^216,217^

**Table 3:** Marketed antimicrobial peptides

Active agent	Structure	Spectrum of activity	Mechanism of action	Source	Marketed
Gramicidin D (mixture of gramicidin A, B, and C)	Linear peptide	Antibacterial peptide	Forms ion-channels to increase membrane permeability	*Bacillus brevis*	1952
Bacitracin	Cyclic peptide	Antibacterial against Gram-positive bacteria	Prevents the transfer of mucopeptides into the cell wall, resulting in the inhibition of cell wall formation	*Bacillus subtilis* and *Bacillus licheniformis*	1948
Colistin (polymyxin E)	Cyclic lipopeptide	Antibacterial	Binds to LPS at the outer membrane and interacts with inner membrane	*Paenibacillus polymyxa* var. *colistinus*	1958
Polymyxin B	Cyclic lipopeptide	Antibacterial	Binds to LPS at the outer membrane and interact with inner membrane	*Bacillus polymyxa*	1952
Vancomycin	Tricyclic glycopeptide containing vancosamine and glucose	Antibacterial against Gram-positive bacteria	Inhibits synthesis of the bacterial cell wall (peptidoglycan)	*Amycolatopsis orientalis*	1955
Teicoplanin	Lipoglycopeptide	Antibacterial against Gram-positive bacteria	Inhibits synthesis of the bacterial cell wall (peptidoglycan)	*Actinoplanes teichomyceticus*	1988
Dalbavancin	Semi-synthetic glycopeptide	Antibacterial against Gram-positive bacteria	Inhibits synthesis of the bacterial cell wall (peptidoglycan)	Semi-synthetic teicoplanin derivative	2014
Daptomycin (LY146032)	Cylic lipopeptide	Antibacterial	Disrupts the bacterial membrane	*Streptomyces roseosporus*	2003
Oritavancin (LY333328)	Lipoglycopeptide	Antibacterial against Gram-positive bacteria	Disrupts the bacterial membrane and inhibits synthesis of the bacterial cell wall (peptidoglycan)	*Amycolatopsis orientalis*	2014
Telavancin (semi-synthetic derivative of vancomycin)	Glycopeptide	Antibacterial against Gram-positive bacteria	Disrupts the bacterial membrane and inhibits synthesis of the bacterial cell wall (peptidoglycan)	*Amycolatopsis orientalis*	2009

Gramicidin D was initially isolated by Dubos in 1939 from the soil bacterium *Bacillus brevis* and was identified as a mixture of three different analogues (gramicidin A, B, and C) that differed by one aa. The gramicidins are 15-mer linear peptides, which are synthesised non-ribosomally by multienzyme complexes and consist of a mixture of l- and d-aa. Gramicidin A (GA) is the major component of gramicidin D, while gramicidin B and C comprise 6% and 14% of the mixture, respectively. The l-Trp residue at position 11 in gramicidin A is replaced by l-Phe in gramicidin B and l-Tyr in gramicidin C, and each analogue is individually active against pathogenic bacteria.^218–221^ When injected intravenously, gramicidin D exhibits toxicity in mice and therefore cannot be used to treat systemic infections. However, it is highly effective *via* topical application and was used during World War II to treat wounds and ulcers, becoming the first AMP to be commercially manufactured.^222^ Gramicidin D was granted FDA-approval in 1955 as a component of Neosporin®, a triple antibiotic ointment for the treatment of bacterial conjunctivitis.^223^

Polymyxin (PM) is the generic name for a group of antibiotics discovered in *Bacillus polymyxa* in 1947, among which colistin is a member. PMs are nonribosomally synthesised cyclic lipopeptides, which contain five Dab residues (a homologue of Lys) giving PMs a net positive charge of +5 at physiological pH. Additionally, the hydrophobic residues and N-terminal fatty acid chain make PMs amphipathic. Generally, PMs differ in the nature of the fatty acid chain, the hydrophobic d-aa residue at position 6, and the l-aa at position 7. Each component of polymyxin B and E (colistin) has been summarised by Hoogmartens and co-workers.^224^ Essentially, PMB and PME differ by a single aa in the cyclic moiety, with a phenylalanine in PMB and a leucine in PME at position 6. To date, PMB and PME are used in the clinic to treat systemic infections only as a last resort antibiotic due to their associated nephro- and neurotoxicity.^45^ In addition to their bactericidal activity, PMB and PME are well known for their LPS-binding properties, and are being considered for their potential to treat septic shock (see Section 6.2).^225,226^ Despite their structural similarity, PMB and PME are administered in very different formulations. PMB is injected intravenously as a sulfate salt, whereas colistin is injected as a prodrug in the form of colistin methanesulfate (CMS).^227^ PMB and PME show potent antimicrobial activity and exquisite selectivity against Gram-negative bacteria, such as *P. aeruginosa*, *K. pneumoniae*, and *A. baumannii*. Colistin was approved by the FDA in 1962 and manufactured by Endo Pharmaceuticals, Pennsylvania, USA as colistin sulfate (Coly-Mycins).^216^

Bacitracin is a nonribosomally-synthesised cyclic peptide antibiotic that was isolated from *Bacillus subtilis* and *Bacillus licheniformis* in 1945. This cyclic peptide has been shown to display a narrow bactericidal activity spectrum, primarily against Gram-positive cocci and bacilli, including *Staphylococcus*, *Streptococcus*, and *Clostridium difficile*, as well as some Archaebacteria.^228^ Bacitracin was approved by the FDA in 1948 for the short-term prevention and treatment of acute and chronic localised skin infections, and is typically used as a single therapy or as part of a tritherapy ointment alongside neomycin and polymyxin B. Although less common, this peptide can also be injected intramuscularly for systemic treatment of infantile streptococcal pneumonia and empyema.^229^

Daptomycin was isolated in the early 1980s as a fermentation product from *Streptomyces roseosporus*. It is a nonribosomally-synthesised 13-mer cyclic lipopeptide bearing a decanoyl side chain that exhibits rapid bactericidal activity against Gram-positive bacteria, including methicillin-resistant *Staphylococcus aureus* (MRSA).^230,231^ Daptomycin and its derivative cubicin, initially manufactured by Cubist Pharmaceuticals and now by Merck & Co., were approved by the FDA in 2003 for the treatment and prevention of infectious diseases. Cubicin and Cubicin RF (a new formulation), can be injected to treat complicated skin and skin structure infections (cSSSI), as well as *S. aureus* bloodstream infections.^216,232,233^

Vancomycin is a nonribosomally-synthesised tricyclic glycopeptide isolated in 1957 from the fungus *Amycolatopsis orientalis*. It consists of a 7-mer tricyclic peptide structure attached to a vancosamine–glucose disaccharide. This antibiotic represents one of the earliest discoveries in this field, and it has been in clinical use for almost 60 years. Vancomycin is most effective against Gram-positive cocci and bacilli where it inhibits cell wall formation. It is used as a first-line agent for MRSA infections, including bacteraemia, endocarditis, pneumonia, cellulitis, and osteomyelitis. This antibiotic has also been used to treat serious Gram-positive infections for patients who are allergic to penicillins and cephalosporins.^44,216,234^ Telavancin is an FDA-approved derivative of vancomycin and was brought to market in 2009, with vancomycin having itself been FDA-approved as an oral solution in 1983. Oritavancin and dabavancin are also glycopeptides that were both FDA-approved in 2014. All three of these lipoglycopeptides exhibit more potent antibacterial activity than vancomycin and are effective against vancomycin-resistant bacteria. They are currently used against cSSSI caused by *S. aureus*.^235^

Teicoplanin is a lipoglycopeptide isolated from *Actinoplanes teichomyceticus* in 1978 and was marketed in Europe in 1988 and in Japan in 1998. However, despite its widespread use, it has never been approved in the US.^236^ Similarly to vancomycin, teicoplanin is a 7-mer tricyclic glycopeptide, but bearing a fatty acid tail. The clinically used teicoplanin consists of a mixture of five lipoglycopeptides that differ in the length and branching of the fatty acid tail. This drug has an activity spectrum similar to vancomycin primarily against Gram-positive bacteria.^237^

In addition to the marketed AMPs, Table 4 shows a non-exhaustive list of recently designed AMPs that have proceeded to clinical trials. A complete list of clinical data on AMPs can be found on the Data Repository of AMPs (DRAMPs).^238^ Up to now, more than 3000 clinically relevant AMPs have been designed and identified, but only a few have successfully proceeded to either preclinical studies, clinical trials, FDA approval, or market entry. Several AMPs, such as pexiganan (MSI-78), iseganan (IB-367), omiganan (MBI-226, analogues of indolicidin) and neuprex (rBPI21), have failed the trials for reasons such as flawed trial designs, ineffectiveness of the drugs during trials, unimproved antimicrobial activity compared to conventional antibiotics, or as in the case of Iseganan, increased mortality compared to placebo.^215,239^ Nevertheless, trial failure can be caused by non-pharmacological reasons, such as instability of the formulated peptides, confounding biological activities of the peptides (*e.g.* pro- and anti-inflammatory effects, see Section 6.2), and high manufacturing costs.^240^ Also, despite the broad-spectrum antimicrobial activity of membrane-disruptive AMPs *in vitro*, they have frequently resulted in systemic and local toxicity *in vivo*, which hampers the successful transition from bench to bedside.^215^

**Table 4:** Non-exhaustive list of AMPs as anti-infective therapeutics in clinical trials^215^

AMPs	Administration	Mechanism of action	Clinical application	Development stage (decision)	Company
Human lactoferrin-derived peptide hLF1-11	Intravenous	Binds DNA	Diseases mediated by LPS and infections caused by fungi	Phase 1 (completed)	AM-Pharma
Histatin-1 and -3, P-113 (histatin derivatives)	Topical, mouthwash	Generates reactive oxygen species	Chronic infections caused by *P. aeruginosa*, gingivitis, and periodontal diseases	Phase 1, Phase 2–3	Demgen
LTX-109 (lytixar)	Topical	Permeabilises bacterial membrane	Uncomplicated skin infections caused by Gram-positive bacteria, also methicillin-resistant and -sensitive *S. aureus* nasal carriage	Phase 2	Lytix Biopharma
Opebacan (rBPI21, neuprex)	Intravenous	Permeabilises bacterial membrane	Burn, wound, and meningococcal infections	Phase 2 (completed)	Xoma
Opebacan (rBPI21, neuprex)	Intravenous	Permeabilises bacterial membrane	Post-traumatic infections	Phase 2 (failed)	Xoma
Omiganan (MBI-226, MX-594AN)	Topical	Permeabilises bacterial membrane	Infections associated with catheter	Phase 3 (completed)	Migenix
Omiganan (MBI-226, MX-594AN)	Topical	Anti-inflammatory	Rosacea, severe acne, vulval epithelial neoplasia, and genital warts	Phase 2b (completed)	Migenix, Biofrontera
EA-230	Topical	Immunomodulation	Sepsis, endotoxemia	Phase 2 (recruiting)	Exponential Biotherapies
IMX942 (dusquetide)	Topical	Immunomodulation	Oral mucositis	Phase 3 (recruiting)	Inimex Pharmaceuticals
PMX-30063 (brilacidin)	Topical or intravenous	Permeabilises bacterial membrane	Acute bacterial skin infections	Phase 2	Innovation Pharmaceuticals
OP-145	Topical (ear drops)	Neutralises bacterial toxins	Chronic otitis media	Phase 2 (completed)	OctoPlus
XF-73	Topical	Permabilises bacterial membrane	Infections caused by staphylococcus during surgeries, nasal carriage	Phase 2 (recruiting)	Destiny Pharma
XOMA-629	Topical (gel)	Permeabilises bacterial membrane	Impetigo	Phase 2a	Xoma
DPK 060	Topical	NA^a^	Infections caused by eczematous lesions	Phase 2 (completed)	DermaGen AB
Murepavadin (POL7080)	Intravenous	Targets the outer membrane LPS transport protein D	Lower respiratory infections caused by *P. aeruginosa* and ventilator-associated pneumonia	Phase 3 (recruiting)	Polyphor
Surotomycin	Oral	Depolarises bacterial membrane	Diarrhoea caused by *C. difficile*	Phase 3 (completed)	Cubist Pharmaceuticals, Merck & Co.
Iseganan (IB-367)	Aerosol, mouth wash	Permeabilises bacterial membrane	Ventilator-associated pneumonia, oral mucositis	Phase 3 (failed)	Intrabiotics Pharmaceuticals
XMP 629	Topical	NA^a^	Acne	Phase 3 (failed)	Xoma
Talactoferrin (TLF, rhLF)	Oral	NA^a^	Sepsis	Phase 3 (suspended)	Agennix
Pexiganan (locilex)	Topical or intravenous	Permeabilise bacterial membrane, stimulates defensin production	Infections caused by diabetic foot ulcer	Phase 3 (failed)	Ganaera
P2TA	Intravenous	Immunomodulation	Infections cause by necrotising soft tissue	Phase 3	Atox Bio

a NA stands for not available.

Two lipopeptides, murepavadin (POL7080, NCT03409679) and surotomycin (NCT01597505, NCT01598311), successfully entered phase III clinical trials in the last five years. Murepavadin belongs to a novel class of antibiotics that target the LPS transport protein D. This lipopeptide is very potent specifically against *P. aeruginosa*-associated nosocomial pneumonia.^241,242^ Surotomycin is a calcium-dependent antibiotic that depolarises Gram-positive and Gram-negative bacterial membranes. The drug is currently being evaluated as an emerging therapeutic for *C. difficile*-associated diarrhoea.^243^

As we have summarised in this section, AMPs can be natural products, but they can also be designed *de novo*, or identified *via* the screening of large libraries, which will be discussed in the following sections.

## 9 Chemical synthesis of AMPs

Early AMP discovery relied on isolation from natural sources, usually requiring large quantities of raw biological material from which small quantities of pure peptide could be extracted.^244^ It is now possible for AMPs to be isolated on a large scale through recombinant DNA technology or through chemical synthesis. The choice of production method is often dictated by the size of the AMP to be synthesised, with larger peptides (>approximately 50 residues) becoming increasingly less practical to realise by chemical means.^245^ However, the recombinant expression of AMPs is generally considered to be more complex and labour-intensive than chemical synthesis, despite the lower production costs and lighter environmental burdens. Furthermore, the recombinant expression of some AMPs requires the incorporation of a large fusion protein to mask the toxicity of the peptide to the host cell, which must later be cleaved from the AMP and removed during purification.^246–248^ For these reasons, the chemical synthesis of AMPs is often the more practical approach, particularly since the advent of solid-phase techniques, which have made the process rapid, efficient, and reliable.^249–252^

Solid-phase peptide synthesis (SPPS), initially introduced by Merrifield, is the most commonly used method to produce peptides of small to medium size (up to 50 residues).^253–255^ SPPS involves attaching the C-terminal aa of the AMP to a polymeric solid support, usually *via* a cleavable chemical linker, followed by successive deprotections and couplings of the aa building blocks to enable peptide chain elongation. In this way, excess reagents and byproducts can be effectively removed by washing the solid support. Once the full sequence has been assembled, it can be cleaved from the resin to afford the desired peptide in high yield and purity.^250^

The chemical synthesis of AMPs presents several advantages compared to extraction from natural sources. Indeed, precise modification of AMP sequences is possible since each aa is sequentially added during SPPS, thus enabling the modulation and improvement of the antibacterial potency and the investigation of the structure–activity relationships (SAR). Futhermore, in addition to the 20 naturally occurring aa, nonnatural aa can also be inserted or substituted into the AMP sequence to improve its biological activity and stability.^256^ Many natural nonribosomally- and ribomally-synthesised AMPs have been the subject of SAR studies, facilitated by SPPS of the peptides and their analogues. The analysis of analogues of natural products is a key approach taken in drug discovery from primary screening to lead optimisation. The optimisation process requires the construction of a large number of analogues in order to identify new compounds with more desirable properties. Many reviews have summarised the SAR of known AMPs, such as polymyxins, gramicidin D and S, cecropins, magainins, defensins, and cathelicidins.^142,219,257–263^

While natural AMPs are structurally diverse and have promising antibiotic properties, they confer several disadvantages as peptide therapeutics, such as susceptibility to proteolytic degradation, potential toxicity to mammalian cells, and high costs associated with their industrial production. As such, many types of chemical modifications in AMPs have been developed with a view to improving their antimicrobial potency and reducing their susceptibility to proteolytic degradation. There are various chemical modifications that can be made to naturally-occurring AMPs, as well as synthetic AMPs, including substitution of one or more aa residues of the natural AMP template with other proteinogenic l- or d-residues, N-terminal acetylation, C-terminal amidation, peptide cyclisation, introduction of unnatural aa, PEGylation, lipidation, and the construction of hybrids.^180^ In the following sections, we will describe each type of modification and highlight examples where the modification has resulted in improvement or decline in the AMP activity.

### 9.1 Substitution with l- and d-proteinogenic amino acids

The most straightforward approach for improving the antimicrobial activity and selectivity of an AMP is to substitute one or more aa residues to other proteinogenic l-residues. For example, aa substitutions of the natural AMP magainin II (sequence H–GIGKFLHSAKKFGKAFVGEIMNS–OH) has led to the discovery of pexiganan (sequence H–GIGKFLKKAKKFGKAFVKILKK–NH_2_). By replacing selected neutral and anionic aa with cationic and hydrophobic residues, the antimicrobial activity of the resulting analogue pexiganan was improved, exhibiting potent broad-spectrum antimicrobial activity.^264,265^ Similarly, several other synthetic AMPs have successfully reached late stage clinical trials using l-aa substitutions. Examples include iseganan, omiganan, and P113, which were developed from protegrin, indolicidin, and histatin respectively.^266–269^

Alanine-scanning is a residue substitution scanning methodology in which peptide analogues are systematically made (or modelled computationally) with single Ala substitutions, enabling investigation of the functional role of each aa, and thus an understanding of the AMP SAR.^270–273^ This approach has been used on a 25-mer casein-derived AMP, which showed that the five C-terminal residues are important for its activity against *L. monocytogenes* and *C. sakazakii*.^271^ Similarly, this methodology has enabled the identification of food-derived peptides with antihypertensive activities that also exhibit potent antimicrobial activity against *S. aureus*, *M. luteus*, *E. coli*, and *C. albicans*.^272^ In another study, Ala-scanning applied to the lipopeptide octyl-tridecaptin A1 enabled the identification of key residues that were responsible for the formation of a stable secondary structure that impacted its activity against Gram-negative bacteria, including some MDR strains.^273^ More recently, Ala-scanning has enabled the investigation of the SAR of the AMP aurein 1.2, the shortest nature-derived AMP that was initially isolated from Australian bell frogs (*Ranoidea aurea*). In this study, the authors observed that a systematic substitution of each aa to an alanine residue resulted in analogues with reduced peptide helicity, but did not strongly affect the antimicrobial activity.^270^

Numerous studies have shown that l- to d-aa substitution of a peptide template can retain the original antimicrobial activity, while preventing proteolysis.^180,274^ However, partial substitution to d-aa of AMPs may result in a loss of α-helicity, depending on the position and the number of d-aa substituted.^275^ A complete substitution of l- to d-aa residues in the peptide sequence results in the enantiomer, whereas partial substitution yields different diastereomers.^276^ For example, Wang and co-workers isolated a natural lysine-rich AMP, known as MPI, from social wasp venom. They then synthesised the d-enantiomer of the peptide and observed that it was significantly more resistant to protease degradation.^277^ Furthermore, the d-peptide retained antimicrobial efficacy against Gram-positive and Gram-negative bacteria and fungi. However, the same study showed that the antimicrobial activity was lost when only the stereochemistry of the Lys residues were switched, which was attributed to destabilisation of the peptide's secondary structure.

In a study by the Hancock research group, the authors designed a library of short synthetic l-AMPs and their respective d-enantiomers. Two of the d-peptides, D-JK-5 (sequence H-vqwrairvrvir-NH_2_) and D-JK-6 (sequence H-vqwrrirvwvir-NH_2_), were shown to be more proteolytically stable than their l-enantiomers. Additionally, the d-enantiomers retained the anti-biofilm activity of their respective l-enantiomers in seven species and 30 strains of wild-type and MDR pathogens.^278^

Hodges and co-workers illustrated the impact of d-aa substitution on several properties of an AMP by performing a scan of the amphipathic α-helical AMP V681 (sequence Ac-KWKSFLKTFKSAVKTVLHTALKAISS-NH_2_). The researchers systematically replaced residues in the polar and non-polar faces with their d-enantiomer and then determined the antibacterial activities, secondary structures, and haemolytic activities of the resulting peptides. One of the analogues (V681) displayed 90-fold and 23-fold improved potency against Gram-negative and Gram-positive bacteria respectively. The authors also found that substituting l-aa for d-aa resulted in lower helical contents in buffer, as measured by CD spectroscopy. Changing the stereochemistry of a particular aa did increase the haemolytic activity in some cases, although the authors did not elaborate as to why this was not observed for all substitutions trialled.^279^

Although substituting l-aa for their d-enantiomers is a common method used to reduce the susceptibility of peptides to proteolytic degradation, it is important to note that d-aa lack specific recognition receptors in mammalian cells, which can impair AMP immunomodulatory activity (see Section 6.2).^180,280^

### 9.2 N-Terminal acetylation and C-terminal amidation

Modifications such as N-terminal acetylation and/or C-terminal amidation are two of the most common approaches used to increase the proteolytic stability of both naturally-occurring and synthetic peptides.^275^ N-Terminal acetylation is a protein modification frequently observed among eukaryotic and prokaryotic cells. Although N-terminal acetylation blocks the activity of aminopeptidases, thus improving the proteolytic stability of the peptide, it decreases the net positive charge by one, which can decrease the antimicrobial activity.^281–285^ For example, Chaudhary and co-workers developed several AMPs derived from MreB protein, a bacterial cytoskeleton protein found in non-spherical bacteria beneath the bacterial cytoplasmic membrane. The peptide fragment MreB_1-9_ carried a net charge of +4 and displayed good antimicrobial activity against both Gram-positive and Gram-negative bacteria, as well as *C. albicans*. The authors showed that acetyl-capping of the N-terminus retained the peptide's antimicrobial activity against *C. albicans* but rendered it less effective against *P. aeruginosa* and *S. aureus*.^283^

C-Terminal amidation is also a common post-translational modification widely observed in natural AMPs.^56,286^ In contrast to N-terminal acetylation, C-terminal amidation has been shown to improve the antimicrobial efficacy of many membrane-disrupting AMPs, likely due to increased α-helix stability at the peptide-membrane interfaces, which enables greater membrane disruption and pore formation.^57,59,275,287,288^ For instance, aurein 2.5, an amphibian AMP naturally amidated at the C-terminus, showed better efficacy than its non-amidated analogue against *K. pneumoniae* in a study by Phoenix and co-workers.^289^ Furthermore, they recently showed that amidated aurein 2.6 and aurein 3.1 have greater propensity to form a stable α-helix than their non-amidated analogue by circular dichroism (CD) spectroscopy and MD simulations.^287^

Nevertheless, studies have shown that simultaneous N-terminal acetylation and C-terminal amidation on AMPs is favourable for their proteolytic stability. For example, an AMP derived from human apolipoprotein B that underwent simultaneous N-acetylation and C-amidation exhibited more than 4-fold increased proteolytic stability compared to the unmodified AMP after incubation with 10% fetal bovine serum for 1 hour.^290^ Similarly, Ovchinnikova and co-workers showed that the proteolytic stability of tachyplesin I was enhanced compared to the unmodified AMP following N-acetylation and C-amidation.^291^

### 9.3 Unnatural amino acids

Another possible chemical modification that can increase the proteolytic stability and antimicrobial efficacy of AMPs is the incorporation or substitution of natural aa with non-natural aa or aa analogues. For example, ornithine, 2,4-diamino-butyric acid (Dab), and 2,3-diamino-propionic acid (Dap) can be used in place of Lys to vary the number of side chain methylene units.^274,275^ Vogel and co-workers have recently demonstrated that substitution of Lys to Dap (from four to one methylene unit) in Trp-rich peptides increased their antimicrobial efficacy 4-fold against *E. coli*, likely due to increased membrane permeabilisation.^292^ Petraccone and co-workers recently designed a small library of cationic synthetic peptides containing unnatural aa such as 2-naphthyl-l-alanine and *S-tert*-butylthio-l-cysteine. These peptides were shown to display high activity towards a broad spectrum of pathogens and increased proteolytic stability.^290^

**Fig. 8:**
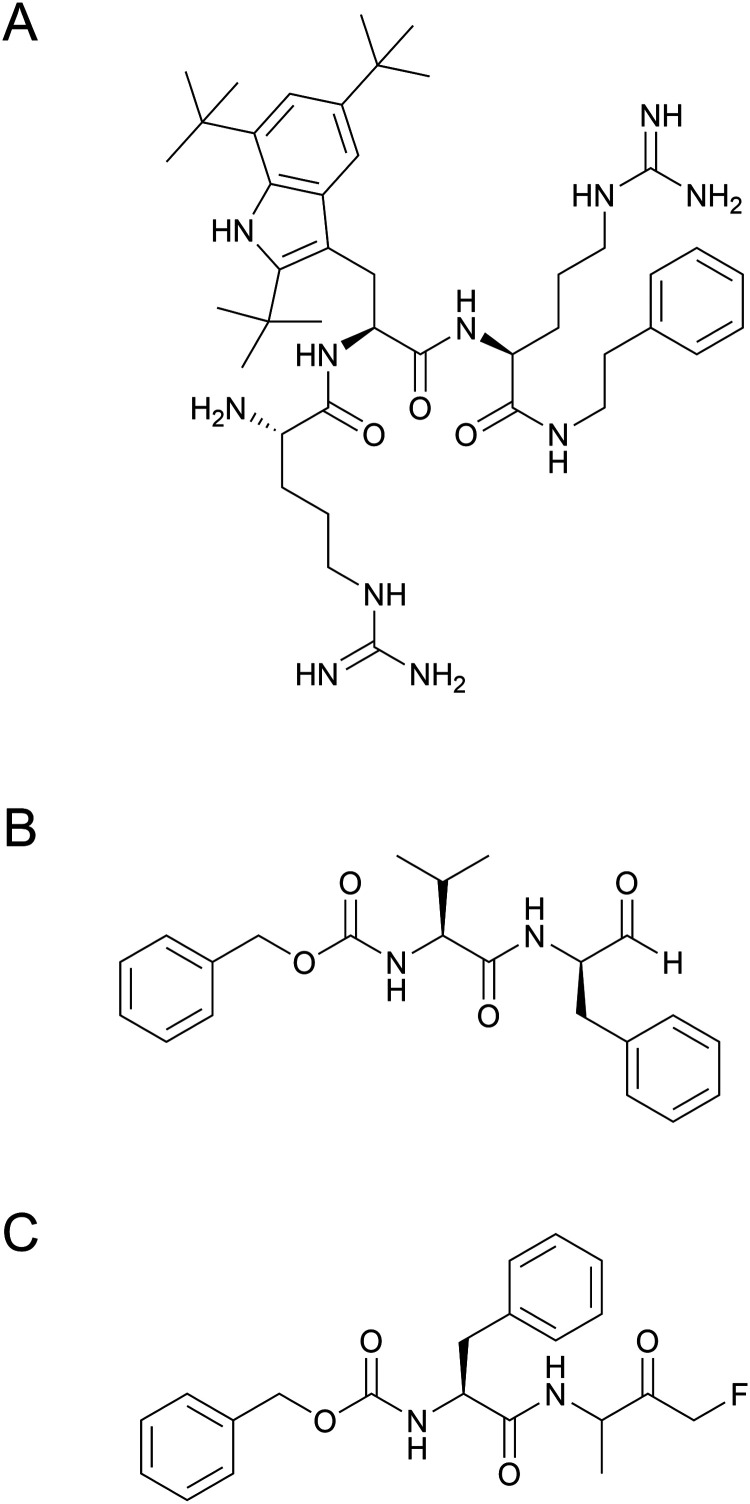
The structure of LTX-109 (A) and the peptidomimetics reported by Hiromatsu and co-workers (B) and (C).^296^

LTX-109 (Fig. 8A), a tri-peptide comprised of a lipophilic, unnatural tryptophan residue flanked by two arginine residues, has highlighted how successful the use of unnatural aa can be for the development of therapeutic AMPs.^293,294^ Developed by Lytix Biopharma, LTX-109 completed Phase II clinical trials for efficacy and safety in 2014, although no trials have been registered since.^295^LTX-109 causes membrane disruption of *E. coli* and *S. aureus*, the archetypal AMP mechanism of action.

Finally, Hiromatsu and co-workers repurposed Cbz-protected dipeptide calpain inhibitors through incorporation of unnatural C-terminal aa containing fluoromethyl ketone and aldehyde moieties (Fig. 8B and C).^296^ These dipeptide AMPs displayed significant bacteriostatic activity against *Chlamydia trachomatis*, although bacterial growth resumed after the treatment ended.

### 9.4 PEGylation

Polyethylene glycol (PEG) moieties are commonly attached to peptide-based agents to improve their solubility, pharmacokinetics, and half-life.^276^ However, the addition of PEG chains to AMPs has not been extensively explored. PEGylation of AMPs could decrease the binding of the peptides to the bacterial membrane, thereby reducing their antimicrobial efficacy. However, PEGylation may also reduce cytotoxicity and haemolysis.^180,297–299^ For example, PEGylation of magainin and the cyclic peptide tachyplesin significantly decreased the cytotoxicity of the peptides but also resulted in decreased activity against *E. coli* and *S. epidermidis* compared to the native peptides.^297,298^ Malmsten and co-workers have demonstrated that PEGylation of an AMP, KYE28, led to partial loss of antimicrobial activity, which correlated with increasing length of the PEG chain. However, the PEGylated peptide showed significantly decreased haemolysis and improved selectivity against bacteria in a mixture of blood cells and bacteria.^299^

### 9.5 Lipidation

Lipidation of AMPs has been shown in several studies to efficiently increase the activity of AMPs. For example, lipidation of the C- or N-terminus of a short linear (Arg-Trp)_3_ peptide resulted in improved activity against *P. aeruginosa* and *A. baumannii*, as shown in a study by Metzler-Nolte and co-workers.^300^

**Fig. 9:**
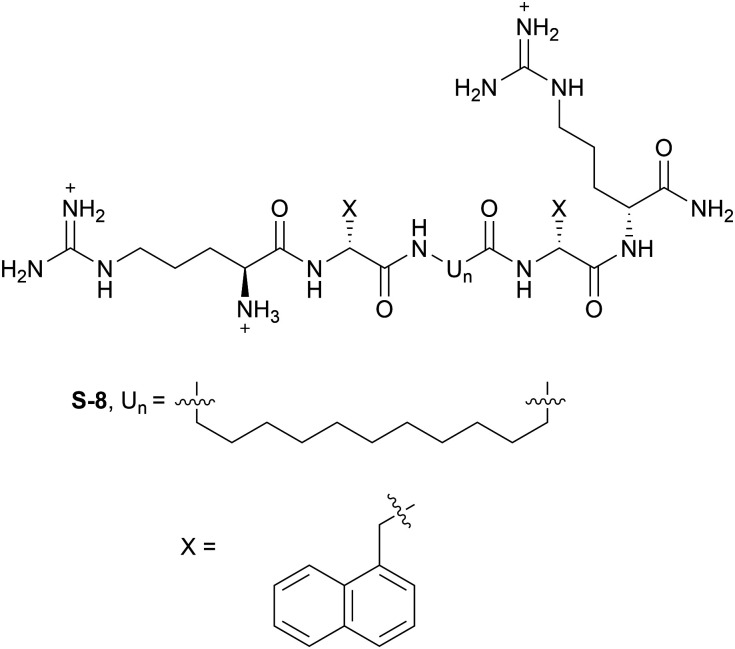
An antimicrobial lipopeptide, S-8, reported by Mukhopadhyay and co-workers.^302^

Shai and co-workers have developed a series of 4-mer lipopeptides of sequences C_*n*_-KXXK-NH_2_ (where X = L, A, G, K, or E; and *n* = 12, 14, or 16), each containing a single d-aa residue and an N-terminal fatty acid chain.^301^ Biological assessment of these lipopeptide AMPs revealed that even though the peptides were short, they possessed potent antimicrobial activity, and the different analogues displayed vastly different specificities against the bacterial strains tested. Very few of the peptides displayed haemolytic activity at the concentrations tested. Mukhopadhyay and co-workers reported lipopeptide AMPs containing lipophilic aryl and alkyl unnatural aa residues flanked by arginine and 1-naphthyl-d-alanine residues (Fig. 9).^302^ The lead peptide, S-8, contained an ‘internal’ fatty acid chain and displayed extremely low MIC values against clinically-relevant staphylococcal strains *via* membrane-depolarisation. S-8 also displayed minimal haemolysis and was active against vancomycin-resistant biofilms. Both these examples outline how various simple modifications can be incorporated into an AMP simultaneously to fine tune its properties.

Building on previous reports of antimicrobial lipopeptide AMPs, Toth and co-workers reported short, branched lipopeptide AMPs comprising different combinations and sequences of 2-aminododecanoic acid and lysine residues.^303,304^ Analogues with multiple fatty acid residues and higher net positive charges displayed good antimicrobial activity against Gram-positive strains, minimal toxicity towards human cell lines, and improved trypsin stability.

Although AMP lipidation has generally been demonstrated to be a good strategy to improve the antimicrobial activity, it also increases the affinity of the peptide for any biological membrane. As such, a loss of specificity for bacterial membranes and higher haemolysis can be expected. Nevertheless, this drawback could be overcome by combining lipidation with other chemical modifications.^305–308^

### 9.6 Cyclisation

Macrocylic peptides are commonly found among naturally occurring AMPs, some of which have been FDA approved and used in the clinic (Section 8). Macrocyclisation occurs either through head-to-tail, backbone–backbone, side chain–backbone or side chain–side chain linkages (Fig. 2D). When macrocyclisation is afforded by a side chain-to-side chain linkage that is not present in a naturally occurring peptide, this is usually referred to as peptide stapling and will be discussed separately in Section 14.1. However, when the side chain–side chain linkage is disulfide formation between two introduced cysteine residues that are not present in the natural peptide, it will be considered here. This classification is however flexible and can be adjusted for purpose.

Many studies have shown that cyclic peptides generally demonstrate favourable properties as antimicrobial agents, which is attributed to an increase in proteolytic stability, and conformational rigidity. Additionally, cyclisation also enhances cell selectivity leading to reduced host cytotoxicity.^56,275^ Peptide macrocycles attract considerable attention as potential AMPs due to their synthetic accessibility. Both the peptide sequence and nature of the cyclisation are important parameters for the optimisation of antimicrobial activity and human toxicity. The potential benefits of macrocyclic peptides have driven recent studies aiming to improve oral bioavailability, which is typically poor for peptide drugs. The potential of macrocyclic drugs as candidates for different clinical applications has been addressed in recent reviews.^309,310^

In this section, we summarise several examples of antimicrobial peptides that have been cyclised through different chemical strategies, with a view to achieving improved antimicrobial activity and stability and reduced haemolytic activity.

Ghadiri and co-workers developed several small cyclic d,l-α-peptides of even numbers of aa (six or eight) and alternating l-Trp and d-Leu residues that were able to self-assemble into tubular structures in membrane environments. These peptides were cyclised head-to-tail *via* peptide bond formation between the N-terminal amine and the C-terminal acid. The cyclised peptides displayed strong activities against Gram-positive MRSA and Gram-negative *E. coli*. However, the afforded polytryptophan cyclic peptides displayed haemolytic activity.^311,312^

In a similar study, Bienert and co-workers demonstrated that head-to-tail cyclised analogues of Arg/Trp-rich peptides (based on the linear sequence Ac-RRWWRF-NH_2_) displayed up to 16-fold greater antimicrobial activity against *B. subtilis* and *E. coli* compared to their linear counterparts. However, cyclisation also resulted in increased haemolysis.^313^ Parang and co-workers also synthesised a head-to-tail cyclised AMP using 4 hydrophilic and 4 hydrophobic residues, with cyclisation being conducted after cleavage of the peptide from the resin. The peptide (head-to-tail cyclised sequence, RRRRWWWW) showed more potent activity against MRSA compared to its linear counterpart.^314^

Inspired by the intramolecular disulfide bonds between cysteine residues in the natural cyclic defensins, Lai and co-workers successfully developed an AMP that was cyclised *via* disulfide bond formation. Two cysteine residues were introduced into cathelicidin-BF15-a3, a peptide derived from the snake venom of *Bungarus fasciatus*. The cyclic peptide showed excellent activity against *P. aeruginosa* and *A. baumannii*, low haemolysis, high stability *in vivo*, and low propensity to induce resistance.^36^

**Fig. 10:**
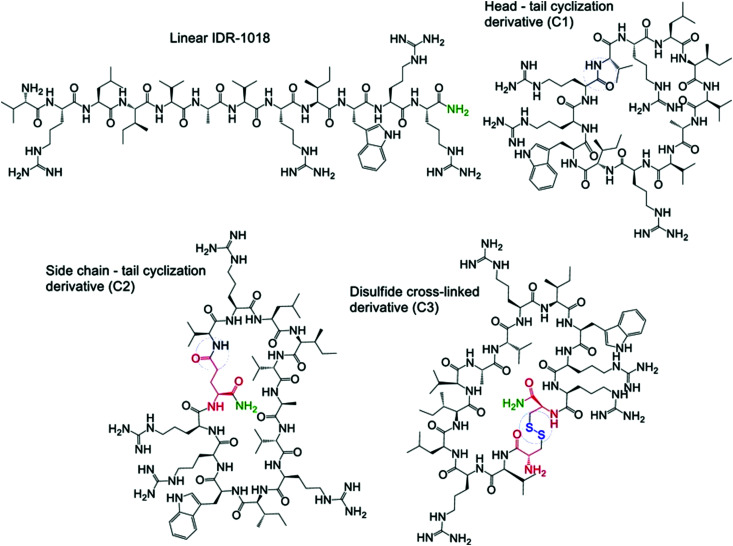
Linear AMP IDR-1018 and its cyclic analogues. Additional residues are in red, amidation at the C-terminus in green, and C3 disulfide bridge in blue. Figure reproduced from Hancock and co-workers with permission from The American Chemical Society, Copyright (2020).^315^

More recently, Hancock and co-workers investigated the effect of different cyclisation strategies on the antimicrobial, antibiofilm, and immunomodulatory properties of the linear AMP IDR-1018. The cyclisation approaches employed were head-to-tail cyclisation; glutamate side chain-to-tail cyclisation by amide formation between the N-terminal amine and the γ-carboxylate of glutamate; and cyclisation *via* disulfide linkage through cysteine side chain residues introduced at both termini (Fig. 10). Among the three cyclisation strategies, the macrocycle resulting from side chain-to-tail cyclisation exhibited a strong ability to suppress inflammation and significantly reduced bacterial loads in a high-density *S. aureus* murine skin infection model.^315,316^

### 9.7 Hybrid AMPs

**Fig. 11:**
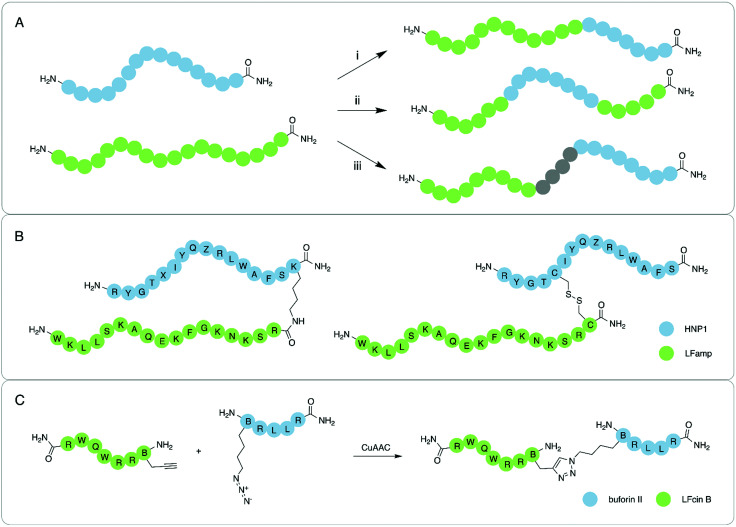
(A) Different strategies for hybrid preparation. (i) Sections of parent peptides (blue and green) can be joined directly at their termini, (ii) the middle section of one of the parent peptides can be replaced by a sequence from the other parent peptide, or (iii) sections of the parent peptides can be joined using a short peptide linker (grey). (B) Hybrids from Ptaszynska and co-workers joined *via* peptide side chains. Spheres represent individual aa where blue spheres are of human neutrophil protein 1 (HNP1) and green spheres of lactoferrampin. The amide-linked hybrids used analogues of HNP1 with X being either l-α-aminobutyric acid (Abu) or acetaminoethyl cysteine (C-Acm) while the disulfide-linked hybrid had a cysteine residue at the same position. In all hybrids, the HNP1 component also contained 2-aminobenzoic acid (2-Abz, labelled Z). (C) Side chain–side chain joined hybrid (chimera) using unnatural aa, labelled B, for CuAAC click chemistry.

Chemical modification is not the only way of optimising the activity and physicochemical properties of AMPs. Full AMPs or AMP fragments are frequently combined into a single peptide, often called a ‘hybrid’ or ‘chimeric’ peptide, with the hope that this will lead to an increase in potency, the enhancement of selectivity, reduction in cytotoxicity, or a dual mode of action.^317,318^ While the term hybrid or chimeric peptide can be used for a variety of combined structures, including peptidomimetics with mixed backbones, here we will consider only hybrid peptides formed by combination of two established AMPs (Fig. 11A). We will illustrate several examples of hybrids that have been modified and synthesised in most cases by SPPS. However, hybrids can also be prepared by recombinant expression.^319^ The examples given have been selected to highlight how the properties of the individual parent peptides can affect the function of the resulting hybrid.

The research of Shi and co-workers has focused on hybrid peptides that acquire selectivity for a particular bacterial strain from one of the peptide components and potency from the other. Initially, they produced such a hybrid by combining the 18-mer AMP novispirin G10 with a short 12-mer peptide KH, the latter of which shows preferential binding for *Pseudomonas* spp. over *E. coli* and *S. mutans*.^320^ For each of the three bacterial species, the hybrid of G10 attached to the C-terminus of KH (G10KHc) did not show a significant change in MIC compared to novispirin G10. Of the three tested bacterial strains, G10KHc killed *P. mendocina* most efficiently, with the lowest MIC and fastest killing kinetics. In a separate co-culture experiment, the peptide preferentially killed *P. mendocina* over *S. mutans*. In another study, the research group used the same approach to target *S. mutans*, a bacterium that causes dental cavities. By combining an AMP derived from novispirin G10 with a subsection of competence stimulating peptide (CSP), a bacterial pheromone, they created a hybrid, C16G2.^321^ This hybrid AMP showed preferential killing of *S. mutans* over other staphylococcal strains both in planktonic and biofilm-embedded states. In addition, when tested on saliva-derived microbial cultures, C16G2 not only preferentially killed the target strain, but also eliminated other malicious bacterial strains co-dependent on *S. mutans*.^322^ This provided space for the growth of more beneficial strains of bacteria, while maintaining the overall bacterial count. The group were also able to demonstrate the application of their method to the combinatorial synthesis of a library of over 120 hybrid AMPs from a pool of various targeting peptides, linkers, and AMPs.^323^ Some members of the library showed selective activity against *S. mutans*.

Shan and co-workers improved properties of RI16, a fragment of the porcine cathelicidin PMAP-36, by hybridisation with an anti-biofilm peptide FV7.^324^ FV7 is a conserved sequence from the most potent anti-biofilm peptides identified from a peptide library screen and subsequent hit optimisation.^190^ An internal section of the RI16 sequence was replaced with that of FV7, aiming to make RI16 less amphipathic and thus introduce anti-biofilm properties, as imperfect amphipathicity has been shown to affect the mechanism of action of AMPs.^325^ The resulting hybrid R-FV-I16 showed improved MIC values against Gram-positive and Gram-negative bacterial strains compared to both parent peptides, a higher therapeutic index, thermal stability at 100 °C, and both membrane-disrupting and anti-biofilm effects. However, its activity was weakened when cations or proteases were introduced to the growth media.

In 2017, the group also developed hybrids of FV7 with the AMPs LL-37 (LL), magainin II (MA) and cecropin A (CE) by attaching the anti-biofilm peptide to the N-terminus of the AMPs.^326^ The resulting hybrids (FV-LL, FV-MA and FV-CE, respectively) all showed improved activity against a panel of Gram-positive and Gram-negative bacteria compared to the parent peptides. All hybrids disrupted membranes of *E. coli* and *S. aureus*, as demonstrated by electron microscopy. Among the three hybrid peptides, FV-LL had the lowest MIC value, the lowest rate of haemolysis, and was the most potent membrane permeabilisator. When tested for anti-biofilm properties on *P. aeruginosa*, FV-LL showed higher biofilm clearance than the parent FV7. Like R-FV-I16, all the hybrid peptides showed a slight decrease in activity in the presence of cations, but good thermal stability. The group has also reported hybrids that combined LL-37 with indolicidin, cecropin P1, or rat neutrophil peptide 1; and PRW4 with fowlicidin 2, protegrin 3, or tritrpticin.^327,328^

Mor and co-workers published a hybrid of RNA-III inhibiting peptide (RIP) and DD_13_, a derivative of the AMP dermaseptin.^329^ RIP is a short peptide that can intercept staphylococcal quorum sensing and thus disrupt biofilm formation. The resulting DD_13_–RIP hybrid was tested *in vivo* on vascular grafts implanted in mice. The grafts were soaked in a solution of the peptide before being implanted and injected with either MRSA or *S. epidermidis*. The implants were removed after a week and the number of CFUs established. The hybrid showed a significant reduction in bacterial load by up to 6 orders of magnitude relative to the saline-treated graft and outperformed the parent peptides. The DD_13_–RIP hybrid was also more effective than rifampicin, an antibiotic control chosen for its frequent clinical use against staphylococci. However, when administered in combination with rifampicin lower concentrations of both the parent peptides and the hybrid peptide were required to eradicate bacterial colonies than treatment with just the peptides on their own.

Cardarelli and co-workers reported a hybrid of the cell penetrating peptide (CPP) Tat_11_ with CM_18_, which is itself a hybrid of the AMPs cecropin A and mellitin.^330^ Mellitin is a potent AMP that also shows a high haemolysis rate and is frequently used as a reference peptide in bacterial growth assays.^331^ Cecropin–mellitin hybrids maintain the potency of mellitin with a significantly reduced rate of haemolysis.^332^ CPPs are a group of short (typically <35 aa) and structurally diverse peptides which are able to translocate cellular membranes in a non-disruptive manner.^333^ Among the most widely-studied CPPs are protein-derived Tat and penetratin, as well as synthetic polyarginine sequences.^333,334^ The CM_18_–Tat_11_ hybrid showed an improved bactericidal activity against *S. aureus* compared to the parent peptides; however, the hybrid was not developed as an antimicrobial. The intended primary use of the hybrid was to trigger the release of endocytosed vesicle contents into the cytosol of eukaryotic cells. This property was tested by loading vesicles with a variety of fluorescent substrates, including Tat_11_–EGFP, calcein, dextrans, and luciferase-encoding plasmids. In all cases, treatment of the loaded cells with CM_18_–Tat_11_ led to increased diffusion of the fluorescence across the cells. CM_18_ and Tat_11_ alone were unable to cause a diffusion of fluorescence. The researchers hypothesised that the addition of Tat_11_ to the CM_18_ sequence likely changed the mechanism of membrane-disruption from pore formation to a detergent model (see Section 6.1), which led to a loss of integrity of the vesicular membrane.^335^ Although it was not primarily intended for antibacterial use, attaching CPPs to AMPs still presents an opportunity for expanding the potential of AMP therapeutics. For example, CPPs may facilitate an improved ability for other therapeutics to penetrate cells in order to access intracellular pathogens (see Section 15.3).

Instead of combining two sequences by forming a single peptide backbone, some groups have prepared hybrids by attaching two parent peptides through their side chain residues. One such example has been reported by Ptaszynska and co-workers, who produced hybrids of bovine lactoferrampin (LFamp) and analogues of a fragment of human neutrophil peptide 1 (HNP1).^336^ The researchers produced three chimeras, two where the ε-amino group of Lys in HNP1 was connected to the C-terminus of LFamp by an amide bond, and one where the two peptides were joined by a disulfide bridge between two cysteine residues (Fig. 11B). In the case of the amide-linked hybrids, the HNP1 analogue was synthesised on resin first, then the orthogonal protecting group (Mtt) on the C-terminal lysine was removed and the LFamp sequence constructed from this amine handle. The full construct was then released from the resin. For the disulfide-linked hybrid, the constituent peptides were synthesised separately and cleaved from their respective resins. The cysteine residue on the HNP1 analogue was activated with 2,2′-dithiopyridine in solution at which point LFamp, containing a C-terminal cysteine, was mixed with the activated HNP1 to yield the hybrid. The amide-linked hybrids showed improved activity against both Gram-positive and Gram-negative bacteria, outperforming the mixture of parent peptides and the parent peptides alone. The same was true for the disulfide-linked hybrid, but only for the Gram-positive strains.

The example above was inspired by the work of Veerman and co-workers on LFchimera, a chimera of the lactoferrin fragments lactoferricin (LFcin) and LFamp.^337^ LFcin was synthesised on resin with an orthogonally protected C-terminal lysine, which was subsequently deprotected and LFamp then synthesised from the newly exposed ε-amino group. The resulting LFchimera showed improved activity against both Gram-positive and Gram-negative bacteria compared to the parent peptides and its potency was not significantly affected by sodium cation concentration. Taweechaisupapong and co-workers produced an analogue of LFchimera, LFchimera2, which comprised LFcin and a truncated analogue of LFamp in which its last three N-terminal residues were removed. Both the original and the new chimera were tested against Gram-negative *B. pseudomallei*, and although LFchimera2 outperformed the parent peptides, it did not give an improvement over the original LFchimera.^338^LFchimera was later also shown to reduce biofilms of *B. pseudomallei*.^339^

Matsuzaki and co-workers studied another disulfide-linked chimera formed between magainin II and PGLa to study the synergistic interaction between the two peptides.^340^ The synergy had been attributed to the formation of a heterodimer between the peptides, so a covalent hybrid was used to interrogate this proposed dimer. The method of synthesis of this hybrid was not specified. Both a physical mixture of the parent peptides and the hybrid displayed a similar increase in potency against *E. coli* and *S. epidermidis* compared to parent peptides alone, with the hybrid showing more sustained suppression of growth of *S. epidermidis* than the mixture, but at the same time significantly increased haemolysis. The exact mechanism of the synergistic interaction of magainin II and PGLa with membranes is still the subject of further studies, with new insights recently published by Vacha, Pabst and co-workers.^341,342^

Finally, click chemistry in combination with unnatural aa side chains can be used to join two smaller peptides into a peptide chimera, such as in the case of fragments from LFcin B and buforin II (Fig. 11C).^343^ Alkyne- and azide-containing aa were introduced at the N-termini of LFcin B and buforin II, respectively, and the peptides were joined by a CuAAC reaction. Compared to the parent peptides, the resulting chimera showed improved activity against *E. coli* and *S. aureus*.

## 10 Semi-synthetic AMPs

To synthesise analogues of complex natural antimicrobial compounds, some groups have adopted a semi-synthetic strategy. Semi-synthesis is a chemical approach whereby a complex starting material is isolated from natural sources and subsequently chemically modified to yield novel analogues.

**Fig. 12:**
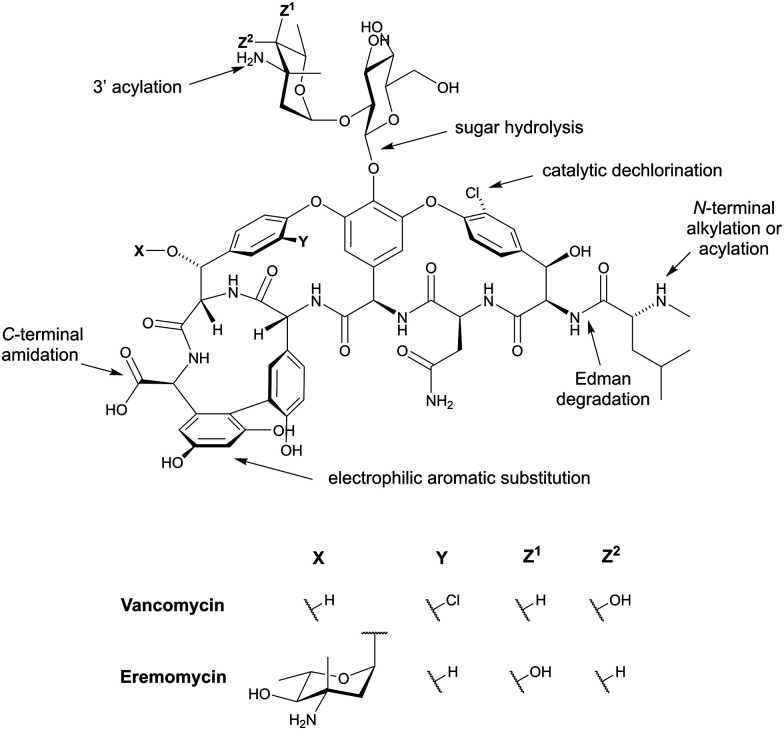
Frequently reported semi-synthetic modifications of glycopeptides vancomycin and eremomycin.

One of the most frequently semi-synthetically modified structures are glycopeptides, which include eremomycin and the well-known vancomycin. In a recent review, Olsufyeva and Yankovskaya have discussed four different types of antibiotics that were prepared using semi-synthesis, with glycopeptides being one of the studied groups.^344^ The authors described the different chemical modifications that have been reported for glycopeptides, including terminal carboxylate amidation, acylation of the sugar 3′ amino group, sugar hydrolysis, Edman degradation of the N-terminal aa, and N-terminal acylation or alkylation (Fig. 12). Other modifications have been reported in the literature, such as aminomethylation, bromination, iodination, attachment of selenocysteine on position 4 of the C-terminal resorcinol ring, or Pd/C-catalysed dechlorination.^345–348^ Semi-synthetic glycopeptides include the FDA-approved drugs such as oritavancin, telavancin, and dalbavancin.^349,350^ We direct the reader to the aforementioned review and references therein, as well as the review by Marschall, Cryle and Tailhades for more detail.^344,351^

Another frequently semi-synthetically modified AMP is the lanthipeptide nisin. Its modifications often include proteolytic digestion followed by expansion of the fragments by attachment of other constructs such as vancomycin, fragment mimics, lipophilic chains, or pore-forming peptides.^352–355^ Finally, cyclic peptidic scaffolds are also often modified after their isolation from natural or bioengineered sources. Examples include polymyxins or lipodepsipeptide daptomycin, whose ‘tail’ aa and lipophilic groups were altered, or depsipeptides ramoplanin and telomycin, whose acylation patterns were modified.^356–360^

## 11 Architectural synthetic AMPs

In addition to the promising synthetic and semi-synthetic approaches to producing compounds that mimic the properties of AMPs that we have discussed in the previous section, researchers have also developed novel AMPs with various creative architectures. Here, we consider ‘peptide architecture’ as the shape of a single peptide molecule, whose structure has been carefully designed by the authors.

In this section, strategies used to build multimeric peptides with antimicrobial properties will be described, such as the SPPS of peptide dendrimers and ring-opening polymerisation used to synthesise polypeptides.

### 11.1 Antimicrobial peptide dendrimers (AMPD)

**Fig. 13:**
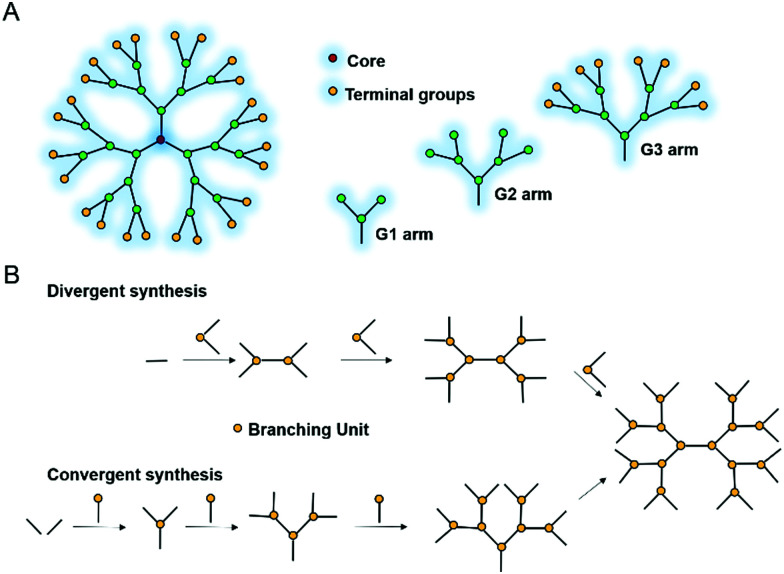
(A) Representation of G1, G2, and G3-generation dendrimers. Red: dendrimer core, orange: terminal groups, green: G1, G2, and G3 arms. (B) Divergent and convergent synthetic paths for building dendrimers.

The word “dendrimer” originates from the Greek, *dendron* for “tree” and *meros* for “part”. Generally, dendrimers are highly branched molecular trees, from which multiple functionalities can be displayed on the surface (multivalency), with a small hydrodynamic volume.^361^ In dendritic topology, the dendrimer comprises three major architectural components: a central dendrimer core; an inner shell composed of several layers labelled G1, G2, G3, *etc.*, where G stands for generations; and finally an outer shell containing the terminal functional groups. Dendrimer generations represent the number of branching points (or focal points) between the core and the surface. For instance, first (G1), second (G2), third (G3), fourth (G4)-generation dendrimers consist of dendrimers that contain one, two, three, four branching points between the core and the surface respectively (Fig. 13A).^361,362^

Two approaches are used to build dendrimers, namely divergent or convergent synthesis (Fig. 13B). Divergent synthesis involves building the dendrimer from the central core to the outer branches. By using this method, Smith and co-workers first achieved the synthesis and characterisation of polyaminoamine (PAMAM) dendrimers.^363^ In contrast, the convergent approach begins from the outer branches and converges to the inner core. This strategy was first reported by Fréchet and co-workers, who used this method to obtain aromatic polyether dendrimers.^364,365^

Peptide dendrimers (PD) form a unique class of dendrimer that contain only aa linked by amide bonds. The first PD was described by Denkewalter and co-workers, using lysine residues as their branching unit.^366,367^ PDs are believed to mimic natural peptides, proteins, and enzymes due to their unique shape and aa composition.^368^ They have been widely reported as drug delivery agents, antiviral agents, synthetic vaccines, and as agents for wound treatment.^368–376^ Antimicrobial peptide dendrimers (AMPDs) have recently attracted considerable attention as a novel class of synthetic AMPs, and the scaffold of a PD offers several advantages compared to linear peptides including greater stability, multivalency and a reduced risk of peptide aggregation during synthesis. This type of scaffold can also be easily modified. There are reports of PDs being functionalised with sugars, lipids, and fluorophores, and these modified scaffolds have been used in a large range of biological applications.^377–380^ We refer the reader to a comprehensive review by Haridas and co-workers about the role of peptide and protein dendrimers in various biological applications.^362^

Several research groups have tried to amplify the antibacterial properties of AMPs by taking advantage of the multivalency of PDs. As such, several examples of PDs will be described, highlighting their antimicrobial efficacy.

**Fig. 14:**
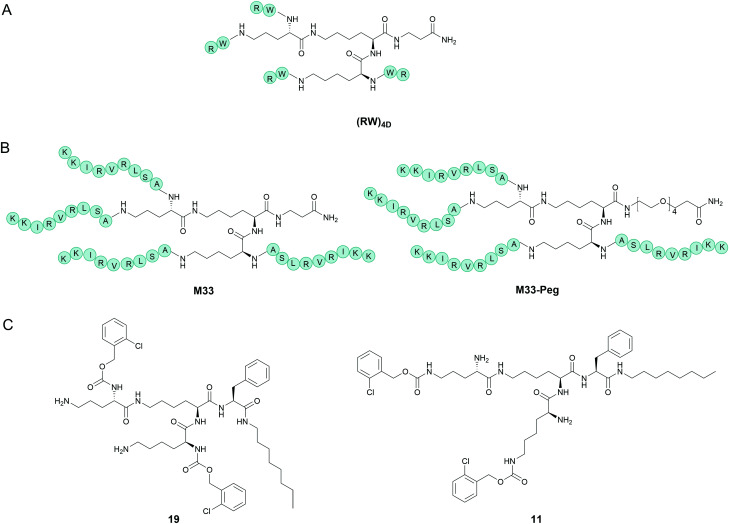
Structure of the peptide dendrimers (RW)_4D_ (A), M33 and M33-Peg (B), and 19 and 11 (C).

Kallenbach and co-workers built a first-generation (G1) PD by assembling four peptides on a tri-lysine core. The peptide dendrimer, referred to as (RW)_4D_, was appended at all four free amino groups with two different short dipeptides (either RW or WR). The short peptide sequences were inspired by the natural peptide indolicidin (H-ILPWKWPWWPWRR-NH_2_) and tritrpticin (H-VRRFPWWWPFLRR-OH) (Fig. 14A). This AMPD exhibited some membranolytic activity against ampicillin- and streptomycin-resistant *E. coli* and an MDR strain of *S. aureus*.^381^ Similarly, the Pini and the Bracci groups replaced the peptide sequence with KKIRVRLSA, resulting in the PD refered to as M33. This compound exhibited antimicrobial efficacy with and without PEGylation at the C-terminus of the PD (M33-Peg) and with different counter ions (Fig. 14B). The PD also displayed the ability to complex LPS to prevent septic shock *in vivo*.^382–384^

Using a similar tri-lysine core, the Urbańczyk-Lipkowska group developed a series of cationic PDs and identified two PDs, 19 and 11, that showed broad spectrum activity, including antifungal properties. In these constructs, the C-terminus of the branching lysine carried C_8_ lipidic chain, whereas the N^α^ or N^ε^ groups were appended with 2-chlorocarbobenzoxy groups (Fig. 14C).^385^

**Fig. 15:**
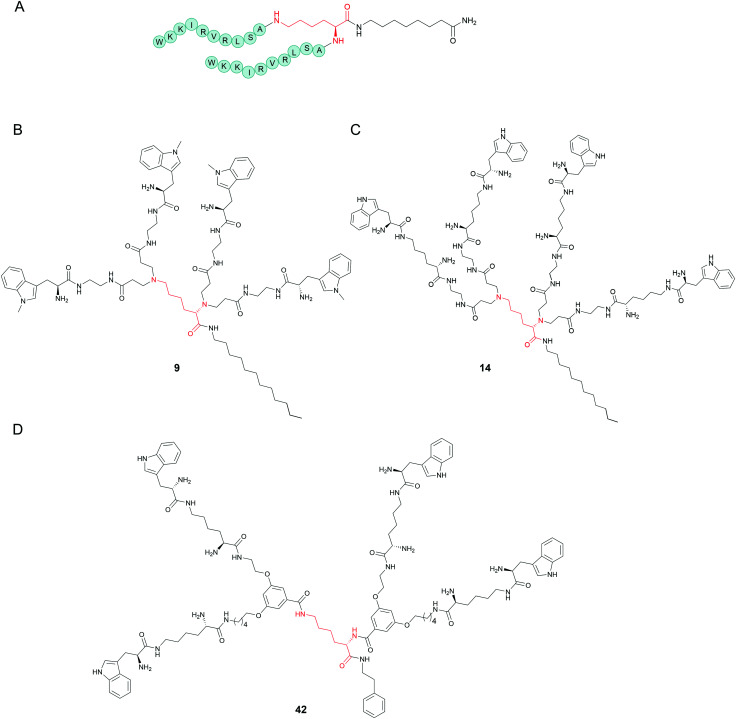
Structure of the PDs with a monolysine-core, such as lipodimeric peptide dendrimer reported by Rinaldi and co-workers (A), 4 (B), 14 (C) and 42 (D). The lysine at the core is in red.

Instead of a tri-lysine core, Rinaldi and co-workers constructed a lipodimeric PD, where only one lysine was used as a starting point to grow the PD, thus forming its core. The C-terminus of the lysine residue was attached to 8-aminooctamide, whereas the N^α^ and N^ε^ groups were used to attach the desired peptide (sequence WKKIRVRLSA or KWKIRVRLSA), yielding a first-generation PD (Fig. 15A). The peptide sequences contained alternating hydrophilic and hydrophobic aa, which enabled the AMPD to adopt a β-sheet structure in the presence of anionic vesicles as shown by CD spectroscopy and MD simulations. Furthermore, these PDs displayed enhanced antibacterial activity against *E. coli* and *S. aureus* compared to monomeric linear peptides.^386^

Similarly to Rinaldi and co-workers, Urbańczyk-Lipkowska and co-workers built a range of amphiphilic Trp-rich PDs, with a monolysine core, and variable structures and hydrophobicities to test their activities against *Candida* cells and to broaden their activity spectrum.^387,388^ First, PDs 9 and 14 (Fig. 15B and C) were identified and both displayed highly efficient inhibition of *C. albicans* growth. The authors used a lysine residue, where the C-terminus carried a dodecyl lipidic chain, and the N^α^ and N^ε^ groups were appended with methyl acrylate. The resulting branches were further extended with ethylene diamine followed by the coupling of 1-methyltryptophan to yield 9. In construct 14, the branches were further extended with additional lysine residues before the coupling of the tryptophan residues. Both compounds are G2 PDs.^389^

In order to broaden the spectrum of the PD antimicrobial activity, the authors constructed another series of amphiphilic dendrimers, where lysine or lysine-tryptophan dipeptides were displayed at the surface of the PDs. In this series, the authors identified AMPD 42 that exhibited superior antimicrobial activity against antibiotic-resistant *E. coli* clinical isolates. The PD was designed with a hydrobic core consisting of a monolysine, the C-terminus was coupled to benzylethylamine (PEA), tryptamine (TA), dodecylamine (DDA) or a Trp-OMe (W-OMe), and the lysine N^α^ or N^ε^ groups were used as the first branching unit to directly attach a second branching unit of variable length (Fig. 15D). The coupling of four lysines or lysine-trytophane dipeptides on the second branching unit resulted in a G2 PD.^388^

The Reymond group developed several PDs that showed remarkable activity against many Gram-negative bacteria, including the opportunistic Gram-negative pathogen *P. aeruginosa*. These PDs were assembled *via* SPPS from proteinogenic aa using diamino acids (*e.g.* lysine) as branching units to generate protein-like structures. In this way, the length and the composition of the PD branches can be fine-tuned.

**Fig. 16:**
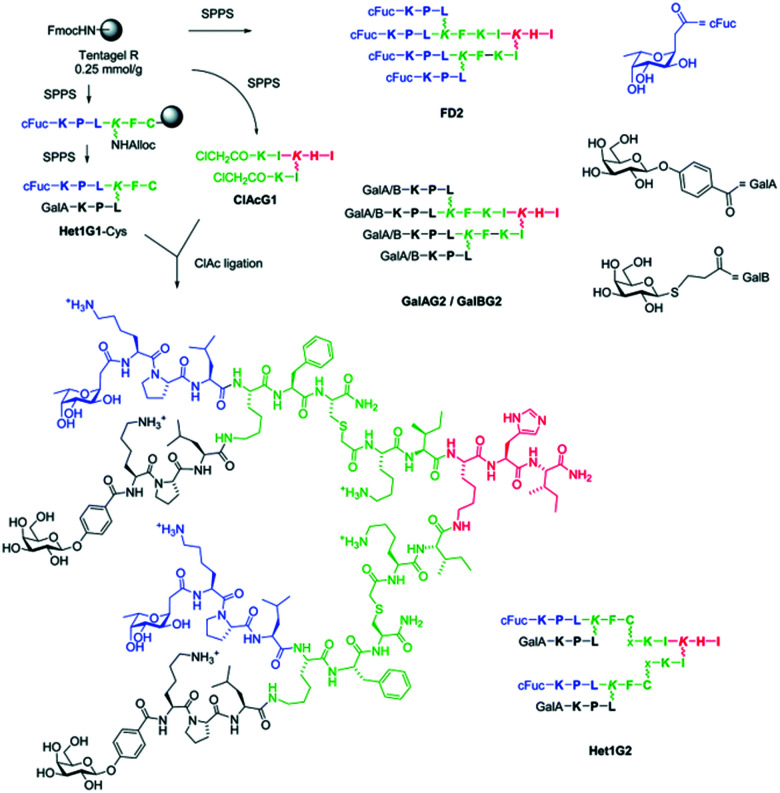
Structures of glycopeptide dendrimers FD2, GalAG2/GalBG2, and Het1G2. Figure reproduced from Reymond and co-workers with permission from The Royal Society of Chemistry, Copyright (2015).^378^

For example, glyco-AMPD GalAG2/GalBG2, FD2, and Het1G2 were developed to specifically target two lectins from *P. aeruginosa*, namely LecA, which normally binds galactosides, and LecB, which normally binds fucosides. LecA-specific galactosyl groups and LecB-specific fucosyl groups were attached to the N-terminus of the PD using a convergent approach of chloroacetyl (ClAc)-ligation to target *P. aeruginosa* biofilms (Fig. 16).^378,390,391^ The biological activity of the glycopeptide dendrimer was critically dependent on the multivalency and the nature of the aa sequence. This approach has enabled the SAR study of a tetrafucosylated PD FD2, where the authors demonstrated that this glycosylated PD was the most potent non-bactericidal biofilm inhibitor and dispersal agent among other glycopeptide dendrimers tested (GalAG2, GalBG2, and Het1G2). Additionally, synergistic effects were observed between sub-inhibitory concentrations of FD2 and tobramycin against *P. aeruginosa* biofilms, suggesting glycopeptide dendrimers could be suitable for use in drug combinations.^378^

**Fig. 17:**
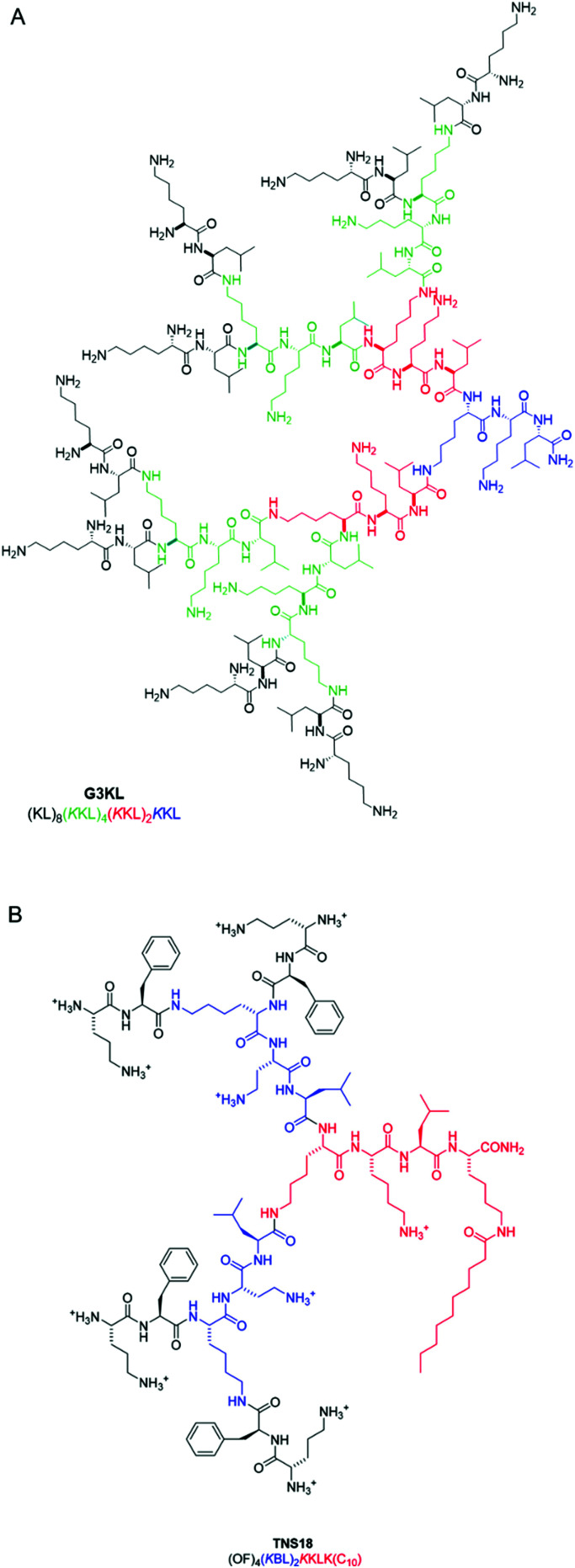
Structures of the peptide dendrimers G3KL^392^ (A) and TNS18^379^ (B). (A) Structure reported by Reymond and co-workers. (B) was adapted from Reymond and co-workers with permission from The American Chemical Society, Copyright (2017).

Whilst optimising the AMPD sequence, the Reymond group also designed a series of AMPDs in which they identified a G3 peptide dendrimer known as G3KL with the sequence (H-KL)_8_(*K*KL)_4_(*K*KL)_2_*K*KL-NH_2_ (*K* = branching lysine). This AMPD was formed of a Lys-Leu dipeptide, with the lysine α- and ε-amines used as branching point (Fig. 17A). This compound showed very potent antimicrobial activity against the opportunistic Gram-negative pathogens *P. aeruginosa* (4 μg ml^−1^) and *A. baumannii* (8 μg ml^−1^), predominantly by a membranolytic mechanism of action. G3KL was shown to resist proteolytic degradation in human serum, due to its compact globular structure.^392,393^ It was further shown to be remarkably active against MDR strains of *P. aeruginosa* and *A. baumannii*, to exhibit *P. aeruginosa* antibiofilm activity, and to enhance wound healing.^394–396^ Titration of G3KL showed that at neutral pH, the N-terminal amino groups were uncharged suggesting that out of the 24 total amines, only 15–17 protonated amines were needed for efficient antibacterial activity. G3KL was further shown to complex LPS at low serum concentration and to be taken up by Gram-negative bacteria up to 10% of the bacterial weight, similar to polymyxin B and a cathelicidin derivative, PMAP-23.^393,397^ More recently, this PD was shown to have a low propensity to cause the development of resistance.^37^ Moreover, this compound displayed a synergistic effect with several small molecule antibiotics in killing *K. pneumoniae*, against which G3KL alone is not active.^398^ In another study, the authors further optimised G3KL by exploiting combinatorial chemistry, and ultimately identified TNS18 with the sequence (H-OF)_4_(*K*BL)_2_*K*KLK(C_10_)-NH_2_ (O = ornithine, *K* = branching lysine, B = diaminobutyric acid) as a potent G2 lipopeptide dendrimer with promising *in vivo* activity against MDR clinical isolates of *A. baumannii* and *E. coli* (Fig. 17B).^379^ In addition to a lipidic chain attached to the core of the peptide dendrimer, TNS18 also contains unnatural aa such as diaminobutyric acid in the first generation and ornithine in the second generation. TNS18 was further shown to inhibit *P. aeruginosa* biofilms.^396^ CD spectroscopy was undertaken showing that TNS18 can adopt an α-helix in 20% trifluoroethanol, a solvent that induces α-helix formation, which is indicative of the ability of the peptide in question to act as a membrane disruptor. However, modelling studies also showed that TNS18 did not require helicity for its antibacterial activity, but rather used an open–closed conformation.^379,392,399^

### 11.2 Antimicrobial polypeptides

**Fig. 18:**
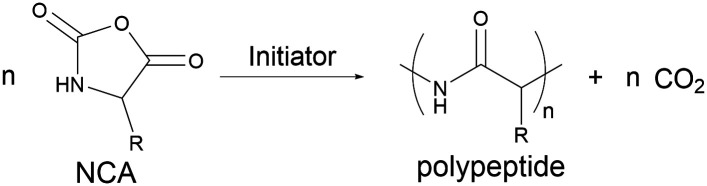
Synthetic scheme of synthetic polypeptides using NCA-ROP.

In addition to SPPS and recombinant techniques, peptides can be obtained *via* ring opening polymerisation (ROP) of *N*-carboxyanhydride (NCAs) monomers derived from α-aa. Initially developed in 1997 by Deming, this method is frequently used to yield high molecular weight peptides, typically referred to as polypeptides.^400^ Recent advances in polypeptide synthesis by ROP and the applications of polypeptides have very recently been summarised by Qiao and co-workers.^401^ NCA ROP is typically carried out in *N*,*N*-dimethyl formamide using a primary amine initiator (Fig. 18). The reaction can be controlled by cooling down the reaction mixture, using nitrogen flow, or by employing high vacuum methods developed by the Hadjichristidis group.^402–404^ Here, we will highlight a few polypeptides of different architectures that show remarkable activity against MDR bacteria and are therefore of great clinical interest.

**Fig. 19:**
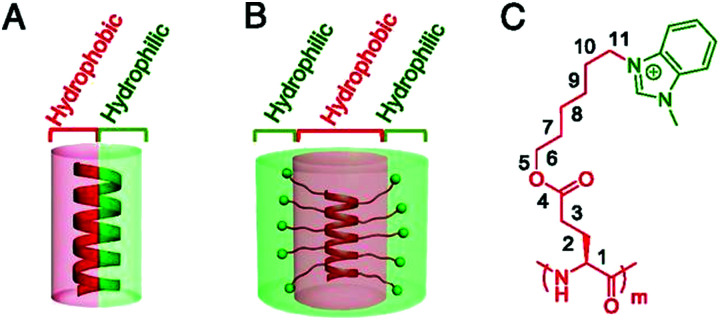
Schematic representation of facial (A) and radial (B) amphipathicity, and the chemical structure of PHLG-Blm family (C). Figure reproduced with permission from Xiong *et al.*, *Proc. Natl. Acad. Sci. U. S. A.*, 2015, **112**, 13155–13160.^405^

In 2015, Cheng and co-workers developed antimicrobial polypeptides, which display radial amphipathicity in contrast to the facial amphipathicity typically displayed by AMPs and were known as PHLG-Blm. This type of polypeptide was synthesised *via* ROP of γ-(6-chlorohexyl)-l-glutamate *N*-carboxyanhydride, followed by amination with 1-methylbenzimidazole to afford a positively charged hydrophilic terminus of the individual aa side chains (Fig. 19). This homo-polypeptide showed a helical conformation, as measured by CD spectroscopy, with a hydrophobic helical core and hydrophilic shell. The polypeptide showed strong activity against Gram-negative and Gram-positive bacteria, predominantly by membrane disruption. However, when the polypeptide was constructed using a mixture of d-, and l-aa, helicity was lost and the antibacterial efficacy was decreased; however, it displayed improved stability in serum and plasma.^405^

**Fig. 20:**
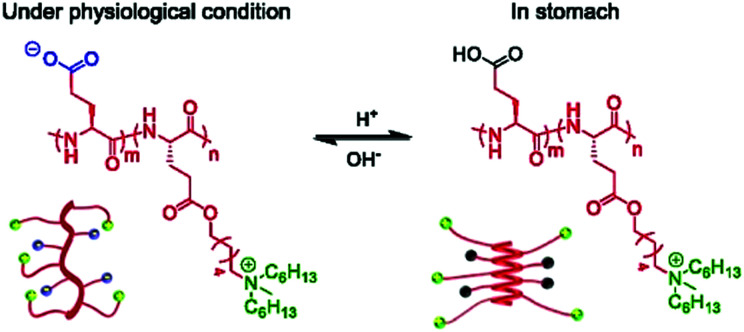
Schematic illustration of the pH-responsive PL2 polypeptide that transits to a helical conformation under acidic condition. Figure reproduced with permission from Xiong *et al.*, *Proc. Natl. Acad. Sci. U. S. A.*, 2017, **114**, 12675–12680.^406^

In 2017, the Cheng group designed pH-responsive polypeptides with a switchable helix-coil conformation. Random copolymerisation of 20-mer polypeptides was achieved by ROP of l-γ-(6-chlorohexyl)-Glu NCA and l-*tert*-butyl-Glu NCA followed by amination and trifluoroacetic acid-mediated ester hydrolysis. The polypeptides were designed such that upon protonation of the glutamic acid side chains, they would adopt an α-helical conformation that would effectively kill the stomach pathogen *Helicobacter pylori*. At physiological pH, it was expected that the helical structure would be distorted due to intramolecular electrostatic attraction between the anionic carboxylate and cationic amine functional groups, thus rendering the polypeptide inactive. In this manner, it was hoped that the polypeptide would only be active in the acidic environment of the stomach where *H. pylori* resides, thus reducing toxicity. *In vivo* studies of mice infected with *H. pylori* showed promising results for the polypeptide PL2 (Fig. 20), which exhibited low toxicity, low inflammation response activation, no significant injury to mucosa, while the mice maintained stable body weight and blood electrolytes. This pH-responsive polypeptide is an exemplary demonstration of a potent biocompatible antimicrobial polypeptide.^406^

**Fig. 21:**
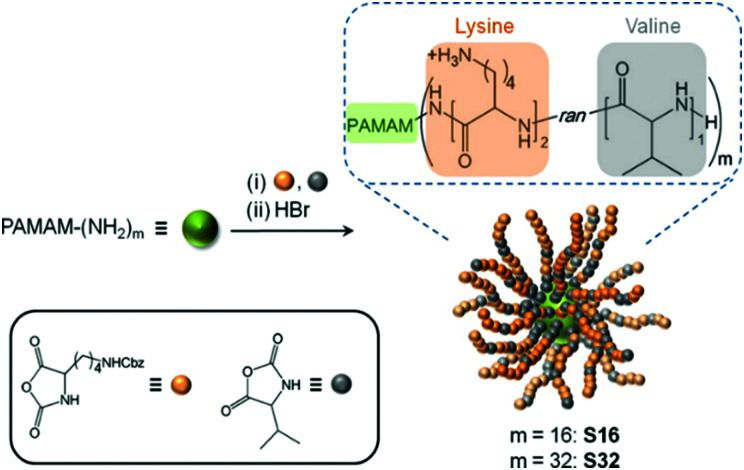
Schematic representation of SNAPPs with 16- and 32-arms. The synthesis was initiated from terminal amines of PAMAM dendrimers using ROP of lysine and valine NCAs. Figure reproduced from Qiao and co-workers with permission from The American Chemical Society, Copyright (2016).^408^

In 2016, Qiao and co-workers reported a new class of antimicrobial polypeptides known as structurally nanoengineered antimicrobial peptide polymers (SNAPPs). SNAPPs use second- and third-generation PAMAM dendrimers as a core unit onto which lysine and valine residues could be randomly incorporated *via* ROP of lysine and valine NCAs (Fig. 21). The resulting SNAPPs were referred to as ‘star-molecules’ with either 16- (43.8 kDa) or 32-arms (74.8 kDa), with 30 residues per arm and a lysine-to-valine ratio of 2 : 1. These star-molecules exhibited activity against ‘ESKAPE’ pathogens including *E. coli*, *P. aeruginosa*, *K. pneumoniae* and *A. baumannii* with sub-micromolar activity, preferentially acting as strong membrane disruptors. In addition, the study showed that resistance was not acquired after 600 generations at sub-minimum bactericidal concentrations (MBC) with *A. baumannii*. However, like many cationic AMPs, the activity of the SNAPPs against Gram-negative bacteria decreased due to divalent cations in simulated body fluid that mimics the *in vivo* ionic composition. Nevertheless, the study highlighted that SNAPPs can potentially be used as novel antimicrobial agents against *A. baumannii* infections since they remained active in high salt concentrations and in the presence of serum proteins.^407–409^

**Fig. 22:**
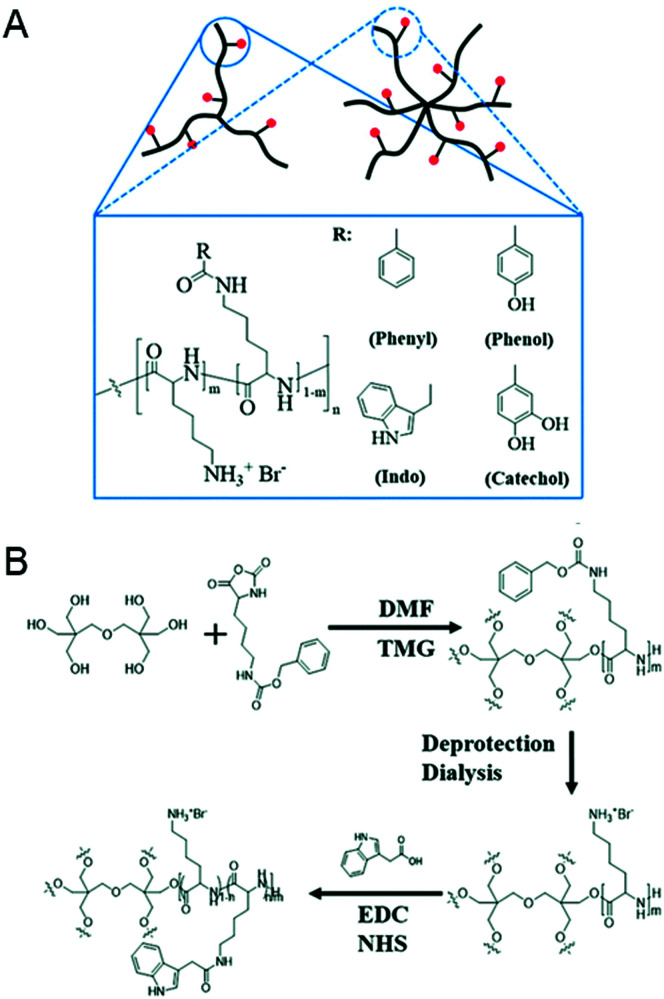
(A) Schematic representation and chemical structure of star-shaped polypeptides bearing different pendent groups. (B) Synthesis scheme of star-shaped co-polypeptide with 6 arms using dipentaerythritol as the initiator. Figure reproduced from Jan and co-workers with permission from The Royal Society of Chemistry, Copyright (2019).^410^

Recently, Jan and co-workers identified amphipathic star-shaped molecules with effective antimicrobial activity. Dipentaerythritol was used as an initiator, which forms the core of the structure from which 6-arms could then be extended. This was achieved by using ROP of *Z*-l-lysine NCA, followed by the removal of the protecting group on the polypeptide using HBr (Fig. 22). Hydrophobic groups such as indoleacetic acid were then attached to the lysine side chain using 1-ethyl-3-(3-dimethylaminopropyl)carbodiimide or *N*-hydroxysuccinimide. One advantage of this synthetic strategy is that the hydrophilic and hydrophobic moieties of the polypeptides can be more easily controlled than in typical polymerisation reactions. The resulting star-shaped polypeptides showed potent antibacterial activity against Gram-negative bacteria, no toxicity to human cells, and a low haemolysis rate. Additionally, these polypeptides were able to reduce inflammatory cytokines IL-6 and TNF-α in mice infected with enterohaemorrhagic *E. coli*. This study showed that the architecture of the polypeptides and their amphipathic nature are both important factors for their antimicrobial activity.^410^

More recently, Kim and co-workers reported the rapid and large-scale synthesis of topologically nanoengineered antimicrobial polypeptides (TNAPs) *via* a metal-free imidazolium hydrogen carbonate-mediated NCA polymerisation.^411,412^ With this synthetic strategy the authors were able to develop amphipathic linear- (l), hinged- (h), star- (s), and cyclic- (c) polypeptides composed randomly of poly(l-lysine) and poly(γ-benzyl-l-glutamate). The helicity of the polypeptides increased with increasing ratio of benzyl glutamate. Comparing polypeptides with the same Lys-to-benzylGlu ratio, but different structural architectures, the antibacterial activity was as follows: l- < c- < h- < s-TNAP. This study has emphasised the impact of polypeptide architecture on antibacterial activity.^412^

## 12 Random peptide cocktails as a strategy to combat MDR bacteria

The molecular diversity of AMPs suggests that their selective activity against bacteria is closely related to broad physicochemical properties such as net positive charge and amphipathicity (see Section 4). Upon binding to anionic bacterial membranes, cationic AMPs typically adopt an α-helical conformation such that the hydrophobic moiety can insert into the membrane, thus causing a membrane disruption (see Section 6.1). The unique physicochemical properties of AMPs have inspired the development of various synthetic cationic–hydrophobic random copolymers that mimic the antimicrobial activity of AMPs, the discussion of which is outside the scope of this review. However, we refer the reader to a recent review from Kuroda and co-workers for a detailed description of this fascinating area of research.^413^

**Fig. 23:**
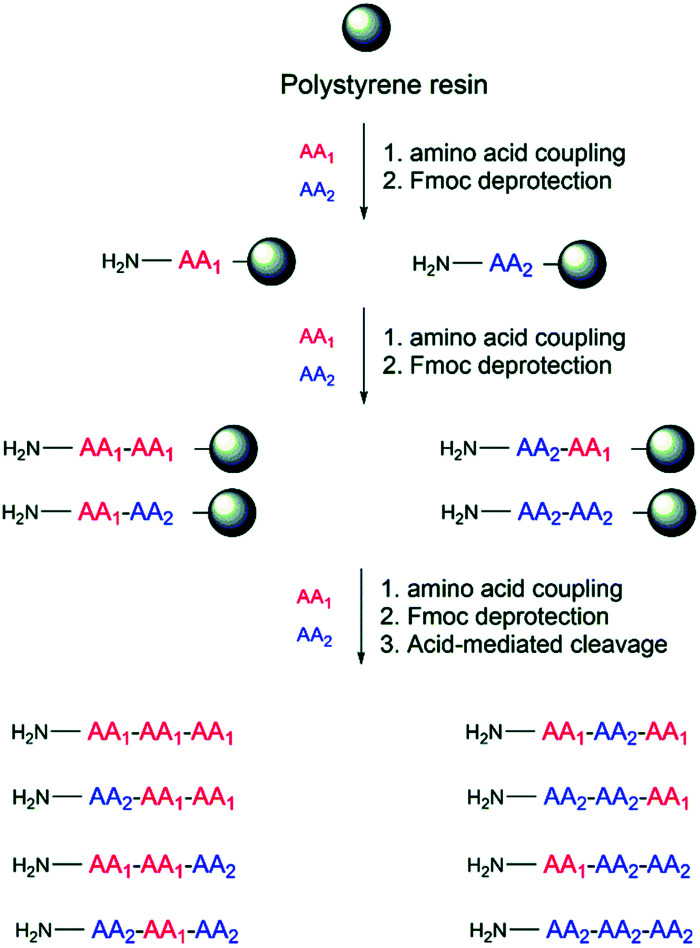
Schematic representation of the synthesis of random peptide cocktails *via* SPPS. Three coupling steps are shown in the figure. A mixture of aa is added at each coupling step to yield peptides composed of several different sequences with the same length. Figure reproduced from Hayouka and co-workers with permission from The Royal Society of Chemistry, Copyright (2019).^417^

Based on the methodology developed for the synthesis of random copolymers, Gellman and co-workers generated sequence-random poly(α-aa) libraries by SPPS, using both l- and d-aa. The poly(α-aa) mixtures were first synthesised in a homochiral manner *i.e.* solely with l- or d-α-aa, and then in a heterochiral manner *i.e.* a mixture of l- and d-α-aa.^414^ At each coupling step, a mixture of aa was added to the resin, ultimately affording a library of peptides of the same length but different sequences (Fig. 23). The first libraries were composed of 20-mer peptides, with each library containing peptides comprised of only two l-α-aa: one hydrophobic and one cationic *e.g.* L/K, I/K, F/K, L/R, I/R or F/R. None of these homochiral binary peptide mixtures displayed simultaneous potent antibacterial activity and low haemolytic activity, despite control over the peptide chain length, aa identity, and aa proportion. Selecting the most promising subunit identity, subunit proportion, and chain length parameters identified in the l-aa peptides, the group then sought to investigate the effects of stereochemical variation on these properties by using various combinations of l- and d-aa. This series of heterochiral mixed peptides maintained the antibacterial activity of the homochiral peptide mixtures, and exhibited a significant reduction in the haemolytic activity.^414^

Hayouka and co-workers later showed that heterochiral peptide mixtures were able to selectively attack bacterial membranes without the formation of visible pores.^415^ To this end, they constructed two libraries with sequences containing only Phe and Lys, but with different Lys chirality. The final libraries contained a cocktail of 2^20^ random peptides. During the synthesis, a pre-defined ratio of Phe and Lys was added at each coupling step, leading to a random peptide sequence, but well defined peptide length and stereochemistry. The heterochiral (Fk) mixtures showed strong disruption of MRSA biofilm biomass compared to the homochiral mixtures (FK).^415^ The researchers also showed that the homochiral and heterochiral peptides operate by different mechanisms of action. The homochiral peptides were able to cooperatively assemble into anionic phospholipid bilayers in a manner similar to that of sequence-defined AMPs, whereas the heterochiral peptides inserted into membrane bilayers through random oligomerisation. The heterochiral peptide sequences showed minor membrane disruption, suggesting an intracellular mechanism of action.^416,417^

Random peptide mixtures of l-Lys and l-Leu have been shown to inhibit Gram-positive and Gram-negative bacteria found in pasteurised bovine milk, highlighting their ability to target different bacterial membranes and thus prevent the growth of a broad spectrum of bacteria. These peptide mixtures have therefore been proposed as potential food preservatives.^417–419^ One important advantage of random peptide mixtures resides in the fact that purification is not required, rendering the synthesis low-cost.

Using a similar approach, the Reymond group has recently demonstrated that SPPS using racemic aa followed by high performance liquid chromatography (HPLC) purification can yield stereorandomised peptides that contain up to billions of stereoisomers, which can be used to probe the biological activity of peptides. For example, the researchers showed that stereorandomisation of α-helical amphiphilic membrane disruptive peptides, including D-JK-5 (see Section 9.1) and indolicidin, preserves their antibiofilm effect, despite the disordered conformations. In contrast, stereorandomised AMPDs, including G3KL (see Section 11.1), keep their antibacterial, membrane-disruptive, and antibiofilm effects with reduced hemolysis and cytotoxicity. Moreover, the authors also showed that partial stereorandomisation of polymyxin B analogues preserved the antibacterial activity but caused a loss of its membrane-disruptive and LPS-neutralising activity, suggesting that these analogues killed the Gram-negative bacteria by another mechanism of action, perhaps involving other targets.^420^

We have shown in this section that a poly(α-aa) mixture of homochiral peptides, or peptides with a mixed chirality, can be a promising strategy to develop novel AMPs with new targets, and to potentially prevent the development of AMR.

## 13 Computer-aided design of AMPs

### 13.1 Artificial intelligence

Artificial intelligence (AI) has gained importance in the field of science and medicine due to the advancement of computer power, the availability of large amounts of data, publicly available neural networks, and improvements in AI algorithms such as machine learning and deep learning.^275^ Machine learning presents a smart and efficient method for optimising AMP sequences, by learning from extensive and comprehensive training data.^421^ For this purpose, various algorithms have been developed based on machine learning methods, such as support vector machine (SVM), fuzzy K-nearest neighbour (KNN), random forest (RF), and neural network (NN).^275,421^ We refer the reader to a detailed description of each of the methods applied to the area of AMPs in recent reviews written by Franco and co-workers and Wong and co-workers.^421,422^

Among the different machine learning strategies, the quantitative structure–activity relationship (QSAR) model is the most broadly used. This model uses physicochemical descriptors in order to predict the biological activity of peptides from their aa sequence.^423,424^ We refer the reader to a mini-review of recent trends in QSAR-based virtual screening in drug discovery by Andrade and co-workers.^425^

Ding and co-workers used a QSAR-based model to reveal that site-directed substitution of hydrophobic aa with less lipophilic residues in amphipathic peptides can decrease their haemolytic activity without significantly affecting the antimicrobial activity. Furthermore, cyclisation of the potent linear peptides *via* disulfide bond formation between two cysteine residues placed at the N- and C-termini resulted in more potent antimicrobial activity and better proteolytic stability. These disulfide-bridged cyclic peptides showed high activity at sub-nanomolar concentrations against several Gram-negative pathogens including *E. coli*, *P. aeruginosa*, and *K. pneumoniae*, while also displaying low haemolytic activity and low toxicity to human monocytes.^426^ Barron and co-workers reported a QSAR-based model that was able to accurately predict peptoid antibacterial activity by analysing a set of structurally diverse peptoids.^427^ Furthermore, Jerala and co-workers have demonstrated the use of a QSAR-based model to identify motifs in coiled-coil forming peptides that were capable of forming silver nanoparticles.^428^

In order to optimise a linear AMP sequence, the Hancock group performed *in silico* screening of 3D structures of peptides from a virtual library using a QSAR-based model to predict antibiofilm activity. In this study, the QSAR modelling approach successfully classified the peptides from the virtual library with 85% prediction accuracy, and ultimately enabled the identification of peptide 3002 (sequence H-ILVRWIRWRIQW-NH_2_) that showed 8-fold higher efficiency in eradicating an established MRSA biofilm *in vitro* compared to the peptide 1018, a 12-mer derived from bactenecin.^429,430^ Furthermore, 3002 successfully reduced the size of an abscess in a chronic MRSA mouse infection model.^430^ Although this method has been demonstrated as a promising strategy for the computer-aided design of improved antibiofilm peptides, the authors anticipate more accurate predictions can be performed by iterative improvement of the QSAR models as the number of active sequences deposited in the databases increases.^430^ Tossi and co-workers applied “Mutator”, a freely available web-based computational tool for suggesting residue modifications that can potentially increase the selectivity of AMPs based on QSAR criteria, to a large set of peptides in an anuran AMP/activity database.^431–435^ This approach has successfully led to the identification of Dadapin-1, which showed potent activity against *S. aureus* and moderate activity against Gram-negative bacteria such as *P. aeruginosa*, *A. baumannii*, *E.coli*, and *K. pneumoniae*.^436^ More recently, Idicula-Thomas and co-workers used a QSAR-based model to accurately predict the activity of rationally designed peptides derived from the cathelicidin AMP family against *E. coli* ATCC 25922.^437^

Apart from AI and machine learning, other computational methods have demonstrated their efficacy to predict and identify potent antimicrobial peptides.^421^ Methods including *de novo* (non-template sequence), linguistic, pattern insertion, and evolutionary/genetic algorithms in the area of AMPs have been summarised recently.^421^

### 13.2 Chemical space as an AMP source

Chemical space is a cheminformatic concept that refers to a property space comprising all the possible small organic molecules, including those that exist in biological systems. Various algorithms can be applied to explore defined regions of chemical space and generate focused virtual libraries.^438^ The process of synthesising and biologically testing hits identified by virtual screening of chemical space has revealed a very useful approach for identifying new potent AMPs. For instance, the Reymond group have recently exploited chemical space using atom-pair fingerprint similarity searching to discover and optimise antimicrobial bicyclic peptides and peptide dendrimers against MDR Gram-negative bacteria.^399,439,440^

Bicyclic peptides are interesting scaffolds due to their highly constrained conformation significantly limiting the rate of proteolytic degradation. Various strategies for the chemical synthesis of bicyclic peptides have been summarised in a mini-review by Pei and co-workers.^441^ In 2019, Bicycle Therapeutics entered into a partnership with the Department of Health and Social Care in Cambridge, UK, as part of the Small Business Research Initiative (SBRI) to identify bicyclic (Bicycle®) peptide inhibitors that target Penicillin Binding Proteins (PBPs).^442^

**Fig. 24:**
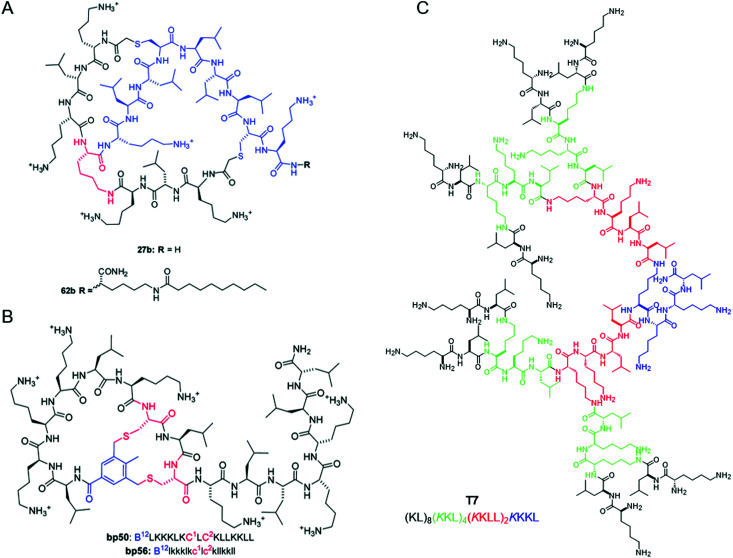
Hit bicyclic peptides 27b and 62b (A), bp50 and bp56 (B), and peptide dendrimer T7 (C) identified from virtual libraries generated from the chemical space. (A) adapted from Reymond and co-workers with permission from The Royal Society of Chemistry, Copyright (2017).^439^ (B) adapted from Reymond and co-workers with permission from The Royal Society of Chemistry, Copyright (2018).^440^ (C) Structure reported by Reymond and co-workers.^399^

To identify new antimicrobial bicyclic peptides, Reymond and co-workers took advantage of the chemical space that encoded molecular shapes and pharmacophores to describe a virtual library of bicyclic peptides and explore their diversity first using a small number of test compounds and later optimising the initial hits by focusing on the nearest neighbours. The bicyclic peptide library was synthesised by SPPS and assembled with a double thioether bridge connecting two cysteines and the chloroacetylated N-terminus, leading to 27b and its lipidated analog 62b (Fig. 24A), which confer good antimicrobial activity against Gram-negative *P. aeruginosa* and several other MDR clinical isolates.^439^

In a second study of antimicrobial bicyclic peptides, 4 685 090 bicyclic peptides were enumerated in a virtual library covering a broad size range, featuring linear peptide tails of varying length, and exploring different distributions of leucine and lysine in the cyclic and acyclic portions of the peptide sequence. The bicyclic peptide library was clustered in atom-pair 2D-fingerprints to select 31 bicyclic peptides for synthesis. One analogue, bp50, was identified and the linear sequence of l-aa, as well as its d-enantiomer bp56, were synthesised by SPPS (Fig. 24B). Subsequent double intramolecular thioether ligation between two cysteine residues inserted into the linear sequence and a 3,5-bis(chloromethly)toluoyl group at the N-terminus afforded the desired bicycle. Both bp50 and bp56 were shown to be efficient at killing MDR strains of *A. baumannii* and *P. aeruginosa*. However, bp56 exhibited much better stability in human serum than bp50.^440^

Using a similar computational approach, the Reymond group recently improved the activity of the antimicrobial peptide dendrimer G3KL (see Section 11.1). The hit peptide dendrimer, known as T7 with the sequence (H-KL)_8_(*K*KL)_4_(*K*KLL)_2_*K*KKL-NH_2_ (*K* = branching lysine), was identified from a virtual library of 50 625 dendrimers, which contained all of the possible permutations of up to three residues of Lys or Leu in the branches and using Lys as branching diamino acid (Fig. 24C). This peptide dendrimer displayed high efficacy against a panel of Gram-negative bacteria, including MDR clinical isolates of *P. aeruginosa*, as well as *K. pneumoniae* strains, against which the reference peptide dendrimer G3KL was inactive.^399^

## 14 Antimicrobial peptidomimetics (AMPMs)

Peptidomimetics are molecules that mimic natural peptide structures, or that mimic the biological effect of natural peptides and bear no resemblance to their structure.^443^ Antimicrobial peptidomimetics (AMPMs) can be designed based on the sequence of a naturally occurring parent AMP, or so as to contain general structural features and properties that are known to promote antimicrobial activity. Some AMPMs are very similar to natural AMPs, largely maintaining the peptide backbone but introducing a limited number of aa substitutions in order to promote the bioactive conformation of the peptide.^444–446^ At the other extreme, they may be small molecules that mimic the AMP mechanism of action but show no resemblance to natural AMP structures.^447^ Often, mimetics are designed to exhibit key physicochemical properties of natural AMPs, such as net positive charge and facial amphipathicity. By imparting a mimetic with these properties, it is hoped that they will operate by the same mechanism of action, which is thought to have a reduced propensity for bacterial resistance. This is evidenced by AMPs retaining antimicrobial activity despite having been a key component of host-defence mechanisms for thousands of years.

One advantage of peptidomimetics over typical α-peptides (*i.e.* peptides made exclusively from α-aa) is that they can be structurally simpler, and therefore cheaper and easier to chemically synthesise. Additionally, peptidomimetics can display improved antimicrobial activity, improved stability (both metabolic and proteolytic), and reduced toxicity compared to unmodified α-peptides.^448^

Here, we will highlight a few illustrative examples of different classes of AMPMs to give the reader an appreciation of the diversity of peptidomimetic structures that have been described in the literature. However, this is far from an exhaustive list of examples and we direct the reader to several recent reviews for a more in-depth discussion.^445–448^

### 14.1 Stapled AMPs (STAMPs)

**Fig. 25:**
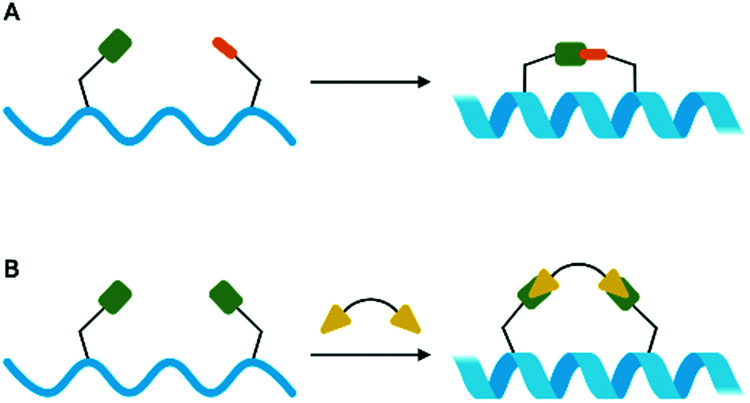
The two approaches to peptide stapling: one-component (A) and two-component (B).

Stapled AMPs (STAMPs) are a class of macrocyclised AMPs wherein cyclisation has occurred between two side chain residues and can be performed in a one-component, or a two-component manner (Fig. 25).^449^ One-component peptide stapling consists of an intramolecular reaction between two aa side chains, whereas two-component stapling involves an initial reaction between one of the side chain residues and a bi-functional linker, followed by the reaction of a second side chain residue and linker, resulting in macrocyclisation. Both methodologies can make use of either natural or unnatural aa for the stapling reaction, which can be performed either on- or off-resin, depending on the methodology employed.

Stapling is typically used to enforce an α-helical conformation in peptides where this is known to be the bioactive conformation. However, it is also possible to stabilise other conformations through stapling.^450,451^ Enforcing the bioactive conformation of a peptide is desirable as it can lead to improved binding affinity by reducing the entropic cost of binding. Furthermore, stapling can improve proteolytic stability by restricting access of proteases to the peptide backbone. AMPs must survive both eukaryotic and prokaryotic proteases before reaching the intended target; therefore, stapling can drastically increase their plasma half-life. Stapling can directly alter the properties of AMPs, but some stapling methodologies also provide a chemical handle through which further functionality can be appended.^452–454^

Reports of STAMPs (and of stapled peptides in general) are dominated by one technique: ring-closing metathesis (RCM). This involves introducing unnatural aa residues with alkene-bearing side chains into the peptide sequence, which are then chemically linked on-resin by RCM using Grubbs catalysts.^455^ Kamysz and co-workers recently reviewed RCM- (or ‘hydrocarbon-’) STAMPs, and therefore we direct the reader to their 2018 review for a more in-depth discussion.^452^

Hydrocarbon stapling has been applied both to AMPs that have been designed as analogues of natural AMPs, and to sequences that have been designed to mimic typical AMP characteristics such as being amphipathic with a net positive charge.^456–458^ Additionally, there are examples of STAMPs containing multiple staple linkages (‘stitched peptides’).^459,460^ Although hydrocarbon STAMPs often display improved proteolytic stability and α-helicity relative to the linear peptide, this stapling can also sometimes result in higher levels of haemolysis and lowered antibacterial activity.^452^ In these scenarios, often the nature and placement of the staple, as well as the peptide sequence, can all be adjusted to overcome these challenges while maintaining the general benefits of stapling.

The number of possible permutations creates a potential minefield for researchers attempting to develop new STAMPs. To address this issue and establish basic design guidelines for RCM stapling of natural peptide sequences, Walensky and co-workers performed a systematic screen using magainin II as a model peptide.^461^ The authors produced a library of magainin II analogues by varying the position of an *i*, *i* + 4 hydrocarbon staple, created hydrophobic network maps for each peptide and assessed the MIC of each STAMP against various bacterial strains, as well as the degree of haemolytic activity. A key finding was that the staple must be incorporated into a pre-existing region of hydrophobicity to avoid undesirable haemolysis, and with this information, the authors optimised a stapled magainin II peptide and confirmed a membrane-lytic mechanism of action. Following on from this work, the group have filed a patent for all-hydrocarbon stapled AMPs based on the sequence of magainin II.^462^ Using the design guidelines established during the development of this magainin II STAMP, the group also developed an algorithm for STAMP design, and were able to rapidly design STAMPs based on the sequences of pleurocidin, CAP18, and esculentin.^461^ The resulting STAMPs displayed potent antibacterial activity and negligible haemolysis without the need for an extensive and systematic library screening approach for each AMP.

Although hydrocarbon stapling is considered the gold standard of peptide stapling, alternative one- and two-component methodologies do exist and have been used for creating STAMPs.^426^ An alternative one component approach includes disulfide or amide bond formation, and two-component techniques include S_N_Ar reactions of Cys residues and perfluoroaryl electrophiles, thioether formation, and alkylation of Lys residues.^426,463–465^

### 14.2 Non-typical amino acid AMPMs

**Fig. 26:**
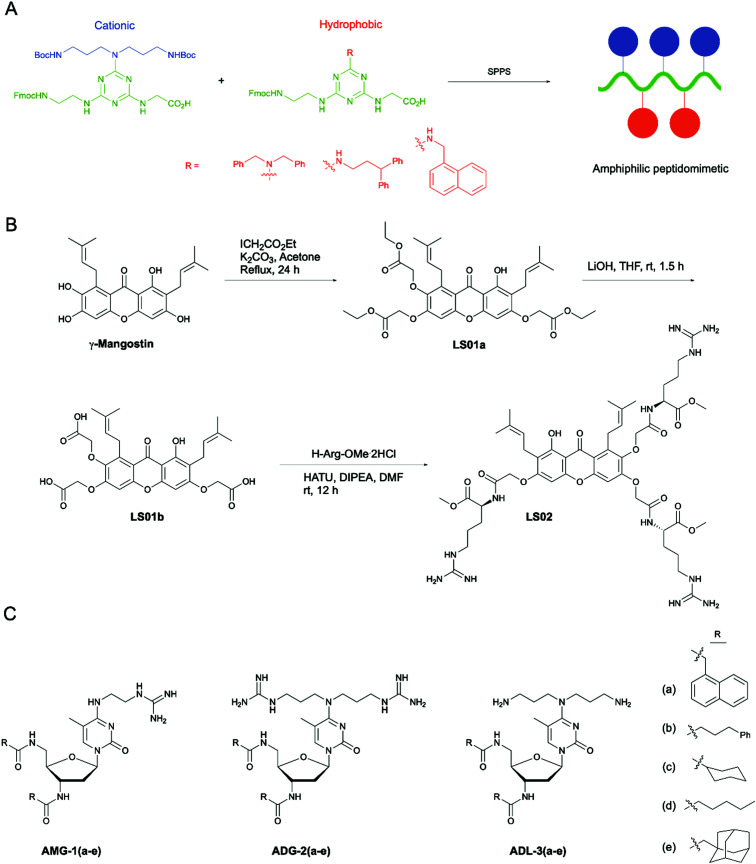
(A) Fmoc-triazine aa used to make AMPMs by Bang and co-workers.^474^ (B) γ-Mangostin AMPM derivative LS02. Figure adapted from Lin *et al.*, *Biochim. Biophys. Acta - Biomembr.*, 2020, **1862**, 1–10, with permission from Elsevier, Copyright (2020).^475^ (C) The structure of azidothymidine (AZT)-based small molecule antimicrobials adapted from Chirumarry *et al.*, *Eur. J. Med. Chem.*, 2020, **193**, 1–13, with permission from Elsevier, Copyright (2020).^476^

A common strategy for generating AMPMs is to incorporate one or more ‘non-typical’ aa in the sequence. We use the phrase ‘non-typical’ to encompass a broad range of aa that are not generally found in naturally occurring AMPs. These non-typical aa can include unnatural α-aa, β-aa, γ-aa, and N-substituted aa (yielding peptoids).^466–471^ In more extreme examples, the typical peptide backbone is almost unrecognisable. For example, Bang and co-workers developed a series of AMPMs using a triazine scaffold as their repeating unit (Fig. 26A).^466,470,472–474^ Each 1,3,5-triazine core was substituted with an Fmoc-protected amine, an acid, and either a cationic or hydrophobic group, generating Fmoc-triazine aa building blocks. Using Fmoc-SPPS, these building blocks were then combined with cationic and hydrophobic residues in alternating positions, ultimately affording amphipathic peptidomimetic structures. All these non-typical aa peptidomimetics are amenable to Fmoc-SPPS.

### 14.3 Small molecule AMPMs

There are some small molecules that mimic the mechanism of action of AMPs (‘mechanistic AMPMs’). These scaffolds are not necessarily designed and can be identified through library screens, or they may also derive from extensive optimisation of other mimetics that bear a closer resemblance to natural AMPs. For example, natural xanthone compounds such as α- and γ-mangostin disrupt cytoplasmic membranes in a similar mechanism of action to natural AMPs. While these mangostins may be considered mechanistic AMPMs, their structure bears no resemblance to a natural peptide, nor were they designed to be as they are natural products themselves. Liu and co-workers recently reported a γ-mangostin derivative, LS02, with excellent activity against Gram-positive bacteria, while also exhibiting good water solubility and low haemolytic activity, in contrast to the parent compound (Fig. 26B).^475^

Bang and co-workers also recently reported a series of azidothymidine (AZT)-based small molecules that incorporate both cationic and hydrophobic groups in order to mimic the amphipathic structure, and therefore mechanism of action, of AMPs (Fig. 26C).^476^

### 14.4 Peptidomimetic hybrids

The term ‘hybrid’ is sometimes used to describe peptidomimetics that contain various types of backbone in the same structure, such as α-peptide/β-peptide, α-peptide/α-peptoid, α-peptide/β-peptoid or α-peptide/γ-aa peptide combinations.^448,477^ However, there are a few key examples where two separately designed and established peptidomimetics were joined in a way similar to hybrid peptides (see Section 9.7), affording a true hybrid peptidomimetic structure.

**Fig. 27:**
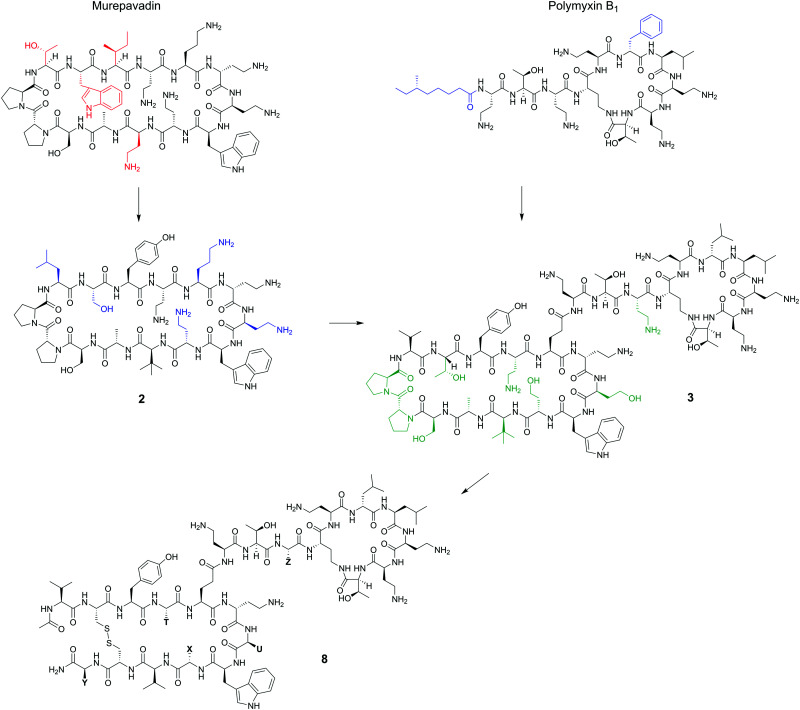
Synthesis of peptidomimetic hybrids. Murepavadin derivative 2 was prepared by substitution of several aa residues (in red). The derivative 2 was further optimised and a hybrid with a polymyxin B_1_ analogue prepared (substituted residues in blue). The resulting hybrid 3 was further optimised to hybrid 8 (green residues modified), which contains 5 variable residues (*T*, *U*, *X*, *Y*, *Z*) that were not specified by the authors.^479^

A joint effort between industry and academia produced a series of peptidomimetic hybrids based on murepavadin and polymyxin B (murepavadin itself being a β-turn peptidomimetic of protegrin I) shown in Fig. 27.^241,478^ Obrech, Robinson and co-workers produced peptidomimetics of murepavadin and then formed a hybrid between a derivative of one of the peptidomimetics (2) and a section of polymyxin B. This combination was chosen to extend the mode of action of murepavadin, which interacts with outer membrane proteins of Gram-negative bacteria, by adding a further interaction with lipid A through the polymyxin moiety. One of the most potent chimeras (3) was further engineered to afford 8, which has been advanced to pre-clinical studies by Polyphor Ltd under the name POL7306.^479^ Analogue 8, 5 residues of which were not disclosed by the authors, and parent 3 showed broad spectrum antibacterial activity, low mammalian cytotoxicity, and *in vivo* activity in a mouse model of peritonitis (*E. coli* and *K. pneumoniae*) and thigh infection (*E. coli*, *P. aeruginosa*, and *A. baumannii*). In a separate study, 8 displayed activity against an extensive panel of MDR strains.^479^ Several peptidomimetics with functionalised unnatural aa were also synthesised and used for confirmation of the mechanism of action. All of the peptidomimetics were synthesised by SPPS.

## 15 Conjugation strategies

Given the unusual and distinct properties and mechanisms of action of AMPs (and AMPMs), a rapidly growing area of research investigates their conjugation to other chemicals (or ‘cargo’), for example small molecule antibiotics and polymers. The benefits of creating peptide–drug conjugates (PDCs) such as these include the creation of a species with multiple mechanisms of action (with the potential for a ‘synergistic-like’ effect), improving the properties of the cargo or peptide, and allowing targeted delivery of the cargo, therefore reducing the likelihood of undesired side effects. There are numerous examples of AMP conjugates in the literature, and we direct the reader to the excellent review by Gray and Wenzel.^131^ A non-exhaustive list is given in Table 5, highlighting the structural and functional diversity within this class of antimicrobial agents, as well as the different synthetic strategies used for their construction. The synthetic approaches to AMP conjugates typically involve Fmoc-SPPS of the peptide (or peptidomimetic), often with the incorporation of reactive aa residues (either unnatural or canonical) at either the N- or C-terminus. The cargo is then modified with a reactive linker, enabling it to be coupled to the peptide, either in solution, or while the peptide is attached to the solid support.

**Table 5:** A non-exhaustive list of PDCs

Drug/warhead (antibiotic class)	AMPM/AMP/CPP (specific example)	Nature of conjugation (synthetic strategy)	Ref.
Fluoroquinolones	Peptidomimetic CPP	Non-cleavable (peptide modified with fluoroquinolone on solid-support)	480
Tobramycin (aminoglycoside)	CPP (penetratin)	Non-cleavable (cargo conjugated to aa *via* CuAAC then incorporated during SPPS)	481
Levofloxacin (fluoroquinolone)	Tetra-branched AMP (M33)	Non-cleavable (levofloxacin coupled to ε-amine of Lys on solid-support)	482
Chloramphenicol	AMP (UBI_29-41_)	Non-cleavable (chloramphenicol coupled to N-terminus of peptide in solution)	483
Ceftazidime (cephalosporin)	Glycopeptide (vancomycin)	Non-cleavable (solution-phase coupling)	484
Methotrexate	Two peptides: cationic delivery AMPM and anionic poly-Glu peptide	Cleavable (β-lactamase sensitive cephalosporin moiety linker between the peptides)	485
Kanamycin (aminoglycoside)	AMPM	Cleavable (AMPM N-terminus modified with disulfide-containing linker, cargo attached on resin)	486
Ciprofloxacin (fluoroquinolone)	AMP (HLopt2)	Cleavable (linked *via* disulfide bond formation at Cys residue of AMP)	487
Chitosan	AMP (Jelleine I)	Cleavable (linked *via* disulfide bond formation at Cys residue of AMP)	488

For many drugs, even minor structural alterations can drastically alter the biological activity. Therefore, it is easy to imagine how the attachment of a chemical linker and large peptide to an antibiotic may negatively impact the antimicrobial effect. A more elegant approach could involve the use of a cleavable motif that releases the free, unmodified drug at the desired site of action. Examples include linkers between the peptide and drug that feature disulfide bonds, or enzyme-cleavable motifs. The addition of a cleavable linker requires thought to be given to the nature of the trigger, the sensitivity of the cleavable motif, the kinetics of release and may add synthetic challenges.

However, there are many possible advantages to cleavable linkers, which will be discussed later. In this section, we will discuss a few recent, illustrative examples of both non-cleavable and cleavable PDCs, emphasising the synthetic strategies used to construct each PDC.

### 15.1 Non-cleavable PDCs

#### 15.1.1 AMP–Drug conjugates

In an antimicrobial PDC, the AMP typically serves to provide an additional and alternative mechanism of action to the drug. A membrane-disrupting AMP both exerts an antimicrobial effect and increases the uptake of the traditional drug component of the PDC. There are examples in the literature of PDCs containing a variety of different AMPs and small-molecule antimicrobial agents, each constructed using a slightly different synthetic approach. The antimicrobial activities and successes of these PDCs depend greatly on their composition, and the bacteria in question. Several classes of antibiotics are widely employed in conjugation strategies, including fluoroquinolones, cephalosporins, vancomycin and aminoglycosides. Typically, these antibiotics are tolerant of chemical modification, allowing their linkage to other species.

For example, two different groups have synthesised PDCs of vancomycin (‘vanco’), one containing cathelicidin-related AMP (CRAMP, sequence H-KIGEKLKKIGQKIKNFFQKLVPQPEQ-NH_2_) as the peptide component, and one containing Hecate (‘Hec’, sequence H-FALALKALKKALKKLKKALKKAL-OH), an AMP derived from melittin.^489,490^ The goal of the CRAMP-Vanco PDC was to broaden the spectrum of activity displayed by vancomycin. To synthesise the PDCs, the CRAMP was synthesised *via* Fmoc-SPPS, and the N-terminus was capped with 4-azido-butanoic acid, enabling it to be conjugated to an alkyne-containing vancomycin derivative *via* a CuAAC reaction. Different linkers were investigated, and PDCs were identified that gave good antimicrobial activity against Gram-negative species and retained similar activity to vancomycin alone against Gram-positive species. These conjugates were also more effective than either vancomycin or CRAMP alone in disrupting the biofilms of *S. typhimurium*, a Gram-negative bacterium. The Hec-Van PDC was synthesised in an attempt to regain activity against vancomycin-resistant bacterial strains. The conjugate was prepared by simple solution-phase 1,1′-carbonyldiimidazole-mediated amide coupling of the vancomycin C-terminal carboxylate and Hecate N-terminal α-amino group. The resulting PDC displayed improved MICs against *S. aureus*, MRSA, and VRSA, and reduced haemolysis compared to the peptide alone. Additionally, when *E. coli*, a Gram-negative strain, were treated with the conjugate at 10 μM, over 97% of the cells were killed.

**Fig. 28:**
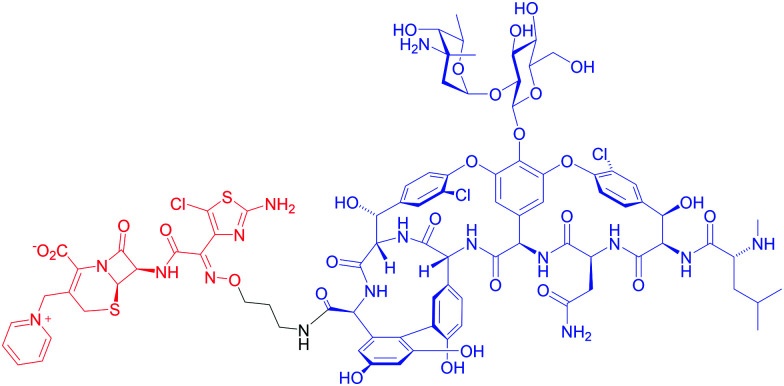
The structure of vancomycin-ceftazidime PDC TD-1792.

Peptides besides ‘standard’ cationic membrane-disrupting AMPs have been employed in PDCs. The conjugation of vancomycin to other peptides has already been discussed in this section, but it can also act at the peptide component of a PDC. TD-1792 is a conjugate of vancomycin and the cephalosporin ceftazidime, which both target different components involved in cell wall biosynthesis (Fig. 28). TD-1792 displays activity against MDR Gram-positive bacterial strains that cannot be achieved with a physical mixture of the individual drugs.^484^ Developed by Theravance Biopharma, TD-1792 completed Phase II clinical trials for the treatment of Gram-positive skin infections in 2007, but no clinical trials have been listed since.^491^

Other established classes of antibiotics have been employed in conjugation strategies, with mixed results. For example, Toth and co-workers investigated conjugates of fluoroquinolone levofloxacin with either the AMP indolicidin or the cell-penetrating peptide (CPP) Tat and found that a physical mixture of levofloxacin and indolicidin gave improved activity against Gram-positive bacterial strains compared to the conjugate.^492^ Li, O’Brien-Simpson, Wade and co-workers developed a SPPS approach to constructing cephalosporin–AMP conjugates with magainin II analogue MSI-78 and proline-rich AMPs (PrAMPs).^493^ Their MSI-78 conjugates displayed synergistic activity (compared to a physical mixture of the individual components) against ESKAPE pathogen *A. baumannii*, including an MDR strain.

#### 15.1.2 CPP–Drug conjugates

One approach to improving the accumulation of small-molecule antibiotics inside bacteria is the attachment of CPPs. Some CPPs translocate bacterial membranes with no bacteriostatic or bactericidal activity, others display toxicity towards the bacteria, and many CPPs sit between these two extremes in activity. When the CPP causes bactericidal activity, it can equally be considered as an AMP. The exact role of the CPP in a CPP–drug conjugate varies depending on the CPP used and the target bacteria.

**Fig. 29:**
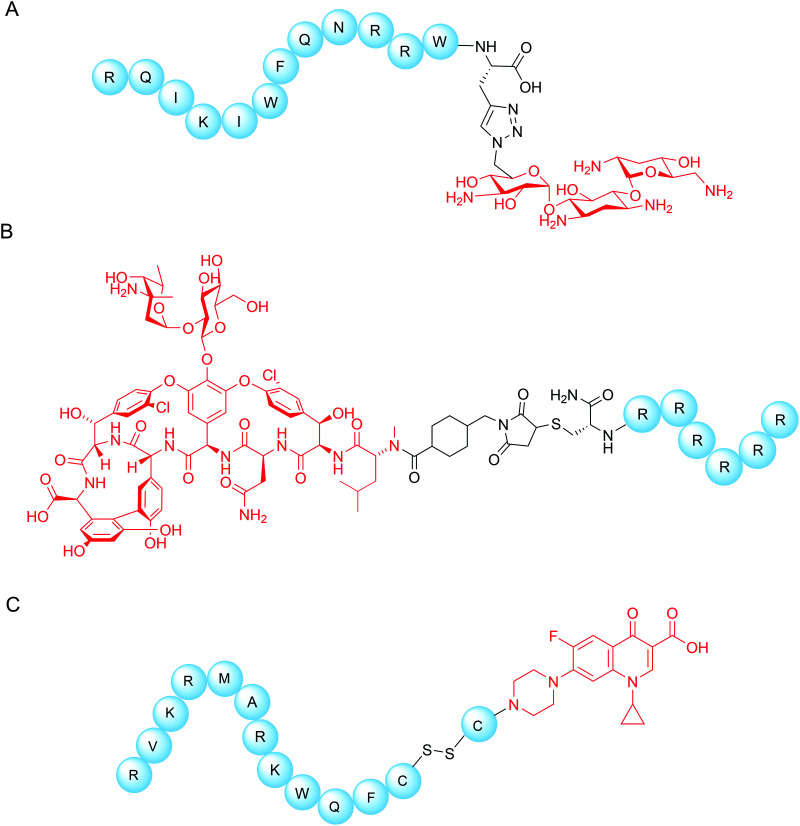
The structures of PDCs. (A) A tobramycin-penetratin PDC, (B) A polyarginine CPP-vancomycin PDC. (C) A cleavable AMP-ciprofloxacin PDC. The drugs are highlighted in red, the linkers in black, and the aa are shown as a blue sphere.

Kasko, Wong and co-workers published a CPP–drug conjugate consisting of the aminoglycoside tobramycin conjugated to the CPP penetratin, which has no antimicrobial activity and therefore, this conjugate serves only to improve the cellular uptake of tobramycin (Fig. 29A).^481^ The conjugate was active against *E. coli* and displayed significantly increased membrane disruption compared to tobramycin alone. The conjugate was also significantly more active than tobramycin against the so-called ‘persister’ *E. coli* cells. This conjugate was constructed by first synthesising azido-Boc_5_-tobramycin and attaching this to Fmoc-protected l-propargylglycine *via* a CuAAC reaction. The Fmoc-protected tobramycin-containing aa was then used in the first amide coupling step of the SPPS of penetratin.^494^

More recently, a polyarginine CPP was attached to vancomycin to restore its antibacterial activity against vancomycin-resistant bacterial strains (Fig. 29B).^495^ The lead candidate was synthesised by first derivatising vancomycin through a site-specific coupling with sulfosuccinimidyl 4-(*N*-maleimidomethyl)cyclohexane-1-carboxylate. The polyarginine CPP was then constructed *via* Fmoc-SPPS with a C-terminal Cys residue. The two components were linked by a conjugate addition reaction between the Cys residue and maleimide-derivatised drug. The PDC displayed significantly improved antibacterial activity compared to vancomycin alone and was active against several vancomycin-resistant strains. An *in vivo* study of *S. aureus*-infected mice showed that the conjugate reduced the number of colony-forming units while maintaining a stable body weight.

#### 15.1.3 Other PDCs

Unlike the previously described PDCs, where the cargo is acting as an antimicrobial agent, Devocelle and co-workers conjugated a cephalosporin moiety to the N-terminus of an AMP in a prodrug strategy.^496^ The antimicrobial activity of the AMP was linked to its overall positive charge, therefore, the authors proposed that they could modulate it by masking the cationic N-terminus of the peptide. In the presence of β-lactamase producing bacteria, the cephalosporin group would be hydrolysed and eliminated, which would result in an increased net-positive charge of the peptide, thus leading to increased antimicrobial activity. The cephalosporin–AMP prodrug was approximately two-fold more active in β-lactamase-positive *E. coli* than in a β-lactamase-negative strain, however the cephalosporin–AMP conjugate did not achieve the same activity as the unconjugated peptide. The authors hypothesised that this approach may be more effective when applied to a shorter peptide bearing a lower overall positive charge, as there would be a greater differential between the prodrug and parent peptide.

### 15.2 Cleavable PDCs

One strategy which appears in several PDCs is the incorporation of a cleavage mechanism that facilitates separation of the two components of the conjugate upon reaching a target cell. This cleavable linker typically makes use of either a reducible disulfide bond, or a moiety that can be cleaved by an enzyme, as employed in the ‘pro-drug’ strategy of Devocelle and co-workers.^496^ In a PDC consisting of an AMP and a small-molecule antibiotic, the use of a cleavable linker results in the release of the free species at the desired target, meaning that there is less chance of the chemical modification required for conjugation to negatively affect the activity of the two antimicrobial species.

An illustrative example was reported by Rolka and co-workers, who synthesised a conjugate of ciprofloxacin and an AMP based on a fragment of human lactoferrin (HLopt2), which distorts the bacterial outer membrane (Fig. 29C).^487^ The two species were connected *via* a disulfide linker, which was constructed by a solution-phase disulfide exchange between ciprofloxacin modified with a disulfide-protected Cys residue, and a Cys residue naturally present in HLopt2. The resulting PDC displayed good, broad-spectrum antibacterial activity, and no haemolysis. Further studies confirmed that the disulfide was reduced intracellularly, releasing the peptide and ciprofloxacin moieties.

### 15.3 PDCs targeting intracellular bacteria

While many bacteria exist entirely extracellularly, there are several species that must reside within a host cell for some, if not all, of their life cycle. Many of these bacteria, such as *Mycobacterium tuberculosis* (*Mtb*, causing tuberculosis (TB)), *Salmonella enterica* (causing salmonellosis, a common type of food poisoning), and *Listeria monocytogenes* (causing listeriosis), are highly relevant to human health.^497,498^ The WHO estimates that a quarter of the world's population are infected with *Mtb*, and therefore are at risk of developing TB, one of the top ten causes of death globally.^499^ Here, we will briefly describe the challenges associated with targeting intracellular pathogens and will highlight examples where AMPs have shown some promise in the treatment of *Mtb*, the archetypal intracellular pathogen.

During an infection, phagocytes engulf and eliminate pathogenic bacteria as part of the host's natural defences. Extracellular pathogens typically attempt to evade phagocytosis either by avoiding recognition altogether, or by disrupting the mechanical aspects of the phagocyte's cytoskeleton such that they are incapable of enveloping invading organisms.^497^ In contrast, intracellular bacteria capitalise on the host response in order to infiltrate phagocytic cells, which they then use to support their growth and replication. This lifestyle may also help the pathogen to ‘hide’ from other components of the host's immune response. In addition to phagocytic cells, epithelial cells are often infiltrated because they are one of the first cells an invading pathogen is likely to encounter. Indeed, bacterial pathogens typically must traverse some form of epithelium in order to establish an infection.^497^

Targeting intracellular pathogens is challenging because it requires a drug to traverse several biological membranes. For example, to reach a bacterium residing in a macrophage, the drug must cross both the macrophage plasma membrane and then the intracellular phagosome membrane before it can reach the bacterial cell envelope.^3^ Many small molecules are unable to effectively treat such infections, in part due to their poor membrane permeability and susceptibility to efflux. The challenge is further heightened because intracellular pathogens typically grow slowly, with some able to enter a dormant, non-replicative state.^500^ This limits the utility of drugs that inhibit targets that are only essential during the growth phase of the cell cycle. Many drugs are also ineffective due to the rapid development of resistance.^501^ The typical treatment for TB involves a cocktail of up to four antibiotics for at least six months, and the treatment of multidrug resistant TB can take over a year.^502^

A variety of carrier systems have been investigated for targeting antibiotics to the intracellular compartments of phagocytes including liposomes, nanoparticles, ghost cells, and polymer conjugates.^503^ Another approach is the use of cationic, broad-spectrum AMPs. In the case of TB, it is believed that AMPs interact with the mycobacterium cell envelope, although as the architecture of the cell envelope is not yet fully understood, the precise nature of this mechanism of action is unconfirmed. Well-studied AMPs such as LL-37 (cathelicidin), PG-1 (protegrin), defensins, and lactoferrin, as well as synthetic AMPs, have been shown to have anti-TB activity *in vitro*. Additionally, AMPs have been identified as having synergistic or additive relationships with traditional small-molecule anti-TB drugs.^504–506^ For an in-depth discussion of the application of AMPs as anti-TB agents, we direct the reader to the excellent review by Gutsmann.^507^

**Fig. 30:**
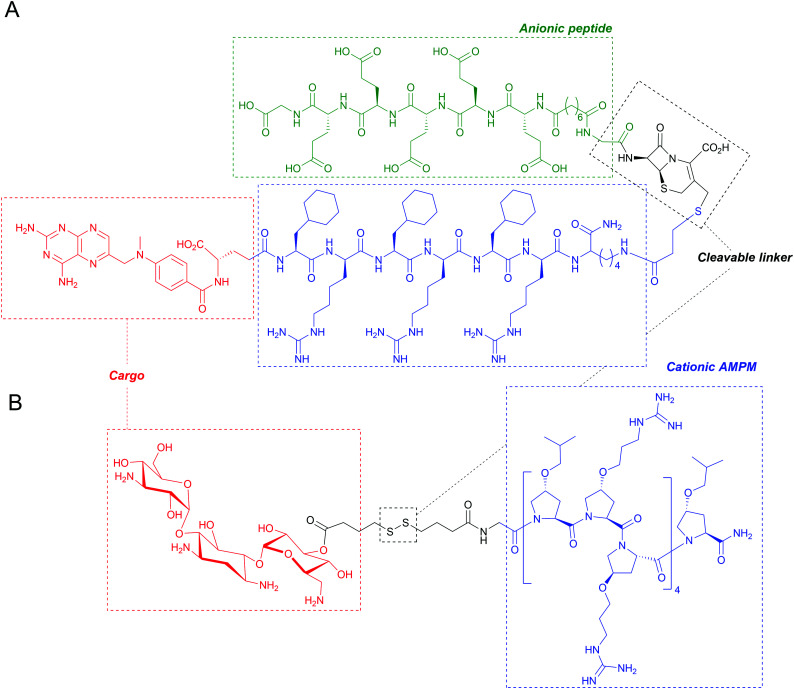
The structures of two PDCs targeting intracellular bacteria. (A) A multi-component PDC comprising of methotrexate, a cationic AMPM and an anionic peptide linked *via* a cleavable cephalosporin moiety. (B) A kanamycin–AMPM conjugate containing a disulfide linkage.

CPP–drug conjugates have already been discussed as a strategy to improve the bacterial penetration of a drug. Drugs that target intracellular bacteria must transverse both eukaryotic and bacterial membranes to reach the intended target, and therefore PDCs can be applied here as well. Kelley and co-workers developed a cleavable dual-CPP–drug conjugate for treating mycobacterial infections using a combination of SPPS and solution-phase chemistry (Fig. 30A).^485^ Methotrexate, a small-molecule dihydrofolate reductase inhibitor, has the potential to be effective against *Mycobacterium tuberculosis* infections; however, it cannot penetrate the mycobacterial cell wall. Additionally, methotrexate must enter eukaryotic cells to reach the mycobacteria. To address these issues, Kelley and co-workers attached methotrexate to a lipophilic, cationic 6-mer AMPM comprised of alternating d-Arg and cyclohexylalanine residues (called the ‘delivery peptide’). When conjugated to the ‘delivery peptide’, methotrexate was able to penetrate the mycobacterial cell wall. To enable the drug to be taken up by eukaryotic macrophages, the methotrexate-delivery peptide construct was conjugated to an anionic poly-Glu peptide, which neutralised the net positive charge of the construct and consequently promoted phagocytosis. The two peptides (delivery and anionic) were conjugated *via* a β-lactamase cleavable cephalosporin linker, so that upon successful uptake into phagocytes, β-lactamases secreted by the intracellular mycobacteria would hydrolyse the linker, facilitating an infection-activated ‘shedding’ of the anionic peptide. The methotrexate-delivery peptide is consequently released enabling it to enter the mycobacterial membrane and exert an antimicrobial effect. The conjugate displayed improved antimicrobial activity against *M. smegmatis* and *M. tuberculosis*, and reduced cytotoxicity against macrophages than methotrexate alone.

Chmielewski, Saleem and co-workers also devised a PDC that targets intracellular pathogens, conjugating aminoglycosides to an AMPM (Fig. 30B).^472^ Aminoglycosides, such as kanamycin, cannot penetrate eukaryotic cell membranes and therefore are ineffective against bacteria that can reside in macrophages, such as *Mycobacteria*, *Salmonella*, and *Brucella* spp. To overcome this limitation, the group conjugated kanamycin to a membrane-disrupting AMPM, P14LRR, either *via* a non-cleavable linker, or *via* a cleavable linker containing a disulfide bond that was reduced intracellularly, releasing the peptide and drug as separate species. The intracellular antimicrobial activity of the conjugates was assessed, and it was found that when dosed individually, the peptide and drug were incapable of clearing intracellular populations of *Mycobacteria*, *Salmonella* and *Brucella* strains from a macrophage cell line. The non-cleavable conjugate showed similar activity to a combination of the drug and peptide; however, the cleavable conjugate showed a significantly improved activity against all the bacterial strains. In *M. tuberculosis*, 93% of the intracellular bacteria were cleared with the cleavable conjugate at 10 μM. The peptide was constructed *via* SPPS using unnatural Fmoc-protected aa, which were synthesised in seven steps from a commercially available proline derivative.^508^ Kanamycin was site-selectively modified with either 4,4′-dithiodibutyric acid or sebacic acid, then coupled to the resin-bound peptide which was finally cleaved from the solid support.

**Fig. 31:**
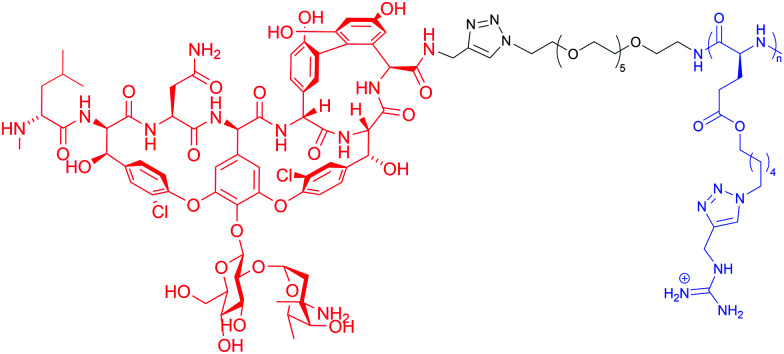
The structure of VPP-G, a PDC targeting intracellular *S. aureus* reported by Jiang and co-workers. Vancomycin is shown in red, and a membrane disruptive AMPM is shown in blue.^509^

Recent research is expanding the scope of intracellular-targeting PDCs beyond TB. Whilst usually considered an extracellular pathogen, it has been increasingly observed that *S. aureus* can invade and survive within host cells, and consequently many antibiotics commonly used to treat *S. aureus* infections (*e.g.* vancomycin, fluoroquinolones, rifampin) cannot penetrate the cell membrane and reach their intended target. Jiang and co-workers have recently published a PDC consisting of a bactericidal AMPM conjugated to vancomycin in a non-cleavable manner (VPP-G).^509^ The AMPM consists of a rigid helical backbone, and long side chains containing positively charged guanidine groups; due to its unusual architecture, this AMPM is capable of efficient membrane penetration. The AMPM backbone was synthesised by ROP, and featured a pendent C-terminal azido group, to which propynyl-functionalised vancomycin was attached *via* CuAAC. The AMPM side chains were functionalised by conversion of the pendent chloro groups to azido groups and subsequent CuAAC to attach 2-propynylguanidinium, yielding the desired PDC (Fig. 31). Whereas unconjugated vancomycin loses 1000-fold potency against intracellular *S. aureus* (IMBC_99.9_ > 710 μM, where IMBC_99.9_ is the minimum bactericidal concentration to kill 99.9% of intracellular bacteria) compared to planktonic *S. aureus*, VPP-G had an IMBC_99.9_ = 9 μM (a physical mixture of the AMPM and vancomycin had an IMBC_99.9_ = 95 μM). VPP-G was also active against MRSA and VRE. Microscopy studies with labelled VPP-G confirmed that the PDC penetrates macrophages and co-localises with intracellular *S. aureus*; staining evidence suggested that this occurred *via* direct membrane penetration. Finally, the authors used SEM to confirm that cell wall damage occurred when *S. aureus* cells were treated with VPP-G, confirming a dual-mechanism of action wherein the AMPM caused membrane disruption, and vancomycin inhibited cell wall biosynthesis.^509^

Novel and effective treatments are needed to treat infections caused by intracellular pathogens. It is promising that the use of AMPs in this field is growing, however, further research is required. In the context of TB, Gutsmann highlights that a great deal of additional information is necessary due to the complexity of the mycobacterial cell envelope and the slow growth kinetics of *Mtb*.^507^ In order to successfully apply AMPs as novel therapeutics, we therefore need to better improve our understanding of the mechanism of action of AMPs as antimicrobials against intracellular pathogens.

## 16 AMPs as antifungal, antiviral, and antiparasitic peptides

In line with available literature, this review has so far mainly focussed on the antibacterial properties of AMPs. In this section, we will draw particular attention to the antifungal, antiviral, and antiparasitic activity of AMPs, which is often overlooked.

### 16.1 Antifungal peptides (AFPs)

Fungi and yeast are eukaryotic organisms that belong to the fungus kingdom and are multicellular and unicellular respectively. Fungal cells are typically larger than bacterial cells, and, crucially for their role as a therapeutic target, contain a cell wall which has a distinct composition compared to bacterial cell walls. The fungal cell wall contains chitin, glucans, and glycoproteins, with the exact structure and composition varying between different fungal strains. Fungal infections, also known as mycoses, range in severity from common ailments such as fungal nail infections and thrush, to diseases like invasive candidiasis that can be extremely harmful or even life-threatening.^510^ Many fungal infections are never diagnosed; however, it has been estimated that in the US in 2017, the treatment of diagnosed fungal infections costs 7.2 billion USD.^511^ The main human pathogenic fungal species are *Candida* spp. (*e.g. C. albicans*), *Cryptococcus* spp. (*e.g. C. neoformans*), and *Aspergillus* spp. (*e.g. A. fumigatus*) which are responsible for over 90% of deaths due to invasive fungal infections.^510^ Typical treatment for fungal infections involves the use of small-molecule drugs such as fluconazole. However, as with bacterial infections, resistance towards these drugs is increasing. For these reasons, antifungal peptides (AFPs) are being explored as potential new antifungal therapeutics.

There are examples of peptides that display antifungal activity as part of a broad spectrum of antimicrobial activity, and peptides that are selective for fungi. Peptides that display antifungal activity alongside other microbes include AMPs that are typically short, cationic, and amphipathic (see Section 4). These AMPs typically exert their effect by either directly targeting the invading pathogen, or by stimulating an inflammatory response (see Section 6.2).^510,512^ AMP families such as the magainins, cathelicidins and lactoferrins display general antimicrobial activity that includes antifungal activity. However, many papers describing novel AMPs focus on their antibacterial properties and do not screen against fungal strains. Therefore, this activity is often overlooked.

Peptides that display only antifungal activity are structurally distinct from broad-spectrum AMPs and have specific mechanisms of action including inhibition of DNA, RNA, and protein synthesis; DNA or RNA binding; membrane permeabilisation; inhibition of cell wall synthesis; induction of apoptosis; and repression of protein folding and metabolic turnover.^510^ These AFPs range in size and structure from small species such as the nikkomycins, to plant defensins such as NaD1 which lie on the border of what can be considered a peptide (MW ∼ 5 kDa). Out of 3230 peptides in the APD3, 1199 were reported to have antifungal properties.^50^ Similarly to AMPs, AFPs can be natural products, they can be designed *de novo*, or identified *via* the screening of large libraries.^510^

A non-exhaustive list of the major mechanisms of action that natural product AFPs can display is given in Table 6. This information is drawn from several excellent reviews.^387,510,513^ Some AFPs inhibit biosynthesis of 1,3-β-glucan, a polysaccharide which is highly abundant in the fungal cell wall. Inhibition of 1,3-β-glucan synthesis destabilises the cell wall, increasing susceptibility to osmotic stress and ultimately leading to cell death. Examples of AFPs that act in this way include the echinocandin family of cyclic lipopeptides.^514^ Chitin is another primary component of the fungal cell wall which significantly aids its structural integrity. The nikkomycins and polyoxins, which are both peptide nucleosides, are key families of chitin biosynthesis inhibitors.^513^ Aureobasidins, cyclic lipophilic depsipeptides, disrupt the assembly of chitin in the cell wall.

**Table 6:** A non-exhaustive list of natural product antifungal peptides

Mechanism of action	Specific examples
Cell wall synthesis inhibitors	Echinocandins,^514^ cyclic lipopeptides
	*E.g.* Pneumocandin B_0_
	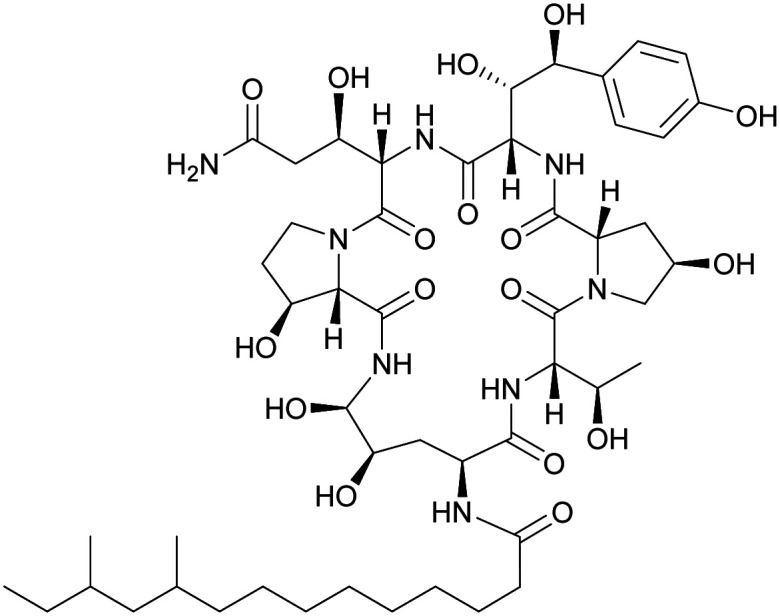
Cell wall synthesis inhibitors	Aureobasidins, cyclic, lipophilic peptides
	*E.g.* Aureobasidin A
	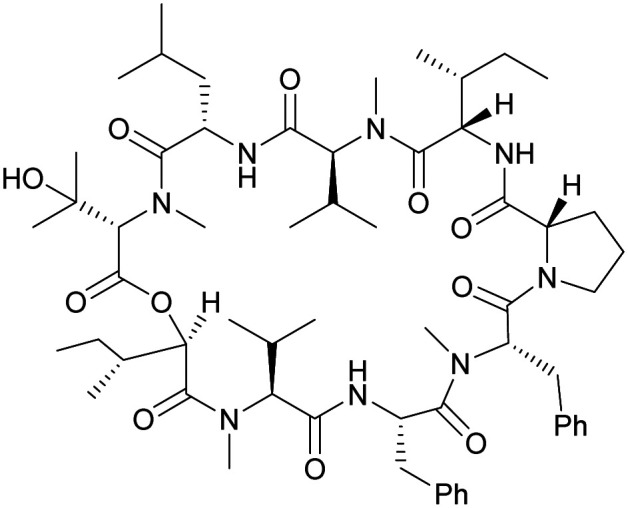
Cell wall synthesis inhibitors	Nikkomycins: structural analogues of uridine diphosphate N-acetylglucosamine
	*E.g.* Nikkomycin Z
	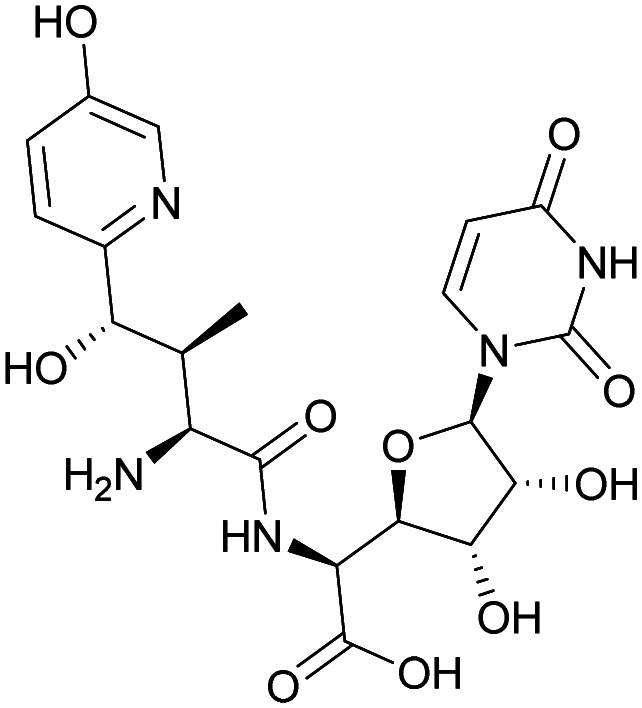
Selective antifungal cell membrane disruption	Defensins *E.g.*NaD1
Selective antifungal cell membrane disruption	Histatin 5: α-helical linear peptide (sequence: H-DSHAKRHHGYKRKFHEKHHSHRGY-OH)
Selective antifungal cell membrane disruption	Iturins: cyclic peptides with a lipophylic β-amino acid linked to a d- and l-aa
	*E.g.* Iturin A
	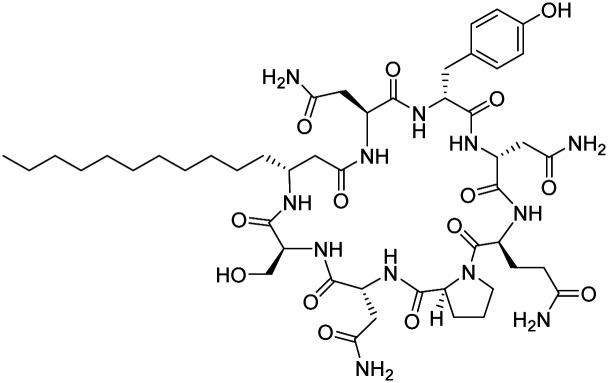

Finally, some AFPs are capable of selectively disrupting fungal cell membrane components. Defensin Rs-ARF2 interacts with glucosylceramide, which leads to increased membrane permeability. Additionally, Rs-ARF2 causes the production of reactive oxygen species within the fungal cell. Due to the selective mechanism of action, Rs-ARF2 and closely related analogues (NAD1, Rs-ARF1, SPE10) are not toxic to mammalian cells. Iturins, cyclic peptides that contain a fatty-acid β-aa, cause pore formation and subsequent leakage of ions.^515^ Histatin 5 is an α-helical linear peptide which can bind to Ssa2p, a cell wall protein, and is then transported into the fungal cells, where it acts on mitochondria. While the vast majority of the AFPs discussed in this section are natural products, there are synthetic peptides and peptidomimetics that display antifungal activity, several of which are listed by Sardari and co-workers.^516^ Arendt and co-workers reported a *de novo* designed peptide, KK14 (sequence H-KKFFRAWWAPRFLK-NH_2_) and related analogues, which inhibited the growth of food spoilage fungi.^517^ The authors do not comment on the mechanism of action of these peptides. According to a recent review of AMPs in clinical trials by Koo and Seo, two AFPs have entered clinical trials.^518^NP213, a cyclic Arg_7_ peptide, has recently completed phase IIa clinical trials for onychomycosis (a fungal nail infection), and targets the fungal membrane.^519^CZEN-002 is a dimeric octamer with a disulfide linkage used for the topical treatment of vulvovaginal candidiasis. The peptide is derived from α-melanocyte-stimulating hormone (α-MSH) with immunomodulatory activity that is exerted by disrupting intracellular signalling through cAMP accumulation in the fungal cells.^518^ In 2016, CZEN-002 was reported to have entered Phase II clinical trials; however, no updates have been published since.^513^

Antifungal AMPs have also been used in combination therapies which have the advantage of reducing the dose of both drugs, lowering unwanted side effects, and reducing the likelihood of resistance emerging. For example, the AMP lactoferrin was shown to have a synergistic relationship with fluconazole, a small-molecule antifungal agent.^520^

### 16.2 Antiviral peptides (AVPs)

Viruses are non-cellular pathogens, which must enter a host cell to survive and replicate. Viral infections are responsible for human diseases such as influenza, hepatitis, Ebola, and acquired immunodeficiency syndrome (AIDS). Historically, viral infections have been devastating for human populations. For example, the variola virus responsible for smallpox killed three out of 10 infected people until its eradication by vaccination in 1975. As human communities have become increasingly mobile, the threat of viral pandemics is likely to rise, as has been evidenced with the onset of the COVID-19 pandemic in 2020. For an excellent review on AVPs, including a discussion of peptides with antiviral activity against COVID-19, please see Afshar and co-workers.^521^ These recent events have thrust into the public eye the need for scientists to be able to rapidly develop effective antiviral treatments.

A virus particle, or virion, contains genetic information, either in the form of single or double-stranded RNA or DNA, contained within a protein capsid (together called the nucleocapsid), which often contains viral enzymes as well. A lipoprotein membrane containing viral proteins sometimes surrounds the nucleocapsid and is referred to as the envelope.^2^

**Fig. 32:**
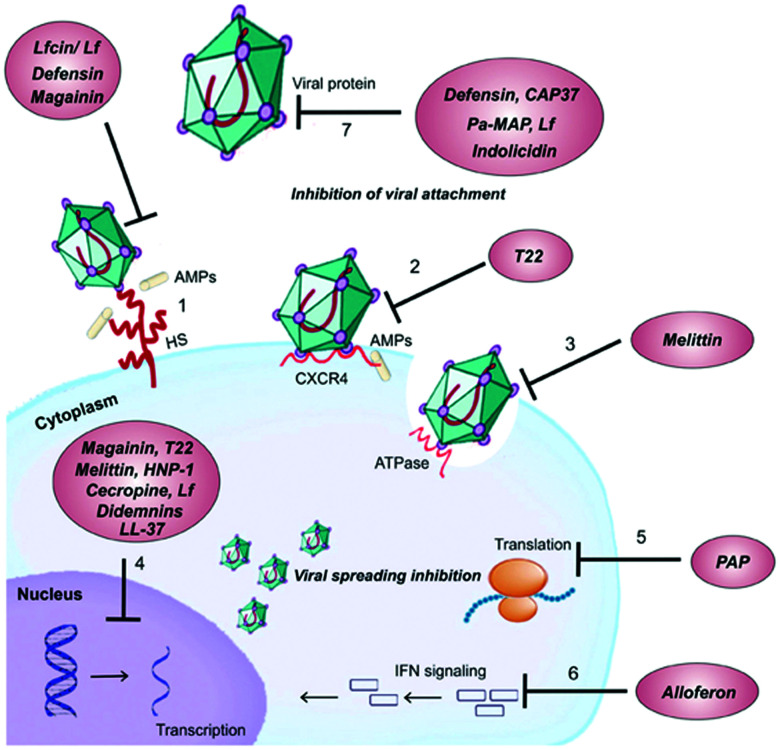
A cartoon depicting the mechanisms of action of cationic AVPs. Figure reproduced from Mulder *et al.*, *Front. Microbiol.*, 2013, **4**, 1–23, with permission from Frontiers, Copyright (2013).^522^

For many AVPs, the exact route by which they inactivate viruses has not been fully elucidated. Possible mechanisms include inhibition of viral attachment, binding to viral targets on the host cell surface, and targeting viral proteins (blocking viral fusion and preventing entry into the host cell).^522^ AVPs can act intracellularly and inhibit viral spreading *via* the suppression of viral gene expression, inhibition of translation, or by immunomodulatory activities. It is also likely that an AVP is capable of multiple different mechanisms of action, which may vary depending on the structure of the virus in question. Finally, AVPs can induce pore formation in some enveloped virions, or cause the aggregation of viruses (Fig. 32).^523^ Given their role in the mammalian innate immune response, it is unsurprising that some AMPs display antiviral activity. The first instance of this was reported in 1986, wherein a member of the defensin family, human neutrophil peptide (HNP-1), was found to be able to inactivate various viruses including Herpes simplex and influenza.^524^ Examples of AMP families displaying antiviral properties include the defensins, cathelicidins, and transferrins among many others.^522,525,526^ It has also been suggested that the defensins can be employed as a prophylactic measure against viral infections.^527^ For discussion of short, cationic AMPs as antiviral agents, we direct the reader to a review by Narayanan and co-workers.^525^ More structurally complex peptides also display antiviral activity. **Kalata B1**, a plant-derived cyclotide displays anti-HIV activity by inhibiting fusion of the virus with the host membrane, and destroying the virus prior to cell entry *via* envelope disruption.^528,529^

Similarly to AMPs, peptides that are specifically antiviral are often derived from the sequences of viral proteins (‘templated-sequence design’), designed *de novo*, are peptidomimetics of natural peptide analogues, or are identified from library screens. Selected examples are discussed below, highlighting the different approaches researchers have used for the development of synthetic AVPs.

One approach is to use a template-sequence design guided by the sequence of a viral protein domain. Liu, Jiang, Zhong and co-workers used a heptad repeat design approach inspired by the coiled-coil domains of viral fusion proteins, and synthesised α-helical lipopeptides based on the template sequence Ac-(X_a_E_b_E_c_X_d_Z_e_K_f_K_g_)_5_-βAla-K(C16)-NH_2_, where X represents a hydrophobic aa residue, and Z represents a charged aa residue.^530^ The peptides disrupted the fusion of the virus and target cell membrane. One of the analogues displayed activity against influenza A and B strains, and a high potency against a Middle East respiratory syndrome–related coronavirus (MERS-CoV) infection.

Buckheit and co-workers developed various peptidomimetics based on a β-hairpin-containing peptide GLR-19, which was itself based on natural peptide thanatin.^526^ The authors investigated different loop sizes, and identified an analogue GLRC-2 which had improved anti-HIV activity and increased resistance to proteolytic degradation than GLR-19.

**Fig. 33:**
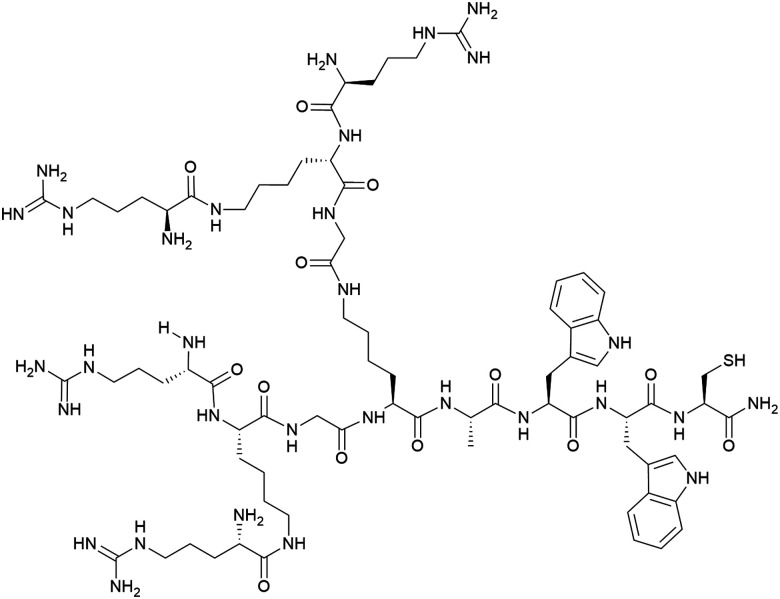
The structure of an antiviral peptide dendrimer.

Khaitov and co-workers developed peptide dendrimers (PDs, see Section 11.1) to treat respiratory syncytial virus (RSV).^531^ Two of the PDs displayed improved activity over cathelicidin-related peptide LL-37 (example shown in Fig. 33), and molecular docking studies indicated that the PDs interact with RSV cellular receptor nucleolin, however further studies are required to confirm the mechanism of action for the PDs.

Finally, library screening has been employed to identify AVPs. Tordo and co-workers provide an excellent review of phage display AVP libraries, through which AVPs have been discovered which are active against hepatitis B virus (HBV), hepatitis C virus (HCV) and HIV.^532^ The authors highlight that phage display is underused in the development of AVPs for ‘neglected’ diseases caused by viruses, such as rabies and Rift Valley fever.

There are AVPs both on the market and in clinical trials. Enfuvirtide is a marketed antiviral 36-mer (sequence: Ac-YTSLIHSLIEESQNQQEKNEQELLELDKWASLWNWF-NH_2_) that acts as an HIV-1 fusion inhibitor and is typically used in combination with other antiviral drugs. Enfuvirtide is derived from the sequence of the C-terminal helical heptad repeat region of gp41, a subunit of the viral envelope protein complex. Sifuvirtide is an analogue of enfuvirtide that has shown improved efficacy, and is currently undergoing clinical trials.^533,534^

### 16.3 Antiparasitic peptides (APPs)

A parasite is an organism that depends on a host organism for survival and replication, causing harm to the host. In 2018, there were an estimated 228 million cases of malaria, a life-threatening disease caused by parasites of *Plasmodium* spp.^535^ In addition to well-known examples such as this, neglected tropical diseases due to parasites such as lymphatic filariasis, onchocerciasis, schistosomiasis, trypanosomiasis, and leishmaniasis affect over a billion people globally. Parasitic infections, and their associated diseases, typically have a greater effect on those in lower-income countries.^536^ The potential antiparasitic properties of AMPs have been even less explored than their antifungal and antiviral properties. However, there are various natural and synthetic APPs that have been reported in the literature.^537^ Below is a non-exhaustive discussion of recently reported APPs. An excellent review of both natural and synthetic APPs is also given by Mor.^538^

Natural AMP families including the magainins, defensins, and cecropins, as well as synthetic hybrids of melittin and cecropin, have been shown to display antiparasitic activity. The targeting of intracellular parasites presents several challenges, much like the targeting of intracellular bacteria (see Section 15.3). Polycationic dermaseptin AMPs can kill intraerythrocytic malaria parasites by disrupting the host cell membrane. It has been shown that truncated dermaseptin analogues can increase antiparasitic activity with a concurrent decrease in haemolysis.^539^ Possible mechanisms of action include selective host membrane disruption, as infected erythrocytes have an altered membrane composition compared to uninfected cells, allowing them to be selectively targeted by AMPs.^538^ Additionally, AMPs have been reported as targeting parasitic cell membranes, mitochondria, or nucleic acids.

**Fig. 34:**
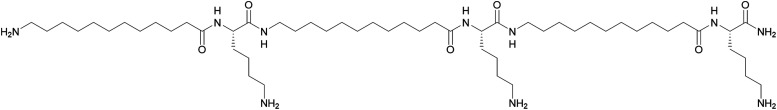
The structure of an oligo-acyl-lysine peptidomimetic based on dermaseptin **S3**.

In addition to naturally occurring peptides, numerous synthetic peptides have been developed which have antiparasitic activity. Mor and co-workers synthesised oligo-acyl-lysyls (OAKs), as peptidomimetics of dermaseptin S3 (sequence H-ALWKNMLKGIGKLAGK-NH_2_) to treat *Plasmodium falciparum*, the most virulent malaria parasite (Fig. 34).^540^ Some of the OAKs displayed high selectivity for antiparasitic activity over haemolysis. For example, OAK-2 has an IC_50_ of 0.08 μM and a selectivity ratio of >1000. The authors proposed that the mechanism of action mimicked that of dermaseptin S3, namely through permeabilisation of the parasite membrane, causing disruption of the membrane potential and K^+^ gradient, however, further studies are required to confirm this.

Finally, it has been demonstrated that APPs are highly useful as chemical probes to identify novel therapeutic targets. Tate and co-workers developed peptide-based probes of Myosin A (MyoA), a component of the *Plasmodium* glideosome, which is thought to be essential for the invasion of red blood cells by *Plasmodium falciparum*.^541^ Truncated, fluorescent MyoA peptides were developed to inhibit the protein–protein interaction (PPI) between MyoA and the MyoA tail interacting protein, allowing the authors to study how the inhibition of this PPI affected the parasites through western blot and chemical proteomic analysis. While further studies are required to confirm target engagement, it highlights the ability of peptides, in combination with modern biochemical analyses, to further improve our understanding of deadly parasites and ultimately develop novel treatments.

To date, AMP development has primarily focussed on antibacterial properties, however, there is significant potential for the development of AMPs against all types of microbial infection. For many existing AMPs, our knowledge of their activity against non-bacterial microbes is largely unexplored and this represents a vast pool of potential therapeutics against a wide range of life-limiting illnesses. Furthermore, there is significant scope for the development of novel AMPs to combat diseases caused by these organisms. In this section, several studies have highlighted the successful development of AMPs against, fungi, viruses, and parasites. Both broadly antimicrobial peptides and those that are specifically antifungal, antiviral, or antiparasitic merit further investigation to help alleviate the significant impact that these microbes exert on world health.

## 17 Conclusion and future directions

Despite the increasingly urgent antibiotic resistance crisis, pharmaceutical companies are wary of investigating and developing new antibiotics. Only a few drugs are currently available to treat infectious diseases caused by resistant ‘superbugs’, such as colistin, the well-known last-resort antibiotic, despite its known nephrotoxicity in humans.^542,543^ However, colistin could lose its effectiveness soon, as the first plasmid-mediated polymyxin-resistance gene (MCR-1) was reported in 2016, forcing the world to confront this antibiotic resistance crisis.^163,544^ As we are faced with the prospect of a world without effective antibiotics, the search for new therapeutics as alternatives to conventional drugs is imperative.

Significant research has been conducted into many aspects of AMP development, and as the isolation of nisin was in the 1920s, it has been widely debated as to whether AMPs can be considered as ‘new’ anti-infective drugs.^543,545^ The development of new AMPs requires a multidisciplinary environment, involving several areas of research such as microbiology, medicinal chemistry, synthetic chemistry, and preclinical development studies. If such collaborative and multidisciplinary work can be facilitated, we believe the development of AMP-based antibiotics effective against infectious diseases may be achieved.

So far, due to their toxicity, most commercial AMPs are applied topically, rather than *via* systemic administration, as exemplified by the polymyxin family. Although there is room for improvement in the current methods and technologies, as we have shown in this review, rational design, structure–activity relationships, and computational methods have been useful in overcoming certain limitations of natural AMPs, such as reducing toxicity and haemolytic activity, and in many cases showing superior killing of MDR bacteria and clinical isolates *in vitro*. Throughout the review, we have also highlighted the multifaceted nature of AMPs and we believe that AMPs have great potential as an alternative therapeutic option to conventional drugs to treat infections, a belief that is strongly supported by the many positive results shown in this review.

For the development of AMPs as anti-infective drugs, rational design should take into consideration several parameters, including environmental factors (pH and ionic strength), peptide length, net charge, hydrophobicity, stereochemistry, and topology. All these parameters can strongly influence the antimicrobial activity, mammalian cell toxicity, haemolytic activity, and immunomodulatory activity of AMPs.

In this final section, we would like to highlight where we believe AMPs could show particular promise as next-generation anti-infective drugs to combat AMR.

### 17.1 STAMPs

There are huge advances to be made in the translation of AMPs into effective antimicrobial therapeutics in the clinic. Whilst much of this is beyond the remit of the organic chemist, the ability to rapidly generate synthetic analogues of AMPs in order to modulate their activity and properties is a key aspect. We therefore anticipate that advances in organic synthetic techniques, for example late-stage functionalisation, will no doubt be beneficial to the field of AMPs and AMPMs. To date, only a narrow selection of possible organic transformations has been explored. In the context of AMPMs, in particular STAMPs, for example only ring-closing metathesis has been widely explored as a stapling technique. Each stapling technique brings unique properties, and therefore it is paramount that these are explored (see Section 14.1).

### 17.2 Combination therapy and AMP–drug conjugates

Due to the unique properties and activities of membrane-disrupting AMPs, they have shown promise as complementary therapeutics in combination with other antimicrobials. Reports of synergistic combinations of AMPs are sporadic and therefore it would be desirable to have more systematic studies.

In addition to combination therapy, AMP–drug conjugates offer an exciting new mode of treatment. Literature conjugates can fail to perform better than physical mixtures of the individual components, and therefore it may be that the conjugation is negatively affecting their activity. We believe that to fully benefit from the conjugation of AMPs and antibiotics, we must thoroughly explore cleavable linkers for antimicrobial purposes. Rational design based on the target bacteria and the known mechanism of action of both the AMP and drug should influence the conjugate design. The use of advanced organic synthetic transformations will provide more unusual sites of conjugation for the peptide. The application of elegant cleavable technology will bring many benefits, including targeted delivery (and therefore fewer side effects in a clinical setting) and lower doses. Due to the presence of multiple antibacterial mechanisms of action in a PDC, they can provide a solution to the treatment of MDR or intracellular bacteria.

### 17.3 Extended scope of AMP targets

A key aspect for treatment of microbial infections is the ‘magic bullet’ effect – the ability to cause harm to only the infecting entity, and not to the host organism. Peptides are more amenable to interacting with a variety of biological structures, beyond those traditionally accessible by small molecules. Therefore, one can consider many mechanisms of action beyond the well-represented membrane-disruption mode that has been discussed widely in this review. It is possible that as technological advancements are made in structural biology and the biochemical studies of microbes, new targets will be uncovered that are more easily accessed with peptides than small molecules.

### 17.4 Efflux pump inhibitors

Bacterial pathogens express a wide range of membrane-bound transporter proteins known as efflux pumps which can actively expel diverse antibiotics from the cell (see Section 2).^546^ As such, these pumps are significant contributors to MDR and are therefore an important potential target for overcoming resistance. By inhibiting these transmembrane proteins, it may be possible to limit resistance development towards novel therapeutics, and furthermore, it may facilitate resensitisation to existing antibiotics. An example of this type of resensitisation is seen in nature, wherein plants produce antibacterial berberine alkaloids alongside an MDR efflux pump inhibitor, 5′-methoxyhydnocarpin.^547^ While 5′-methoxyhydnocarpin has no bactericidal activity of its own, it enhances the activity of berberine and other cytotoxic substrates of the NorA MDR pump.

There are two examples in the literature of using peptides to inhibit bacterial efflux pumps, both of which are from the Deber group. The first example was published in 2015 where the small multidrug resistance (SMR) efflux pump, Hsmr, was targeted.^448,449^ Hsmr forms a functional efflux pump through dimerisation mediated by transmembrane helix 4. As such, a minimal-length hydrocarbon stapled peptide based on helix 4 was designed and synthesised. The stapled peptide displayed specific inhibition of efflux activity and was also able to resensitise Hsmr expressing cells to the antibiotic-like small molecule ethidium bromide, while remaining non-haemolytic. Stapling of the peptide effectively blocked peptide degradation in human plasma and liver homogenates compared to its unstapled counterpart, providing a more favourable therapeutic index. A similar stapled peptide was developed against another SMR efflux pump, EmrE, in 2018.^450^

To conclude, this review has highlighted the diverse nature and function of AMPs, as well as their potential as next-generation therapeutics. We have demonstrated many efficient strategies for the design and synthesis of novel AMPs with improved stability and bioactivity, or with a novel mechanism of action. Additionally, this review describes various synthetic modifications that could be applied to mimic the properties of naturally occurring AMPs. While the current focus of AMPs is mainly on antibacterial peptides, AMPs can be repurposed into potential therapeutics to treat infections caused by fungi, parasites, and viruses, which include neglected tropical diseases. Finally, we have highlighted key directions for the future of AMPs. We believe that the development of novel AMPs has a role to play in future generations of ‘super-antibiotics’, which are able to overcome the AMR crisis.

## Conflicts of interest

There are no conflicts to declare.

## Supplementary Material
