# Correction: The multifaceted nature of antimicrobial peptides: current synthetic chemistry approaches and future directions

**DOI:** 10.1039/d1cs90109e

**Published:** 2021-12-22

**Authors:** Bee Ha Gan, Josephine Gaynord, Sam M. Rowe, Tomas Deingruber, David R. Spring

**Affiliations:** Department of Chemistry, University of Cambridge Lensfield Road Cambridge CB2 1EW UK spring@ch.cam.ac.uk

## Abstract

Correction for ‘The multifaceted nature of antimicrobial peptides: current synthetic chemistry approaches and future directions’ by Bee Ha Gan *et al.*, *Chem. Soc. Rev.*, 2021, **50**, 7820–7880, DOI: 10.1039/D0CS00729C.

The authors regret that the following funding information was omitted from the original article: “We gratefully acknowledge the financial support from the Swiss National Science Foundation (P2BEP2-191784) to B. H. G.”

The Royal Society of Chemistry apologises for these errors and any consequent inconvenience to authors and readers.

## Supplementary Material

